# Aurora B Inhibitors as Cancer Therapeutics

**DOI:** 10.3390/molecules28083385

**Published:** 2023-04-11

**Authors:** Antal H. Kovacs, Dong Zhao, Jinqiang Hou

**Affiliations:** 1 Department of Chemistry, Lakehead University, 955 Oliver Road, Thunder Bay, ON P7B 5E1, Canada; 2 Thunder Bay Regional Health Research Institute, 980 Oliver Road, Thunder Bay, ON P7B 6V4, Canada

**Keywords:** Aurora kinase, Aurora B, chromosomal passenger complex, Aurora B inhibitors, cancer, crystal structures

## Abstract

The Aurora kinases (A, B, and C) are a family of three isoform serine/threonine kinases that regulate mitosis and meiosis. The Chromosomal Passenger Complex (CPC), which contains Aurora B as an enzymatic component, plays a critical role in cell division. Aurora B in the CPC ensures faithful chromosome segregation and promotes the correct biorientation of chromosomes on the mitotic spindle. Aurora B overexpression has been observed in several human cancers and has been associated with a poor prognosis for cancer patients. Targeting Aurora B with inhibitors is a promising therapeutic strategy for cancer treatment. In the past decade, Aurora B inhibitors have been extensively pursued in both academia and industry. This paper presents a comprehensive review of the preclinical and clinical candidates of Aurora B inhibitors as potential anticancer drugs. The recent advances in the field of Aurora B inhibitor development will be highlighted, and the binding interactions between Aurora B and inhibitors based on crystal structures will be presented and discussed to provide insights for the future design of more selective Aurora B inhibitors.

## 1. Aurora B and Cancer

The Aurora kinases are a family of highly conserved mammalian serine/threonine kinases that are critical for maintaining chromosomal integrity during the mitotic and meiotic processes. Auroras A and B regulate mitosis, and Aurora C regulates meiosis. The Aurora gene was first identified in the 1980s, and Aurora B was discovered in 1998 during screening for overexpressed proteins in cancer. Aurora B is located on chromosome 17p13.1 and is expressed at various points of mitosis [1,2].

Cellular division is a critical process responsible for the growth of all organisms that is coordinated by a variety of regulators, such as Aurora B. Mitotically, Aurora B functions to analyze and correct syntelic microtubules and kinetochore attachments [3]. Aurora B also functions to help regulate the release of cohesin [4], which is a protein that modulates the cohesion between the sister chromatids during mitosis [5]. Non-mitotically, Aurora B has also been shown to modulate telomerase and Terf1 to maintain telomeres and non-mitotically regulate epigenetic histone H3 states, as well as modulate chromatin remodeling [6,7,8,9]. Aurora B is most active during mitosis. During early mitosis, Aurora B is found to be distributed widely across the chromosomal arm [10]. Aurora B then collects along the centromeric region at the kinetochore. It will remain in this location until the centermost point of cellular division. Once the cell splits into two identical daughter cells, migration of Aurora B to the midpoint of the central spindle takes place [11]. Furthermore, Aurora B will also migrate into the equatorial cortex [12]. As cytokinesis occurs, Aurora B will further collect into the midbody and central spindle. Aurora B has an influence on chromatin condensation. Studies analyzing Aurora B inhibitors have shown that there is a relationship between Aurora B and increased levels of chromosomal segregation disorders. This has led to the belief that Aurora B has a vital role relating to chromosomal orientation, separation, and reorganization. Aurora B has been shown to activate the spindle assembly checkpoint (SAC) through kinetochore regulation [13,14,15]. During metaphase, chromosomes attach to the microtubule spindle structure through structures known as kinetochores. When chromosomes are not correctly attached to their respective microtubule spindles, their kinetochores activate the spindle assembly checkpoint. The kinetochores will lose connection and tension with the microtubules at this point, which is the driving force that activates the SAC. Once all spindle attachments are determined to be correct, the SAC then deactivates the attachment mechanism and causes cell cycle progression to halt. The SAC will then be inactivated. This progression will reactivate the cell cycle and allow cellular division and chromosomal segregation to proceed. Aurora B can modulate these actions by regulating the checkpoint proteins known as Mps1, Mad, and Bud, respectively [16]. Additionally, Aurora B modulates substrate molecules, which in turn regulate the process of cytokinesis.

With so many integral functions relating to regulating genetic information and cellular division, it is suggestive that Aurora B would be a promising target for cancer therapeutics. Approximately 90% of human malignancies have been found to contain aneuploid cells, denoting that their cells contain abnormal amounts of chromosomes [17]. This can result from a variety of defects within mitotic checkpoints, chromosome cohesion, or the attachment of chromosomes to the mitotic spindle assembly, all of which have been found to be modulated by Aurora B. Aurora B overexpression has been implicated in a wide variety of malignancies, including prostate, liver, leukemia, and breast [18]. The overexpression of Aurora B has been found to induce aneuploidy, causing genetic instability [19]. This elicits tumorigenesis, which is what has led to Aurora kinases being classified as oncogenes [20]. In vivo and in vitro studies over the last two decades have shown that Aurora B has a significant role in tumorigenesis [21,22,23].

Aurora B has long been sought after as a target for drug development. All three Aurora kinases have been studied for their respective potential as cancer therapeutics. Ideally, when inhibiting any biological molecule, its functions must be dispensable and/or replaceable for it to be a promising target, and the null organism must be able to function normally [24]. This is especially important with inhibitors with high selectivity and potency, as inhibiting the target to the greatest extent is necessary to achieve the best results. Gene knockout studies in mice have also supported the idea that Aurora B is an attractive target for cancer therapy. Studies have shown that Aurora B is expendable, and that Aurora C can take over the function of Aurora B in early embryonic phases and development [25]. When knockout mice were used to examine if Aurora A was expendable, the opposite results were found [26,27]. A similar study showed that Aurora A is an essential component for tumor suppression and normal embryonic development [26]. This proves that complete Aurora A inhibition is likely a fatal process. Knockout studies have also been conducted using Aurora C [28]. Results like those seen in Aurora B knockout mice were also observed; Aurora B was shown to compensate for the loss of Aurora C [29]. Auroras B and C have been hypothesized to have similar functions, but this represents some of the first experimental evidence that this may be true. 

To date, many Aurora B inhibitors have been developed and studied. However, as of present, none have made it to the market, to the best of our knowledge. In the next section, the Aurora B inhibitors that have entered clinical trials will be summarized, and the newer inhibitors currently in preclinical studies will be highlighted. 

## 2. Current Status of Aurora B Inhibitor Drug Development

Currently, nearly all developed and studied inhibitors of Aurora B function as ATP-competitive inhibitors. These small molecule inhibitors inhibit the autophosphorylation of Aurora B as well as the histone H3 phosphorylation on Ser_10_ [30,31]. Of note, one non-ATP competitive inhibitor, known as SP-96 (Aurora B IC_50_ = 0.316 nM), has been reported by Lakkaniga et al. [32]. This compound has demonstrated high selectivity (>2000 fold) over FLT3 and KIT, which is hypothesized to reduce off-target toxicity. Studies have shown that concurrent inhibition of FLT3 and KIT combined with Aurora B inhibition can disrupt normal hematopoiesis, leading to increased toxicity [33]. This is further supported by clinical trial results for non-selective inhibitors, such as VX-680 and AZD1152, which both inhibit FLT3 and KIT. Thus, minimizing FLT3 and KIT inhibition should be a priority when developing effective and novel inhibitors.

The development of highly selective cancer therapeutics represents some of the exceptional progress in understanding cancer pathogenesis [34]. Most modern cancer therapeutics are developed to selectively recognize a molecular target. A variety of both Aurora B selective inhibitors and pan-Aurora inhibitors that target both Aurora A and B have been developed. Many of these inhibitors have been proven to have effective antitumor properties in both in vitro studies using cell lines and in vivo studies with murine xenografts. As of present, 59 different clinical trials have been completed/are in progress, ranging from phases I to III. However, no inhibitors have made it to the market. The first-generation Aurora B inhibitors, such as VX-680, failed due to low efficacy and high toxicity when tested in clinical trials [35]. The next generation of Aurora B inhibitors, such as SNS-314, are more specific and selective to sub-types, with the hope of eliciting improved therapeutic potential and less associated side effects. [36,37].

## 3. Aurora B Inhibitors in Clinical Trials

Aurora B inhibitors have been extensively studied in clinical trials over the past two decades, as listed in Table 1. The results of these trials suggest that selective inhibitors targeting Aurora B may be effective therapeutic strategies for cancer.

### 3.1. GSK1070916

**Figure 1:**
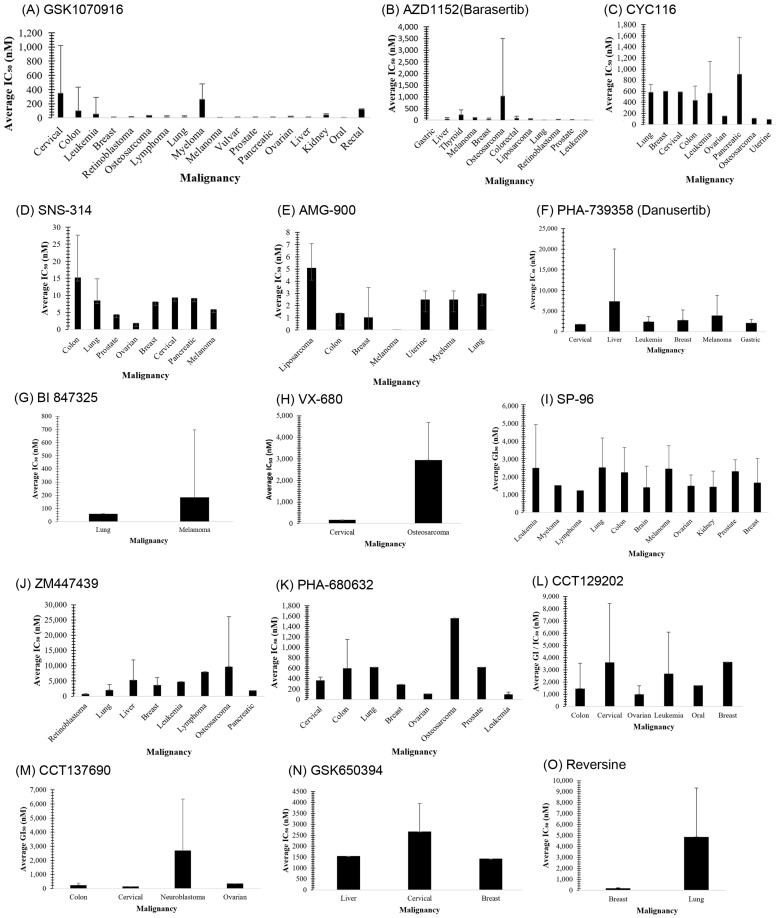
(**A**–**O**) Relationship between type of malignancy and inhibitory concentration 50% (IC_50_) of Aurora B inhibitors (indicated in the image) for a variety of cell lines analyzed when inhibited by a variety of small molecule inhibitors. IC_50_ values were taken as an average of all cell lines described in the literature per malignancy. Please refer to Table S1 for a tabulated version of the cell line and IC_50_ data. Error bars represent the standard deviation of the data.

GSK1070916 is an ATP-competitive, reversible inhibitor of Aurora B. It is an azaindole-based inhibitor. It has been shown to be an effective Aurora B inhibitor, with IC_50_ values of 0.38 and 1.5 nM for Auroras B and C, respectively [38]. It has been found to be >250-fold more selective towards Aurora B over Aurora A. In vivo studies have demonstrated an IC_50_ of 7 nM when analyzed with A549 human lung cell cancer lines [85]. A IC_50_ value of <10 nM has been observed in over 100 different human cancer cell lines (see Figure 1A), and promising tumor proliferation inhibition has been observed. In vivo studies in murine xenografts have demonstrated antitumor activity in human breast, lung, and colon cancers. The ongoing phase I clinical trial could provide further insights into the therapeutic potential of this inhibitor in treating advanced solid tumors.

Figure 1A, GSK1070916 cell lines: Cervical (*n* = 4): KB-3-1, KB-C2, HEC-1-B, and HeLa. Colon (*n* = 19): SW620 (2), SW620/AD300, MV-522, SW48, COLO 201, SW480, WiDr, COLO 205, RKO-E6, RKO, LoVo, HCT116, HT29, SW1417, DLD-1, HCT 8, COLO 320HSR, and COLO 320DM. Leukemia (*n* = 50): MEC-1, ALL-SIL, MOLT-16, HSB-2, CML-T1, MOLT-4, Jurkat, CTV-1, SKW-3, MOLT-3, CEM/CI, CCRF-CEM, JRT3-T3.5, DND-41, EM-2, EM-3, BV173, KCL-22, KU812, K-562, MEG-01, PLB-985, NOMO-1, CCRF-SB, OCI-AML-2, OCI-AML-3, ML-2, THP-1, MV-4-11, HL-60, F-36P, NB4, M-07e, OCI-M1, GDM-1, BDCM, CMK, KG-1, HEL 92.1.7, JVM-3, SUP-B15, NALM-6, SEM, RCH-ACV, CESS, Kasumi-2, TANOUE, HD-MY-Z, U-937, and JK-1. Breast (*n* = 7): SK-BR-3, MDA-MB-453, MX-1, MDA-MB-231, MDA-MB-468, MCF-7, and T-47D. Retinoblastoma (*n* = 3): Y-79, LRB1, and LRB2. Osteosarcoma (*n* = 1): U2OS. Lymphoma (*n* = 47): MJ, HuT 78, HH, KARPAS-231, L-428, TO 175.t, ST486, EB2, Raji, GA10, Daudi, Jiyoye, CA46, Namalwa, NC-37, EB1, EB3, P3HR-1, MC116, 1A2, HS-Sultan, DG-75, CRO-AP2, CRO-AP5, SR, NU-DUL-1, MHH-PREB-1, OCI-LY-19, SU-DHL-16, Pfeiffer, SU-DHL-5, SU-DHL-4, SU-DHL-6, HT, Farage, RL, DB, Sc-1, DoHH-2, Toledo, SU-DHL-10, BC-1, BC-3, BCP-1, RC-K8, BC-2, and REC-1. Myeloma (*n* = 3): RPMI8266, SKO-007, and U266B1. Lung (*n* = 6): NCI-H358, A-549, NCI-H157, NCI-H460, NCI-H1299, and NCI-H1155. Melanoma (*n* = 4): SK-MEL-2, A375-P, SK-MEL-28, and SK-MEL-5. Vulvar (*n* = 1): SW954. Prostate (*n* = 3): PC-3, LNCaP, and DU145. Pancreatic (*n* = 4): AsPC-1, Mia PaCa-2, BxPC-3, and PANC-1. Ovarian (*n* = 5): OVCAR-3, A2780, OVCAR-4, SK-OV-3, and OVCAR-8. Liver (*n* = 1): Hep-3B. Kidney (*n* = 2): A-498 and 786-O. Oral (*n* = 1): HN-5. Rectal (*n* = 1): NCI-H630.

Figure 1B, AZD1152 (Barasertib) cell lines: Gastric (*n* = 2): HGC-27 and MGC-803. Liver (*n* = 13): JHH-1, JHH-2, JHH-4, HuH-1, HuH-6, HuH-7, HLE, HLF, PLC/PRF/5, SK-Hep1, Hep-3B (2), Hep-G2, and Huh-7. Thyroid (*n* = 5): CAL-62, BHT-101, 8305C, 8505C, and TT. Melanoma (*n* = 1): MDA-MB-435. Breast (*n* = 4): MDA-MB-231, MDA-MB-361, BT-474, and MDA-MB-468. Osteosarcomas (*n* = 4): SK-ES-1, A4573, A673, and U2OS. Colorectal (*n* = 2): HCT116 and HT-29. Liposarcoma (*n* = 2): SW-872 and 93T449. Lung (*n* = 4): A549 (2), SK-MES1, and SKLU1. Retinoblastoma (*n* = 3): Y-79, LRB1, and LRB2. Prostate: (*n* = 2) LNCaP and PC-3. Leukemia (*n* = 3): PALL-2, MOLM13, and MV4-11.

Figure 1C, CYC116 cell lines: Lung (*n* = 2): A549 and NCI-H460. Breast (*n* = 1): MCF7. Cervical (*n* = 1): HeLa. Colon (*n* = 3): COLO205, HCT116, and HT29. Leukemia (*n* = 4): K-562, CCRF-CEM, MV4-11, and HL-60. Ovarian (*n* = 1): A2780. Pancreatic (*n* = 3): BxPC-3, HuP-T4, and MIA PaCa-2. Osteosarcoma (*n* = 1): Saos-2. Uterine (*n* = 1): MES-SA.

Figure 1D, SNS-314 cell lines: Colon (*n* = 2): HCT116 and HT29. Lung (*n* = 2): Calu-6 and NCI-H1299. Prostate (*n* = 1): PC-3. Ovarian (*n* = 1): A2780. Breast (*n* = 1): MDA-MB-231. Cervical (*n* = 1): HeLa. Pancreatic (*n* = 1): MIA PaCa. Melanoma (*n* = 1): A375.

Figure 1E, AMG-900 cell lines: Liposarcoma (*n* = 2): SW872 and 93T449. Colon (*n* = 1): HCT116. Breast (*n* = 40): HCC1187, MDA-MB-468, HCC38, HCC70, EFM-19, BT-20, HCC1395, MDA-MB-157, HCC1569, MDA-MB-134, UACC-893, MDA-MB-361, CAMA-1, BT-549, ZR-75-1, SUM225CWN, UACC-732, MDA-MB-415, SK-BR-3, BT-474, HCC1954, MDA-MB-436, Hs 578t, CAL-51, MCF-7, MDA-MB-231 (2), MDA-MB-453, T-47D, UACC-812, DU4475, HCC1143, HCC1937, HCC2218, KPL-1, MDA-MB-175-VII, SUM190PT, HCC1806, EFM-192A, and MDA-MB-231-PTX paciltaxil-resistant. Melanoma (*n* = 1): MDA-MB-435. Uterine (*n* = 2): MES-SA and MES-SA-Dx5 doxorubicin-resistant. Myeloma (*n* = 2): U226-B1 and RPMI-8226. Lung (*n* = 2): NCI-H460 and NCI-H460-PTX paciltaxil-resistant.

Figure 1F, PHA-739358 (Danusertib) cell lines: Cervical (*n* = 1): C13. Liver (*n* = 3): HuH-7, HepG2, and Hep3B. Leukemia (*n* = 5): THP-1, HL-60 (2), and K-562 (2). Breast (*n* = 3): T-47D, MDA-MB-231, and MCF-7. Melanoma (*n* = 5): WM3211, 1205Lu, SK-MEL-28, A375, and SK-MEL-5. Gastric (*n* = 2): NCI-N87 and AGS.

Figure 1G, BI 847325 cell lines: Lung (*n* = 1): Calu-6. Melanoma (*n* = 15): A375 (2), M229, M229R, A375R, WM793, WM793R, 1205Lu, 1205LuR, M249, M249R, WM164, WM164R, WM39, and RPMI17951.

Figure 1H, VX-680 cell lines: Cervical (*n* = 1): HeLa. Osteosarcoma (*n* = 10): U2OS, Saos-2, IOR/OS18, IOR/OS9, U2OS/DX580, Saos-2/DX580, U2OS/MTX300, Saos-2/MTX300, U2OS/CDDP4, and Saos-2/CDDP4.

Figure 1I, SP-96 cell lines: Leukemia (*n* = 4): CCRF-CEM, HL-60 (TB), K-562, and MOLT-4. Myeloma (*n* = 1): RPMI8226. Lymphoma (*n* = 1): SR. Lung (*n* = 9): A549/ATCC, EKVX, HOP-62, HOP-92, NCI-H226, NCI-H23, NCI-H322M, NCI-H460, and HCI-H522. Colon (*n* = 7): COLO205, HCC2998, HCT116, HCT15, HT29, KM12, and SW620. Brain (*n* = 6): SF268, SF295, SF539, SNB-19, SNB-75, and U-251. Melanoma (*n* = 9): LOX-IMVI, Malme-3M, M14, MDA-MB-435, SK-MEl-2, SK-MEL-28, SK-MEL-5, UACC-257, and UACC-62. Ovarian (*n* = 7): IGROV-1, OVCAR-3, OVCAR-4, OVCAR-5, OVCAR-8, NCI/ADR-RES, and SK-OV-3. Kidney (*n* = 8): 786-O, A-498, ACHN, Caki-1, RXF 393L, SN12C, TK-10, and UO-31. Prostate (*n* = 2): PC-3 and DU145. Breast (*n* = 7): MCF7 (×2), MDA-MB-231/ATCC, Hs 578T, BT-549, T-47D, and MDA-MB-468.

Figure 1J, ZM447439 cell lines: Retinoblastoma (*n* = 3): Y-79, LRB1, and LRB2. Lung (*n* = 2): A549 and NCI-H1299. Breast (*n* = 3): MCF7 (2) and T-47D. Leukemia (*n* = 1): GL-1. Lymphoma (*n* = 1): EMA. Osteosarcoma (*n* = 10): U2OS, Saos-2, IOR/OS8, IOR/OS9, U2OS/DX580, Saos-2/DX580, U2OS/MTX300, Saos-2/MTX300, U2OS/CDDP4, and Saos-2/CDDP6. Pancreatic (*n* = 1): Capan-1.

Figure 1K, PHA-680632 cell lines: Cervical (*n* = 2): C-33 A and HeLa. Colon (*n* = 3): HCT116, HT29, and LoVo. Lung (*n* = 1): A549. Breast (*n* = 1): MCF7. Ovarian (*n* = 1): A2780. Osteosarcoma (*n* = 1): U2OS. Prostate (*n* = 1): DU145. Leukemia (*n* = 2): U-937 and HL-60.

Figure 1L, CCT129202 cell lines: Colon (*n* = 7): S1, S1-M1-80, COLO205, SW620, HCT116, HT29, and KM12. Cervical (*n* = 2): KBv200 and HeLa. Ovarian (*n* = 4): MCF-7/adr, A2780, OVCAR8, and MDA-MB-157. Leukemia (*n* = 3): HL-60, HL-60/adr, and MV4-11. Oral (*n* = 1): KB. Breast (*n* = 1): MCF-7. 

Figure 1M, CCT137690 cell lines: Colon (*n* = 11): SW48, T84, SW620, LS174T, SW403, SW948, HCT 116, DLD-1, COLO320, PC/JW2, and LoVo. Cervical (*n* = 1): HeLa. Neuroblastoma (*n* = 6): Kelly, IMR-32, SHEP WT, SH-SY5Y, SK-N-SH, and SHEP. Ovarian (*n* = 1): A2780.

Figure 1N, GSK650394 cell lines: Liver (*n* = 1): HepG2. Cervical (*n* = 2): L-02 and HeLa. Breast (*n* = 1): MCF-7.

Figure 1O, Reversine cell lines: Breast (*n* = 3): 4T1, MDA-MB-231, and MCF-7. Lung (*n* = 4): A549, NCI-H1299, NCI-H1435, and NCI-H23.

### 3.2. AZD1152 (Barasertib)

Barasertib is a promising ATP-competitive Aurora B inhibitor classified as a pyrazoloquinazoline derivative that has shown potent activity against this target in various assays. Barasertib is also known by the names AZD2811, AZD1152, and AZD1152-HQPA. It was created through the optimization of the ZM447439 inhibitor. It has shown an IC_50_ of 1 nM in kinase assays and an IC_50_ of 0.37 nM in cell-free assays [86]. Binding assays have shown >1000-fold greater affinity for Aurora B as opposed to Aurora A [19,87]. The antitumor activity has been proven in multiple murine xenograft studies. Barasertib has been well studied clinically, with trials in phases I, II, and III. There has been a total of 15 clinical trials to date. The malignancy targets of interest have focused on acute myeloid leukemia (phases I, II, and III, ongoing), relapsed/refractory diffuse B-cell lymphoma (phase II, low anti-tumor response, difficulties with administration), small-cell lung cancer (phase II, recruitment phase), and advanced solid tumor malignancies (phase I, tolerable). Overall, Barasertib is a promising Aurora B inhibitor with potential for cancer therapy, and further clinical trials are needed to determine its effectiveness in various malignancies.

### 3.3. CYC116

CYC116 is an ATP-competitive Aurora B inhibitor that was designed as a pyrimidin-2-amine derivative. IC_50_ values have been reported as 19, 69, and 9.2 nM for Auroras A, B, and C, respectively [88]. One clinical trial was initiated for advanced solid tumor malignancies (phase I), but it was terminated early by sponsors due to the complexion of the necessary pharmacological studies. In vivo studies have demonstrated impressive antitumor results in multiple solid and leukemia xenograft models, as well as P388 murine leukemia xenografts grown subcutaneously as solid tumors [89]. It has been shown in HeLa cell lysates that treatment with 1.25 µM of CYC-116 for 7 h can induce complete inhibition of histone H3 phosphorylation [90]. 

### 3.4. SNS-314

SNS-314 is an ATP-competitive inhibitor of Aurora B featuring a urea moiety in its structure. It exhibits IC_50_ values of 9, 31, and 3 nM for Auroras A, B, and C, respectively [91]. In vitro studies using various cell lines (see Figure 1E) have shown its effectiveness. It has also been proven effective in vivo in an HCT116 murine xenograft [92] with dose-dependent histone H3 phosphorylation inhibition witnessed. SNS-314 has demonstrated very impressive preclinical antitumor properties and anti-proliferative effects in cancer cells. First described in 2008, it has entered one clinical trial (Phase I, advanced solid malignancies), showed good tolerance, and is prompting future studies. 

### 3.5. AMG 900

AMG 900 is an ATP-competitive phtalazinamine derivative Aurora B inhibitor with an IC_50_ value of 4 nM for Aurora B [93]. In vivo studies have shown positive results based on human tumor xenografts with proliferating murine xenograft tissues (see Figure 1H) [94]. Currently, there have been two clinical trials with malignancy targets of interest, including acute myeloid leukemia (phase I, passable results) and advanced solid tumor malignancies (phase I, tolerance). 

### 3.6. PHA-739358 (Danusertib)

PHA-739358, also known as Danusertib, is an ATP-competitive Aurora B inhibitor and a 3-aminopyrazole derivative. It has been extensively studied, with reported IC_50_ values of 13, 79, and 61 nM in cell-free assays for Auroras A, B, and C, respectively [95]. Currently, six clinical trials are underway, targeting multiple myeloma (terminated phase II), chronic myeloid leukemia (ongoing phase II,), accelerated or blast phase Philadelphia chromosome-positive acute lymphoblastic leukemia (ongoing phase I), prostate cancer (ongoing phase II), and advanced solid tumor malignancies (ongoing phase II).

### 3.7. BI 847325

BI 847325 is a dual ATP-competitive inhibitor of both Aurora and MEK kinases and a 5-alkyl indolinone derivative of an Aurora B inhibitor. It exhibits IC_50_ values of 25, 3, and 15 nM for Auroras A, B, and C, respectively [60]. In murine xenografts, oral administration daily at a dosage of 10 mg/kg has been shown to be potent in both BRAF and KRAS mutation-positive mutant xenograft models [96]. Biomarker analysis has revealed a mechanism of MEK inhibition in BRAF mutation-positive models and Aurora inhibition in KRAS mutant models. In vivo and in vitro models have shown high efficacy, as BI 847325 has proven effective in many in vitro cell line studies (see Figure 1N). Particularly, BI 847325 has proven to be the most efficacious in BRAF and KRAS mutation-positive malignancies. BRAF inhibition resistance has been shown to be overcome by BI 847325 through the mechanism of suppressing MEK and Mcl-1, which is a novel discovery. This effect has further been examined and proven in acquired BRAF mutation inhibitor resistance moles, which were shown to have a reduction in tumor volume when analyzed in in vivo melanoma models for both BRAF mutation positive and negative xenografts. One clinical trial (phase I) has commenced, targeting advanced solid malignancies.

### 3.8. VX-680 (MK-0457)

VX-680, also known as MK-0457 and Tozasertib, is a pan-Aurora inhibitor. It is an ATP-competitive 4,6-diaminopyrimidine derivative. VX-680 was first described in 2004 by Vertex Pharmaceuticals researcher Dr. Elizabeth Harrington [62]. VX-680 is >200 times more selective toward Aurora A than B. It has IC_50_ values of 0.6, 18, and 4.6 nM for Auroras A, B, and C, respectively. Of note, it also has an IC_50_ of 30 nM for FLT3. In vitro studies have shown sensitivity to leukemia, lymphoma, and colorectal cancer cells. Cellular death has been attributed to the induction of apoptosis, as has been proven with annexin-V binding assays. In vivo studies have shown promise with acute myeloid leukemia cells [97]. In an in vitro setting, using human cancer cell xenografts in naked murine animals, it has been shown that VX-680 is effective in reducing tumor sizes with a human AML HL-60 xenograft. Tumor growth reduction has been shown to be dose dependent. Antitumor activity has been shown with colon carcinoma HCT116 xenografts in nude murine animals [98]. While VX-680 is incredibly selective, it has been found to be very toxic in clinical trials. As of present, five main clinical trials have been completed, those of which are in phases I and II. These trials have been conducted with malignant targets of interest such as advanced solid tumors, leukemia, non-small cell lung carcinoma, chronic myelogenous leukemia, and leukemia lymphoblastic acute Philadelphia positive. Results have shown positive efficacy but severe toxicity, which eventually resulted in the termination of any further clinical trials and testing. During a phase I trial (NCT00111683), eight of eighteen patients with a BCR-ABL T315I mutation and chronic leukemia (*p* = 0.44) showed some hematologic response [64]. Additionally, one of three patients (*p* = 0.33) with Philadelphia chromosome-positive acute lymphoblastic leukemia was found to have achieved complete remission. VX-680 showed promising and valuable results but is no longer a drug candidate worth pursuing. Derivatives of its structure could offer promising candidates for future studies.

### 3.9. BI 811283

BI 811283 is an ATP-competitive diaminopyrimidine derivative that acts as an inhibitor of Aurora B kinase. IC_50_ values have been reported as 9 nM for Aurora B as well as 14 nM when examined in 24 different cancer lines (no raw data available) [66]. Currently, two clinical trials have been initiated for acute myeloid leukemia (phase II, no remarkable efficacy) and advanced solid tumor malignancies (phase I, limited anti-tumor activity, no further studies warranted). Thus, clinical efficacy has been very limited. In vivo studies have shown inhibited tumor progression in murine xenografts of human non-small cell lung cancer and colorectal carcinomas [99].

### 3.10. AT9283

AT9283 is a pyrazole-benzimidazole derivative and ATP-competitive pan-Aurora inhibitor with IC_50_ values of 3 and 3 nM for Auroras A and B, respectively [69]. However, AT2983 is also a potent inhibitor of many other kinases, including JAK2 (IC_50_ = 1.2 nM), JAK3 (IC_50_ = 1.1 nM), Ab T315l (IC_50_ = 4 nM), GSK3β, FGFR2, VEGFR3, Mer, Ret, Tyk2, Rsk2, and RSK3. AT9283 has also been shown to display selectivity towards 72 other kinases when analyzed against a panel of kinases [100]. Despite this, six clinical trials have been conducted with the malignancy targets of interest, including relapsed and refractory leukemia, advanced solid tumor malignancies, non-Hodgkin’s lymphoma, and multiple myeloma. However, varying results have been obtained, which suggest AT9283 does not induce significant clinical improvement and does not have any notable efficacy. 

### 3.11. MLN8237 (Alisertib)

MLN8237, also known as Alisertib, is an ATP-competitive inhibitor that displays IC_50_ values of 1.2 and 396.5 nM for A and B, respectively [101]. Most analyses of this inhibitor have focused on Aurora A, and it has been shown to induce arrest of the cell cycle, polyploidy, and apoptosis. In vitro studies have shown increased G2/M cell cycle arrest, consistent with other Aurora kinase inhibitors. Treatment with Alisertib in HCT116 cells has been linked to a larger proportion of aneuploidy due to the inhibition of Aurora B. This inhibitor has shown efficacy in multiple murine xenografts, specifically those of multiple myeloma and neuroblastoma origin [102,103]. Currently, two phase I clinical trials have been initiated, both of which target advanced solid malignancies.

### 3.12. ABT-348 (Ilorasertib)

ABT-348, also known as Ilorasertib, is an ATP-competitive pan-Aurora inhibitor that displays potent inhibition of Auroras A, B, and C, with IC_50_ values of 120, 7, and 1 nM, respectively [104]. Preclinical evaluations have demonstrated that Ilorasertib inhibits histone H3 phosphorylation. This inhibitor has been analyzed in a variety of in vitro cell lines, including solid malignancies, leukemia, and lymphoma. In vivo efficacy has also been proven in murine xenografts of MV-4-11 acute myeloid leukemia, showing significant tumor volume reductions [105]. As of present, four clinical trials have been conducted, including three phase I trials and one phase II trial, all of which targeted advanced solid malignancies for proof of concept and pharmacodynamic/pharmacokinetic analysis.

### 3.13. TAK-901

TAK-901 is an ATP-competitive pan-Aurora inhibitor with IC_50_ values of 21 and 15 nM for Auroras A and B, respectively [82]. In vitro efficacy has been proven in a wide range of cancer cell lines, with IC_50_ values ranging from 40 to 500 nM as well as EC_50_ values of 50–200 nM having been reported [82]. Specifically, the induction of polyploidy has been reported in prostate cancer (PC3) and acute myeloid leukemia cells (HL60) [106]. In vivo efficacy has been proven using HCT116-bearing murine xenografts, yielding tumor inhibition of up to 60% when analyzed with PET technology [107]. As of present, two clinical trials have commenced, both of which were phase I, with the malignant targets of interest being advanced hematologic malignancies, advanced solid malignancies, and lymphoma.

### 3.14. CS2164

CS2164, also known as Chiauranib, is a potent, ATP-competitive Aurora B inhibitor with an IC_50_ value of 9 nM [83]. CS2164 has also been shown to be a potent inhibitor of VEGFR (vascular epidermal growth factor receptor) and CSF-1R (colony-stimulating factor-1 receptor). In vitro efficacy has been demonstrated in many cell lines, namely in acute lymphoblastic leukemia and colorectal cancer [83]. As is consistent with most Aurora B inhibitors, induction of G_2_/M cell cycle arrest through Aurora B and histone H3 phosphorylation inhibition is observed. In vivo efficacy has been shown in murine xenografts bearing colorectal cancer (HCT-8) and hepatocellular carcinoma (SMCC-7721), showing tumor growth inhibition values of up to 50% [83,108,109,110]. Ten clinical trials have been initiated, ranging from phases I to III. The primary malignant targets of interest have been primarily advanced solid malignancies: small-cell lung cancer, ovarian cancer, hepatocellular carcinoma, and non-Hodgkin’s lymphoma.

### 3.15. SP-96

SP-96 is a newly discovered small molecule quinazoline derivative and Aurora B inhibitor. SP-96 is the first non-ATP-competitive inhibitor to be described. SP-96 is extremely selective, exhibiting an IC_50_ of 0.316 nM for Aurora B and a selectivity of >2000-fold over FLT3 and KIT [32]. This is thought to be incredibly important, as FLT3 and KIT inhibition combined with Aurora B inhibition have been shown to be implicated in the development of myelosuppression. As of present, all other inhibitors that have been developed inhibit FLT3 and KIT in some capacity, showing that SP-96 could have significant potential in the future of cancer therapeutics. When tested against the NCI60 cell panel, SP-96 was very potent against selected cell lines but displayed GI_50_ values > 1 µM for most cell lines. SP-96 exhibited nanomolar level values against A498 (renal), COLO 205 (colon), CCRF-CEM (leukemia), and MDA-MB-468 (breast). To date, no clinical trials have commenced for this inhibitor.

## 4. Aurora B Inhibitors in Preclinical Development

The development of Aurora B inhibitors for cancer treatment has progressed rapidly over the last several decades, with many compounds in preclinical development summarized in Table 2.

### 4.1. ZM447439

ZM447439 is a quinazoline derivative and an ATP-competitive inhibitor of Aurora B. ZM447439 exhibits IC_50_ values of 110 and 130 nM for Auroras A and B, respectively [118]. ZM447439 inhibits many other kinases, notably Auroras A and B, with >8-fold selectivity as opposed to MEK1, NMMII, and Mps1. ZM447439 has favorable anti-proliferative effects in vitro (see Figure 1F) when analyzed against a wide variety of cancer cell lines. As of present, no clinical trials have commenced, and there are no malignant targets of interest. ZM447439 has also proved successful when combined with other treatments, such as aspirin and numerous bio- and chemotherapeutic agents [119]. ZM447439 was first described in 2005, and since then, numerous small molecule inhibitors have been designed based on its structure, such as Barasertib.

### 4.2. PHA-680632

PHA680632 is a pyrrolopyrazine derivative and an ATP-competitive inhibitor of Aurora B. PHA680632 exhibits IC_50_ values of 27, 135, and 120 nM for Auroras A, B, and C, respectively [112]. When compared with a large kinase panel, PHA680632 was found to be 10–200-fold more selective for FGFR1, FLT3, LCK, PLK1, STLK2, VEGFR2, and VEGFR3 over 22 other kinases. Impressive anti-proliferative effects have been observed in vitro in several different cell lines. The efficacy of PHA680632 has also been proven in vivo using murine xenografts. PHA680632 has also been studied in combination with ionizing radiation, which was shown to increase apoptotic events and micronuclei formation when analyzed with a TP53-deficient HCT116 cell line [120]. PHA680632 was first discovered in 2006, and since then, no clinical trials have commenced, and no malignant targets of interest have been identified. 

### 4.3. CCT129202

CCT129202 is an imidazopyridine derivative and an ATP-competitive inhibitor of Aurora B. CCT129202 exhibits IC_50_ values of 42, 198, and 227 nM for Auroras A, B, and C, respectively [113]. CCT129202 has been shown to be effective in vitro (see Figure 1I). CCT129202 has been observed to be effective in vivo in murine HCT116 xenografts [121]. CCT129202 has also been shown to activate p21, a cyclin-dependent kinase inhibitor. The main function of p21 is tumor suppression, and its activation by CCT129202 could prove important in further development [122]. CCT129202 has been shown to be able to induce apoptotic events. This has been confirmed using treated cells, which showed an increase in cleaved poly (ADP-ribose) polymerase (PARP), indicative of the induction of apoptosis. As of the present, no clinical trials have commenced for this ligand.

### 4.4. CCT137690

CCT137690 is an ATP-competitive Aurora B inhibitor. CCT137690 exhibits IC_50_ values of 15, 25, and 19 nM for Auroras A, B, and C, respectively [123]. CCT137690 binds to the ATP-binding site of Aurora B. CCT137690 also contacts the glycine-rich loop when bound. CCT137690 was found to have an aqueous solubility of 0.23 mg/mL. CCT137690 has displayed impressive in vitro antiproliferative effects in a variety of cell lines (see Figure 1K). GI_50_ values have ranged from 5 to 9210 nM. CCT137690 was found to inhibit the histone H3 phosphorylation for Aurora B [114]. In murine SW620 xenografts, CCT137690 was observed to decrease tumor growth. As of present, no clinical trials have commenced for this inhibitor. Colorectal malignancies have been the focus during preclinical studies. 

### 4.5. GSK650394

GSK650394 is a newly discovered ATP-competitive small molecule inhibitor of Aurora B. GSK650394 binds to the ATP-binding pocket of Aurora B. An IC_50_ value of 5.68 µM was recorded for human Aurora B [115]. Additionally, an IC_50_ of 1.29 µM was recorded for Aurora B from *Aspergillus fumigatus* (*A. fumigatus*). When analyzing with molecular binding analyses, it was found that the carboxyl group of GSK650394 competed with the γ phosphate from ATP for the binding position at the Lys106 residue, as well as that the sizeable aromatic group of GSK650394 occupies the Aurora B hydrophobic pocket. When analyzed biologically, GSK650394 has been shown to induce cell cycle arrest during the G_2_/M phase with anticancer potential (see Figure 1L). GSK650394 is also a potent serum- and glucocorticoid-regulated kinase (SGK) inhibitor [124,125]. GSK650394 exhibits IC_50_ values of 62 and 103 nM for SGK 1 and 2, respectively. GSK650394 has also been shown to reduce histone H3 S10 phosphorylation in a variety of malignant cells. No clinical trials have commenced for this ligand.

### 4.6. Reversine

Reversine is an ATP-competitive purine derivative and Aurora B inhibitor. Reversine features a morpholine group, which phases into the solvent exposed area of the Aurora B ATP-binding pocket. Reversine has been found to inhibit a variety of molecules, including, but not limited to: MEK1, non-muscle myosin II (NMMII), Mps1 kinase, and Auroras A and B [116]. Reversine has a two-fold greater binding affinity towards Mps1 than Aurora B. In vitro, Reversine has been found to inhibit cell proliferation and induce cell apoptosis through the mechanism of modulating caspase-3 and Bax/Bcl-2 [126,127,128]. By blocking Aurora B, Reversine has been found to contribute to the anti-growth effect in BRCA and other breast cancer cell lines [129]. Additionally, Reversine has been found to be effective in triple-negative breast cancer (TNBC) cell lines. As of present, no clinical trials have commenced, and there are no malignant targets of interest. Reversine represents an interesting future possibility for an Aurora B inhibitor but is likely more suited as an Aurora A target. 

### 4.7. Hesperadin

The hesperadin molecule is an ATP-competitive Aurora B inhibitor that can be classified as indolinone-based. In autoradiography assays, it has shown an IC_50_ of 3 nM, and in cell-free assays, an IC_50_ of 250 nM [117]. Hesperadin was the first-generation Aurora B inhibitor, which shed light on the activation mechanism of Aurora B [130]. Currently, no clinical trials have been initiated, and there are no plans for further developments. However, in vivo studies have shown antitumor activity in breast cancer (MCF7) and prostate adenocarcinoma cell-based assays [131]. This relationship works by inducing cell proliferation through the reduction of Aurora B activity, causing mitotic deficiencies, and eradicating the checkpoint proteins hBUBR1 and CENP-E from the kinetochores of the mitotic chromosomes. The presence of multiple mitotic defects caused by Aurora B inhibition significantly reduces MCF7 breast and PC3 prostate cancer cell proliferation. Additionally, interesting findings were also observed in pathogenic *Trypanosoma brucei*, where Hesperadin inhibits Aurora kinases and blocks nuclear division and cytokinesis in bloodstream forms [132]. 

Several other pan-Aurora inhibitors have been described in the literature, such as MLN8054, but they are predominantly more selective for Aurora A. Quercetin, a plant flavonol, has also been characterized as an Aurora B inhibitor [133]. In vivo studies have extensively examined Aurora B inhibitors, with the degree of histone H3 phosphorylation used to assess the level of Aurora B inhibition. Barasertib and VX-680 were the first ligands examined under in vivo conditions, and Barasertib showed remarkable efficacy in murine xenografts. However, similar results have not yet been achieved in clinical studies. In vivo evidence has demonstrated that Aurora B inhibition leads to an antiproliferative phenotype [87]. Inhibition of Aurora B impairs mitotic products, leading to polypoid tumor cells that become unviable [31,134]. This differs slightly from the inhibition pattern observed in Aurora A, which forms a monopolar spindle due to irregular chromosomal segregation and delays in mitosis that result from inhibition [135]. While in vivo results have shown promise, the same level of efficacy has not yet been observed in clinical trials.

## 5. Crystal Structures and Ligand Protein Binding Interactions of Aurora B Inhibitors

Aurora kinases contain three distinct domains: the variable N-terminal domain (39–139 aa), the conserved kinase catalytic domain (250–300 aa), and the short C-terminal domain (12–20 aa) [136]. The N-terminal domain is believed to be involved in the control of protein localization and is known to provide selectivity for protein-protein interactions [137]. The N-terminal domain contains the glycine-rich loop [138,139]. The conserved kinase domain is the catalytic domain that contains the activation T-loop, which is responsible for activation of the kinase domain [140,141,142]. Structurally, the conserved kinase domain is constituted by a β-stranded N-terminal lobe and an α-helical C-terminal domain that are connected by the hinge region, which has the responsibility of maintaining the active conformation of Aurora kinases [136,143,144]. This domain also contains the ATP-binding pocket, which is hydrophobic and shaped by the adenosine residue of ATP binding in a deep left cleft that exists between the β-stranded lobe and the α-helical lobe [145,146]. The kinase domain is highly conserved amongst the Aurora proteins, with 71%, 60%, and 75% homologies between Aurora A/B, Aurora A/C, and Aurora B/C, respectively [136]. Although Auroras A and B are known to be approximately 70% identical in terms of their catalytic domain, they have very dissimilar localizations and independent functions during the cell cycle [147]. When the Aurora B: INCENP complex was superimposed with the Aurora A: TPX2 (targeting protein for Xklp2) complex, remarkable differences in conformation were observed [138,148].

**Figure 2:**
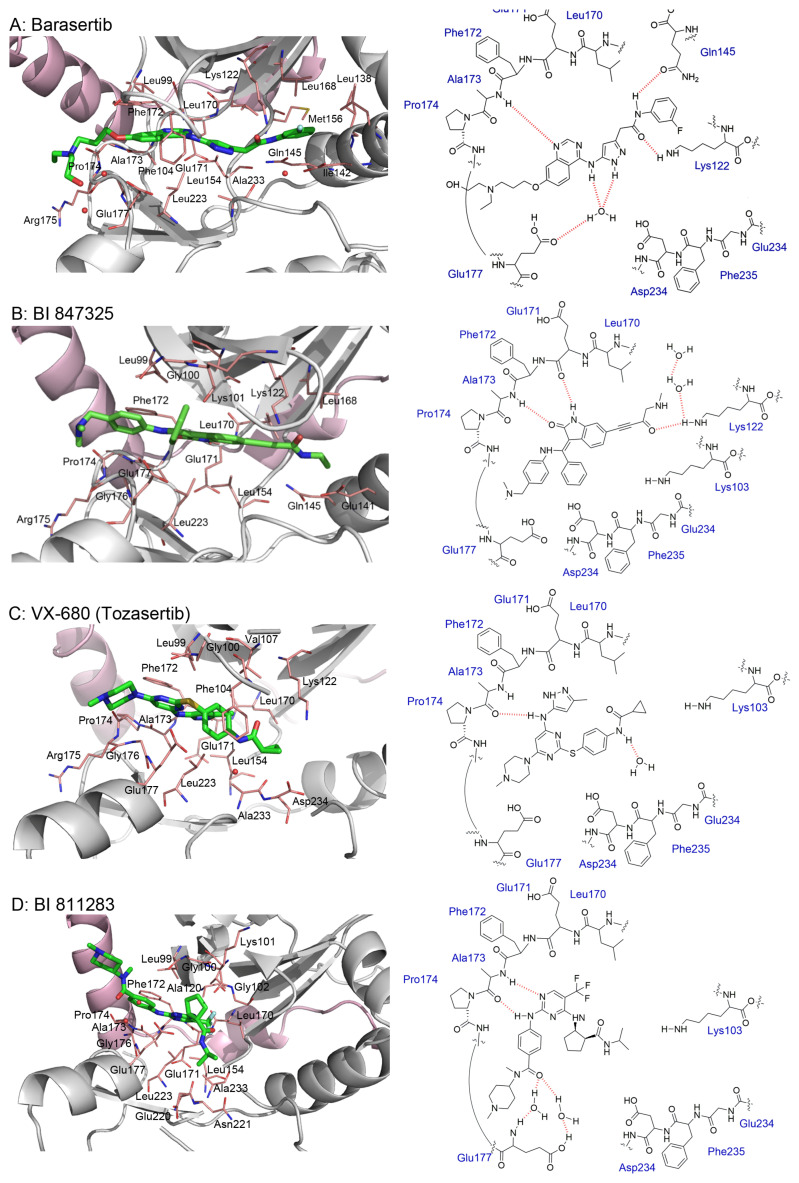

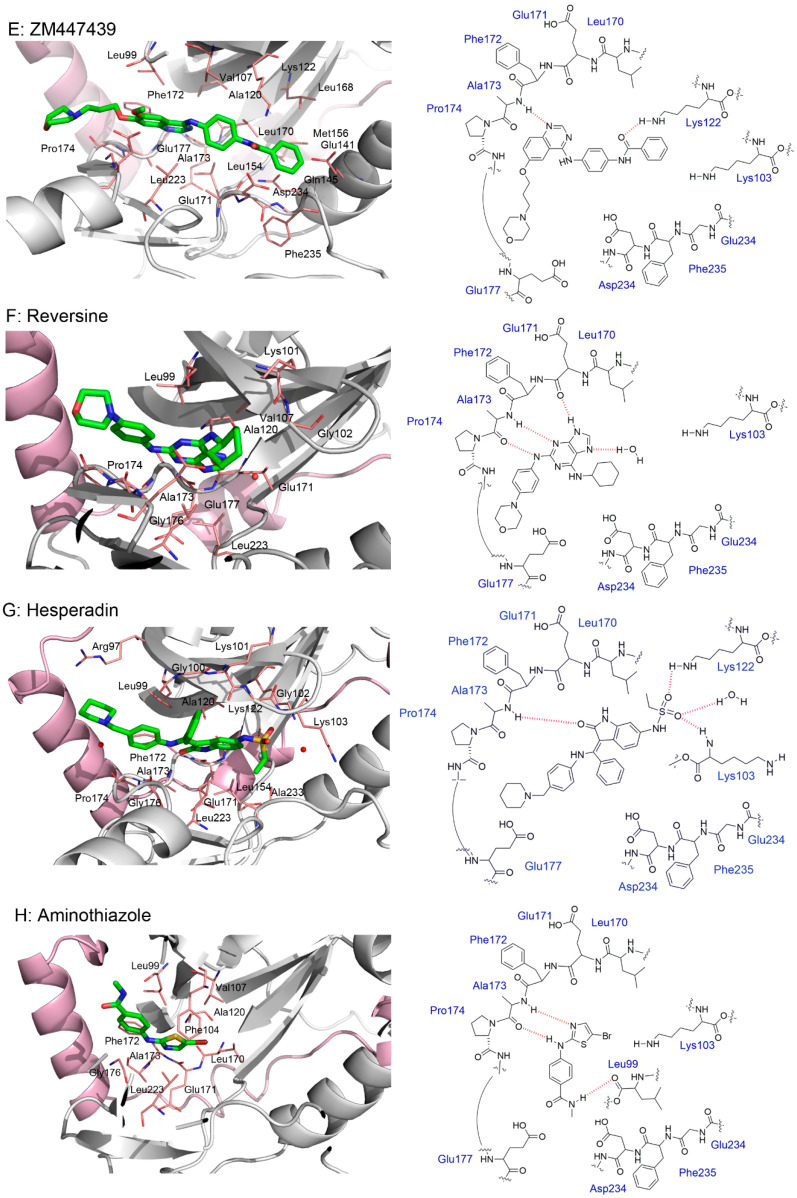
Crystal structures of inhibitors (indicated in the image) in a complex with Aurora B (**A**–**H**). Some residues have been omitted for clarity. Red dashed lines indicate hydrogen bonding interactions. See the ligand-binding interaction descriptions below for more information.

The crystal structure of Aurora B has been used to shed light on the binding interactions between Aurora B and its inhibitors, which will facilitate the development of selective small-molecule inhibitors (Figure 2). The human Aurora B crystal structure in complex with any inhibitors is still lacking in the scientific community. The crystal structure of *Xenopus laevis* (*X. laevis*) is generally used in structural biology studies. Sessa et al. first described the complex of Aurora B with INCENP and Hesperadin (PDB ID: 2BFY) within *X. laevis* in 2005 [130]. This discovery opened the door to studying the properties of Aurora B at the molecular level. It was observed that when INCENP binds, an active conformation of the T-loop is generated allosterically. This discovery has allowed for the most accurate representation of Aurora B to be used when designing new ligands.

Figure 2A: 4C2V: Barasertib [149]. Barasertib is a pyrazoloquinazoline derivative and an ATP-competitive, reversible, and selective Aurora B inhibitor. Barasertib binding does not result in any remarkable structural conformation changes. Binding occurs at the ATP-binding site and occurs across the entire pocket, spanning from the ɑC helix all the way to the hinge region. Van der Waals stacking interactions are observed between the Val_107_, Leu_154_, Leu_223_, Phe_172_, and Leu_99_ side chains. These five interactives predominantly anchor the quinazoline and anilio groups from Barasertib into place. Additionally, hydrogen bonds are observed between the amino group of the 3-fluoroaniline moiety and Gln_145_. In addition, hydrogen bonds occur between the carbonyl moiety of the 3-fluoroaniline moiety and Lys_122_. This 3-fluoroaniline moiety is grouped within Leu_138_ and Leu_168_, which form a hydrophobic pocket together, respectively. This interaction disrupts the ion pair that usually forms between the residues Lys_122_ and Glu_141_. Three water molecules facilitate the ligand-binding interaction. Two waters facilitate the binding of Glu_177_, which binds to the imidazole moiety as well as the amine group connecting the quinazoline and imidazole groups. As well, hydrogen bond formation is observed between the N-terminus amine of Ala_173_ and one of the tertiary amines of the quinazoline group. Additionally, one water molecule facilitates binding at the hydroxyl group at the solvent exposed hinge region. It appears Glu_177_ is the primary molecule responsible for the selectivity of Barasertib, with some likely secondary contributions from other Van der Waals interactions. Of note, the chain containing a hydroxyl group faces out into the solvent exposed area of the hinge region.

Figure 2B: 5EYK: BI 847325 [150]. BI 847325 is a 6-alkylindolinione derivative and an ATP-competitive selective Aurora B inhibitor. This interaction does not induce any remarkable conformational changes. BI 847325 occurs at the ATP-binding site. The indolinone moiety plays an important role in serving as the binge-binding motif. The alkynyl group binds such that it points to the DFG residue. Two hydrogen bonding interactions are observed by the indolinone group. The first between Glu_171_ and the nitrogen from the amide group, and the second between Ala_173_ and the carbonyl group. Additionally, hydrogen bonding is observed between the Lys_122_ residue and the carbonyl of the amide group that is bound to the alkynyl moiety, located deep in the ATP-binding pocket. One water molecule facilitates the Lys_122_ interaction. Of note, the tertiary amine faces the solvent exposed area in the hinge residues. It appears that the Lys_122_ interaction dictates the selectivity of the BI 847325 interaction.

Figure 2C: 4B8M: VX-680 [151]. VX-680 is an aminopyrazole quinazoline derivative and an ATP-competitive, non-selective inhibitor of Aurora kinases. Ligand binding does not induce any remarkable conformational changes. VX-680 binds at the ATP-binding site of Aurora B. The main binding affinity and selectivity are believed to be regulated by the interaction of the secondary amine from the amino pyrazole that links to the N-methyl-piperazine ring with Ala_173_ of Aurora B. Of note, the piperazine group faces out into the solvent exposed area of the hinge region.

Figure 2D: 5K3Y: BI 811283 [152]. BI 811283 is a diaminopyrimidine derivative and an ATP-competitive Aurora B inhibitor. BI 811283 binds within the ATP-binding pocket of Aurora B. Binding occurs in the hinge region. Binding does not occur as deep in the pocket as is seen with other inhibitors. Four hydrogen bonds modulate the binding. The amino group of Glu_177_ forms a hydrogen bond with the amide carbonyl that holds the aniline and piperazine groups together, and the carboxyl group of Glu_177_ forms a hydrogen bond with the same amide carbonyl. Both bonds are facilitated by two separate water molecules, respectively. Additionally, Ala_173_ forms two hydrogen bonds with the BI 811283 molecule. The amino group of Ala_173_ forms a hydrogen bond with the tertiary amino group of the pyrimidine ring, and the carbonyl group of Ala_173_ forms a hydrogen bond with the secondary amine that connects the benzene and pyrimidine molecules. Of note, the piperazine group faces out into the solvent exposed area of the hinge residues.

Figure 2E: 2VRX: ZM447439 [153]. ZM447439 is a quinazoline derivative and an ATP-competitive, selective Aurora B inhibitor. ZM447439 binds to the ATP-binding pocket of Aurora B, spanning from the aC helix all the way to the hinge region. Hydrogen bonds are formed between the tertiary amine of the quinazoline moiety and the N-terminus of Ala_173_, as well as the carbonyl of the amide group that connects the two cyclic structures deep in the ATP-binding pocket and the amino side chain of Lys_122_. Like other Aurora B inhibitors, Ala_173_ appears to modulate the selectivity of this molecule. The phenyl group faces out into the solvent exposed area of the hinge region.

Figure 2F: 2VGO: Reversine [154]. Reversine is a purine derivative and an ATP-competitive Aurora B inhibitor. Reversine binds to the ATP-binding pocket of Aurora B and is anchored through three main hydrogen bonds. Hydrogen bonds exist between the carbonyl of Ala_173_ and the primary amine that holds the 4-morpholinoaniline and purine groups together, the amino group of Ala_173_ and the tertiary amine from the purine moiety, as well as the carbonyl of Glu_171_ and the secondary amine from the purine functional group. Hydrophobic stacking interactions between the side chains of Ala_120_, Leu_170_, Ala_173_, Leu_99_, and Leu_223_ anchor the purine ring into place. Of note, the morpholine group faces out into the solvent exposed area of the hinge residues.

Figure 2G: 2BFY: Hesperadin [155]. Hesperadin is an indolinone ATP-competitive inhibitor against Aurora B, inhibiting chromosomal alignment and segregation. In the crystal structure (PDB ID: 2BFY), Hesperadin binds to Aurora B in its active conformation. The indolinone moiety of Hesperadin binds at the hinge region, with hydrogen bonding interactions between the oxygen and nitrogen groups in the moiety and the main chain carbonyl and amide of Glu_171_ and Ala_173_. In the hydrophobic back pocket, Lys_103_ and Lys_122_ dominate the major interactions through hydrogen bonding with the sulfonyl oxygen of the ligand and the main chain nitrogen of Lys_103_ and the side chain nitrogen of Lys_122_. On one end of the indolinone ring, Van der Waals interactions are observed from the principal phenyl ring of the indolinone moiety with the side chains of the residues Glu_177_, Val_107_, and Leu_99_. These interactions face the entry point of the catalytic site. Additionally, the phenylamine group is compressed between the Gly_176_ residue and the Leu_99_ sidechain. This interlinkage predisposes the piperidine group to being solvent-exposed adjacent to the ligand binding site. On the inverse side of the indolinone group, the sulfur and oxygen groups from the sulfonamide group face directly into the Aurora B active site. Two water molecules aid in facilitating the ligand binding interaction, one facilitating bonds between the carbonyl of the sulfonyl group and the secondary amine of the Lys_122_ side chain, and the other between the Pro_174_ carbonyl and the pyridine tertiary amine group. Of note, the Pro_174_ reaction is the only one that occurs in the hinge region. Additionally, a hydrogen bond is observed between the N-terminus amide nitrogen group and the nitrogen of the piperazine moiety. The Lys_122_ and Ala_173_ interactions appear to modulate the selectivity of Hesperadin. As mentioned, the piperazine group faces out into the solvent exposed area of the hinge region.

Figure 2H: 2VGP: Aminothiazole 25 [156]. This molecule, known as aminothiazole 25, is an aminothiazole derived ATP-competitive inhibitor. Aminothiazole analogs represent excellent potential for Aurora B inhibition drug candidates, as they contain the necessary hydrogen bond acceptors and donors to allow for a high degree of selectivity. Aminothiazole 25 is anchored into the Aurora B binding pocket by hydrophobic stacking interactions with the side chains of Ala_120_, Leu_170_, Ala_173_, Leu_99_, and Leu_223_. Three hydrogen bonds also facilitate the interaction, with Ala_173_ and Leu_99_. Two hydrogen bonds are formed with Ala_173_. First, the nitrogen moiety is observed to form a hydrogen bond with the amide of Ala_173_. Additionally, the amine group that connects the phenyl and aminothiazole groups forms a hydrogen bond with the carbonyl of Ala_173_. One other hydrogen bond formed between the amide of the 2-anilino phenyl ring also creates a secondary, complementary hydrophobic stacking interaction with Leu_99_. Of note, the carboxyamide amino group donates a proton to the carbonyl group of Leu_99_. It appears that in this molecule, Leu_170_ plays a role in modulating the selectivity. The carboxyamide group faces out into the solvent exposed area of the hinge residue.

## 6. Conclusions and Perspectives

The prevalence of cancer is increasing gradually, year by year. Recent studies have reported a 40.2% chance of developing cancer at some point during the human lifespan [157]. In 2018, there were approximately 17 million new cases of cancer worldwide, with approximately 9.5 million deaths being reported due to cancer [158]. With both new cancer cases and deaths expected to rise in the coming years, more cancer therapies are needed. Aurora B represents a promising target for cancer therapeutics, given that the in vitro and in vivo efficacy of many inhibitors has already been observed. Drug development efforts continue to show promise in creating a selective Aurora B inhibitor for cancer therapeutic purposes. The design of selective Aurora B inhibitors that have high tolerability is considered one of the critical factors in getting drugs to the clinic. Most clinical trials have been discontinued due to high toxicity. This was well characterized with VX-680, which has been excluded from all future clinical trials due to toxicity [159,160]. 

Inhibitors of Aurora B have been extensively pursued in the cancer therapeutic world. As of the time of this writing, 59 different clinical trials have been initiated. A few highly selective Aurora B inhibitors, such as AZD1152 and GSK1070916, have already shown promise in clinical trials, supporting the idea that selectively inhibiting Aurora B rather than all Aurora kinases represents a promising strategy in the development of anticancer therapeutics. As more selective Aurora B inhibitors make their way into clinical trials, results should continue to improve. Toxicity may be related to the combined Aurora A and B inhibition that is exhibited by most inhibitors. Due to their extreme structural similarities, designing a selective Aurora B inhibitor has proven challenging. 

The overexpression of Aurora B has been implicated as a potential contributor to chemotherapeutic drug resistance [161]. This has been demonstrated biochemically in non-small cell lung cancer models, showing that Aurora B may be implicated in cell proliferation and p53-related DNA damage pathways. This study also showed that Aurora B knockout models were able to resensitize to the same chemotherapeutic drugs [161]. Other studies have yielded similar results, specifically in ovarian carcinoma and neuroblastoma drug-resistant cells [162,163]. This evidence that Aurora B inhibition is associated with increased drug response in drug-resistant models may further demonstrate why Aurora B represents an effective drug target.

However, doubt has also been raised as to whether Aurora B inhibition will represent a promising therapeutic option. Aurora B inhibitor-induced drug response has been observed in some studies [164]. Drug resistance in Aurora kinases has been observed, which is due to Aurora B dominant point mutations [165]. Although, Aurora B inhibitor-induced drug resistance is not well understood, it was hypothesized that due to the flexibility in the Aurora B ATP-binding pocket, Aurora B may be able to form resistance to some inhibitors, depending on their exact molecular specificities, the flexibility of the ligand, and the volume/orientation of the ATP-binding pocket that the ligand occupies [165,166]. It has yet to be shown experimentally how drug resistance will affect current Aurora B inhibitors that have made their way into clinical trials. Exploiting structural changes in the ATP-binding pocket acquired due to drug resistance may even be beneficial to inhibitor selectivity and efficacy. It is likely that drug resistance will be observed at some point over the course of clinical evaluations. 

Understanding the biochemistry process of drug resistance will be essential to the development of new treatment regimens. The Auroras, and especially Aurora B, have a role in drug resistance, and further research into the molecular and genetic basis of this resistance will further our understanding of the role of the Aurora kinases in drug resistance and secondary targets. As of now, Aurora B knockout studies in drug resistance models have further validated its potential for future drug developments. While current efforts have not yielded the desired results, it is still possible to design highly selective inhibitors for Aurora B. In addition, exploitation of Aurora B mutants as therapeutic targets may be required in the quest for anticancer drugs.

It is important to note that the future of cancer therapy is likely multifactorial, and Aurora B inhibitors may prove to be effective agents in more than just the role of primary chemotherapeutics. Aurora B inhibitors have also been shown to sensitize cancer cells in the presence of different chemotherapeutic agents and radiotherapies [167,168,169]. As well, Aurora B inhibitors have been shown to help decrease resistance to radiation in some cancer cells [170,171]. 

Significant advancements have been made in validating Aurora B as a potential target for cancer therapeutics. Considering the role of Aurora B in cancer biology, this represents an inspiring prospect for anticancer agent development. Aurora B selective inhibitors have shown promise in clinical trials, and they will continue to improve as science continues to further its understanding of cancer. Given the current comprehension of the structure of Aurora B, pharmacological inhibitors that selectively inhibit the activity of Aurora B should be practicable and attainable. Discovering novel inhibitors that are highly selective, potent, and possess favorable pharmacological properties represents the task ahead. Such compounds will also serve to derive a better understanding of the biological functions of Aurora B. A more extensive understanding of the structural and molecular biology of human Aurora B will be crucial to helping pave the way for the next generation of therapeutic agents for cancer.

## Figures and Tables

**Table 1:** Comprehensive list of Aurora B inhibitors in clinical trials.

Compound Name and Structure	In Vitro IC_50_	Clinical Trial Remarks	Clinical Trials Identification Number(s)
GSK1070916 [38] 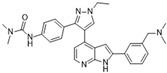	A: 490 nM B: 0.38 nM C: 1.5 nM	Phase I—Advanced solid malignancies [39]	NCT01118611
AZD1152 (Barasertib) [40] 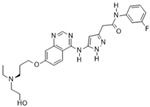	A: 1359 nM B: 0.37 nM C: n/a	Phase I—Leukemia [41]	NCT00530699
		Phase II—Lymphoma [42]	NCT01354392
		Phase I—Advanced solid malignancies [43]	NCT00338182
		Phase I—Leukemia [44]	NCT01019161
		Phase I/II—Leukemia [45]	NCT00497991
		Phase I—Leukemia [46,47]	NCT00926731
		Phase II and III—Leukemia [47,48]	NCT00952588
		Phase I—Advanced solid malignancies [43]	NCT00497679
		Phase I—Advanced solid malignancies [49]	NCT00497731
		Phase I and II—Leukemia	NCT03217838
		Phase II—Small-cell lung cancer	NCT03366675
		Phase I—Advanced solid malignancies [50]	NCT02579226
		Phase II—Small-cell lung cancer	NCT04525391
		Phase II—Small-cell lung cancer	NCT04745689
		Phase I and II—Leukemia	NCT03217838
CYC116 [51] 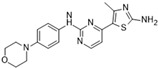	A: 19 nM B: 69 nM C: 9.2 nM	Phase I—Advanced solid malignancies	NCT00560716
SNS-314 [37] 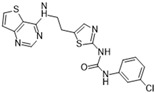	A: 9 nM B: 31 nM C: 3 nM	Phase I—Advanced solid malignancies	NCT00519662
AMG 900 [52] 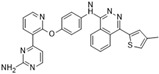	A: 5 nM B: 4 nM C: 1 nM	Phase I—Acute myeloid leukemia [53]	NCT01380756
		Phase I—Advanced solid malignancies [54]	NCT00858377
PHA-739358 (Danusertib) [55] 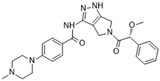	A: 13 nM B: 79 nM C: 61 nM	Phase II—Multiple myeloma	NCT00872300
		Phase II—Hormone refractory prostate cancer [56]	NCT00766324
		Phase II—Leukemia	NCT00335868
		Phase I—Advanced solid malignancies [57]	n/a
		Phase I—Leukemia [58]	n/a
		Phase I—Advanced solid malignancies [59]	n/a
BI 847325 [60] 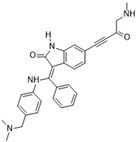	A: 25 nM B: 3 nM C: 15 nM	Phase I—Advanced solid malignancies [61]	NCT01324830
VX-680 (MK-0457) [62] 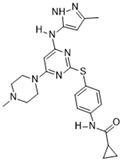	A: 0.6 nM B: 18 nM C: 4.6 nM	Phase I—Advanced solid malignancies [63]	NCT02532868
		Phase I and II—Advanced colorectal and solid malignancies	NCT00099346
		Phase I and II—Leukemia [64]	NCT00111683
		Phase II—Leukemia [65]	NCT00405054
		Phase II—Advanced non-small cell lung cancer	NCT00290550
		Phase I—Leukemia	NCT00500006
BI 811283 [66] 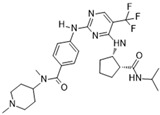	A: n/a B: 9 nM C: n/a	Phase I and II—Leukemia [67]	NCT00632749
		Phase I—Advanced solid malignancies [68]	NCT00701324
AT9283 [69] 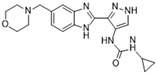	A: 3 nM B: 3 nM C: <10 nM	Phase II—Multiple myeloma [70]	NCT01145989
		Phase I—Advanced solid malignancies and non-Hodgkin’s lymphoma [71]	NCT00443976
		Phase I and II—Leukemia [72]	NCT00522990
		Phase I—Leukemia [73]	NCT01431664
		Phase I—Refractory solid malignancies [74]	NCT00985868
MLN8237 (Alisertib) [75] 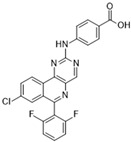	A: 1.2 nM B: 396.5 nM C: n/a	Phase I—Advanced solid malignancies [76]	NCT00249301
		Phase I—Advanced solid malignancies [77]	NCT00652158
ABT-348 (Ilorasertib) [78] 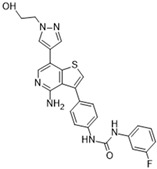	A: 120 nM B: 7 nM C: 1 nM	Phase I—Advanced solid malignancies [79]	NCT02540876
		Phase II—Advanced solid malignancies	NCT02478320
		Phase I—Advanced hematologic malignancies [80]	NCT01110473
		Phase I—Advanced solid malignancies [81]	NCT01110486
TAK-901 [82] 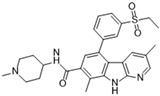	A: 21 nM B: 15 nM C: n/a	Phase I—Advanced hematologic malignancies	NCT00807677
		Phase I—Advanced solid malignancies and lymphoma	NCT00935844
CS2164 (Chiauranib) [83] 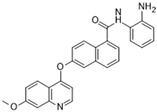	A: n/a B: 9 nM C: n/a	Phase I—Advanced solid malignancies [84]	NCT02122809
		Phase I and II—Small-cell lung cancer	NCT05271292
		Phase I and II—Hepatocellular carcinoma	NCT03245190
		Phase I and II—Ovarian cancer	NCT03166891
		Phase I—Non-Hodgkin’s lymphoma	NCT03074825
		Phase III—Ovarian cancer	NCT04921527
		Phase III—Small-cell lung cancer	NCT04830813
		Phase I and II—Non-Hodgkin’s lymphoma	NCT03974243
		Phase II—Ovarian cancer	NCT03901118
		Phase I—Small-cell lung cancer	NCT03216343

Note: A: Aurora kinase A; B: Aurora kinase B; C: Aurora kinase C; n/a: data not available.

**Table 2:** Comprehensive list of Aurora B inhibitors in preclinical development.

Compound Name and Structure	In Vitro IC_50_	Preclinical In Vivo and In Vitro Activity
SP-96 [32] 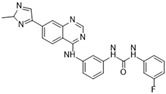	A: n/a B: 0.316 nM C: n/a	- >2000-fold selectivity for Aurora B over FLT3 and KIT; - Selective growth inhibition in NCI60 screening; - GI_50_ < 1000 nM for most cell lines; - First ever ATP-non-competitive inhibitor of Aurora B.
ZM447439 [111] 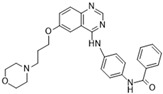	A: 110 nM B: 130 nM C: n/a	- Eight-fold selectivity for Aurora A and B over MEK1, SRS, and LCk; - IC_50_ > 10 nM for CDK1 and PLK1; - Induction of apoptosis through promotion of DNA fragmentation and caspase 3/7 activation; - Induces arrest of GEP-NET cells during the G0, G1, and G2/M phases of mitosis.
PHA-680632 [112] 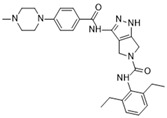	A: 27 nM B: 135 nM C: 120 nM	- Induces polyploidy in tumor cells; - From 10- to 200-fold greater selectivity for FGFR1, FLT3, LCK, PLK1, STLK2, VEGFR2, and VEGFR3; - Through reducing tumor cell proliferation and increasing cellular apoptosis, has been shown to inhibit tumor growth in murine xenografts for a variety of different cancer cells.
CCT129202 [113] 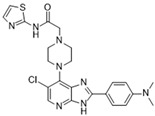	A: 42 nM B: 198 nM C: 227 nM	- Decreases histone H3 phosphorylation and increases p53 stabilization in HCT116 cells; - Induces p21 upregulation in a p53 dependent and independent manner in a variety of cell lines; - Causes the decreased phosphorylation of Rb and E2F activity in a concentration-dependent fashion; - Fifty percent reduction in histone H3 phosphorylation and tumor growth inhibition of 57.7% in murine mice with HCT116 xenografts.
CCT137690 [114] 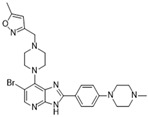	A: 15 nM B: 25 nM C: 19 nM	- Antiproliferative properties in multiple cancer cell lines; - Induction of apoptosis, polyploidy, and mitotic aberrations in cancer cells; - In vivo inhibition of tumor growth in MYCN-driven transgenic malignancies when analyzed in murine xenografts.
GSK650394 [115] 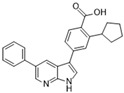	A: n/a B: 5.28 nM C: n/a	- Causes cell cycle arrest in the G2/M phase; - IC_50_ of 1290 nM for Aspergillus fumigatus; - Suppresses cancer cell and Aspergillus fumigatus proliferation.
Reversine [116] 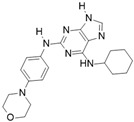	A: 400 nM B: 500 nM C: 400 nM	- Inhibits MEK1, NMMII, and MPS1 kinase; - MEK1 IC_50_ > 1500 nM and NMII IC_50_ = 350 nM; - Approximately 2-fold greater affinity for Mps1 than Aurora B; - Inhibits cell proliferation and causes induction of apoptosis by modulation of caspase 3 and Bax/Bcl-2; - IC_50_ of 660 nM for the human A3 adenosine receptor; - Inhibits phosphorylation of histone H3 in HCT116 cells; - Reduced tumor growth in cervical cancer cells inoculated in murine xenografts when combined with aspirin.
Hesperadin [117] 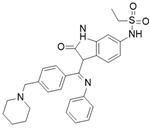	A: n/a B: 250 nM C: n/a	- Induction of polyploidy in HeLa cells through chromosome segregation and defected cytokines; - Reduces activity of AMPK, LCk, MKK1, MAPKAP-K1, CHK1, and PHK; - Reverse mitotic arrest caused by monastrol and taxol; - Inhibits cellular growth of cultured infected bloodstreams (IC_50_ = 48 nM).

Note: A: Aurora kinase A; B: Aurora kinase B; C: Aurora kinase C; n/a: data not available.

## Data Availability

The data for Figure 2 was retrieved from rcsb.org (accessed on 15 March 2023).

## Supplementary Materials

The following are available online at https://www.mdpi.com/article/10.3390/molecules28083385/s1, Table S1: Comprehensive list of IC_50_ data for Aurora B inhibitors reported in literature. Supplementary S1: Aurora B inhibitors.

## Sample Availability

Samples of the compounds are not available from the authors.
