# Supplementary material for: Aurora B Inhibitors as Cancer Therapeutics
Source: Molecules. 2023 Apr 11;28(8):3385. doi: 10.3390/molecules28083385 (PMC10144992; doi:10.3390/molecules28083385)
Supplement: Supplementary file 1 [file molecules-28-03385-s001.zip › molecules-2259316-Supplementary S1 Aurora B inhibitors.pdf]

Supplementary S1: Aurora B inhibitors.

```
BI 847325
  DSViewer          3D                              0

 35 38  0  0  0  0  0  0  0  0999 V2000
    7.7600    2.5630    5.0420 C   0  0
    9.1380    2.7820    5.3110 C   0  0
    9.5400    3.9010    6.0720 C   0  0
   11.3770    2.7170    5.7610 C   0  0
   11.7650    0.2720    4.4280 N   0  0
    9.3550    0.1710    3.6850 C   0  0
    7.6590   -1.5180    3.2220 C   0  0
    7.7970    0.4420    1.7880 C   0  0
   13.6390   -1.0830    4.1730 C   0  0
   14.3470   -2.1100    3.5970 C   0  0
   13.7880   -2.7810    2.5000 C   0  0
   11.7830   -1.4550    2.6250 C   0  0
    5.3670    6.3480    6.8130 C   0  0
    3.3730    7.2900    6.1710 O   0  0
    4.2740    7.2670    7.0070 C   0  0
    4.1840    8.0940    8.0480 N   0  0
    3.0030    8.9930    8.1430 C   0  0
    2.8410    9.5210    9.5500 C   0  0
    6.2060    5.5790    6.5230 C   0  0
    7.2190    4.6500    6.1690 C   0  0
    8.5810    4.8550    6.4670 C   0  0
   10.9040    3.8410    6.2860 N   0  0
   12.5490    2.4240    5.7540 O   0  0
   10.3490    1.9890    5.0910 C   0  0
    6.8280    3.5070    5.4080 C   0  0
   10.4970    0.7890    4.3820 C   0  0
    8.8650    0.8980    2.5300 C   0  0
    7.1730   -0.7770    2.1350 C   0  0
    8.7410   -1.0910    4.0170 C   0  0
   12.3560   -0.7830    3.7370 C   0  0
   12.5090   -2.4660    2.0250 C   0  0
   14.5910   -3.8710    1.8200 C   0  0
   14.5000   -5.1650    2.4980 N   0  0
   14.7850   -6.2310    1.5090 C   0  0
   15.4450   -5.2370    3.6450 C   0  0
  1  2  4  0  0  0
  1 25  4  0  0  0
  2  3  4  0  0  0
  2 24  1  0  0  0
  3 21  4  0  0  0
  3 22  1  0  0  0
  4 22  1  0  0  0
  4 23  2  0  0  0
  4 24  1  0  0  0
  5 26  1  0  0  0
  5 30  1  0  0  0
  6 26  1  0  0  0
```

```
  6 27  4  0  0  0
  6 29  4  0  0  0
  7 28  4  0  0  0
  7 29  4  0  0  0
  8 27  4  0  0  0
  8 28  4  0  0  0
  9 10  4  0  0  0
  9 30  4  0  0  0
 10 11  4  0  0  0
 11 31  4  0  0  0
 11 32  1  0  0  0
 12 30  4  0  0  0
 12 31  4  0  0  0
 13 15  1  0  0  0
 13 19  3  0  0  0
 14 15  2  0  0  0
 15 16  1  0  0  0
 16 17  1  0  0  0
 17 18  1  0  0  0
 19 20  1  0  0  0
 20 21  4  0  0  0
 20 25  4  0  0  0
 24 26  2  0  0  0
 32 33  1  0  0  0
 33 34  1  0  0  0
 33 35  1  0  0  0
M  END
> <Resolution>
1.93

$$$$
VX-680
  DSViewer          3D                              0

 33 37  0  0  0  0  0  0  0  0999 V2000
   44.0070   48.0820   54.1290 N   0  0
   42.6140   47.6750   54.4180 C   0  0
   42.3440   47.7770   55.9240 C   0  0
   42.6900   49.1460   56.3750 N   0  0
   44.0440   49.6690   56.0430 C   0  0
   44.2900   49.4770   54.5360 C   0  0
   44.2450   47.9480   52.6810 C   0  0
   41.7500   49.9340   57.0780 C   0  0
   40.6760   49.3350   57.7350 C   0  0
   39.7630   50.1360   58.4180 C   0  0
   39.9420   51.4660   58.4200 N   0  0
   40.9800   52.0360   57.7830 C   0  0
   41.8730   51.2770   57.1180 N   0  0
   38.7280   49.5620   59.0420 N   0  0
   37.9920   50.1040   60.0280 C   0  0
   37.9700   51.3660   60.4530 C   0  0
   37.0710   51.3470   61.4300 C   0  0
```

36.5820	50.1260	61.5650	N	0	0
37.1270	49.3950	60.7380	N	0	0
36.6350	52.5270	62.2930	C	0	0
41.1650	53.7850	57.8290	S	0	0
39.5770	54.2770	58.4090	C	0	0
39.3180	54.3800	59.7770	C	0	0
38.0530	54.7730	60.2220	C	0	0
37.0350	55.0600	59.3030	C	0	0
37.3090	54.9530	57.9380	C	0	0
38.5700	54.5650	57.4920	C	0	0
35.7920	55.4360	59.6580	N	0	0
35.3420	55.7770	60.8760	C	0	0
36.0160	55.8020	61.9100	O	0	0
33.8470	56.1530	60.8890	C	0	0
33.5260	57.5720	61.3710	C	0	0
33.2100	56.3700	62.2660	C	0	0

1	2	1	0	0	0
1	6	1	0	0	0
1	7	1	0	0	0
2	3	1	0	0	0
3	4	1	0	0	0
4	5	1	0	0	0
4	8	1	0	0	0
5	6	1	0	0	0
8	9	4	0	0	0
8	13	4	0	0	0
9	10	4	0	0	0
10	11	4	0	0	0
10	14	1	0	0	0
11	12	4	0	0	0
12	13	4	0	0	0
12	21	1	0	0	0
14	15	1	0	0	0
15	16	2	0	0	0
15	19	1	0	0	0
16	17	1	0	0	0
17	18	2	0	0	0
17	20	1	0	0	0
18	19	1	0	0	0
21	22	1	0	0	0
22	23	4	0	0	0
22	27	4	0	0	0
23	24	4	0	0	0
24	25	4	0	0	0
25	26	4	0	0	0
25	28	1	0	0	0
26	27	4	0	0	0
28	29	1	0	0	0
29	30	2	0	0	0
29	31	1	0	0	0
31	32	1	0	0	0
31	33	1	0	0	0

```
 32 33  1  0  0  0
M  END
> <Resolution>
1.85

$$$$
BI 811283
  DSViewer          3D                             0

 40 43  0  0  0  0  0  0  0  0999 V2000
    9.9170    2.3940    4.9620 C   0  0
   10.2020    3.6130    5.6530 C   0  0
   12.1550   -0.8520    3.4560 C   0  0
   14.6790   -1.9660    3.2050 C   0  0
   13.7860   -3.6890    1.5740 C   0  0
    8.6400    0.8170    2.3850 C   0  0
    7.3230    1.0650    1.6570 C   0  0
    6.2540    1.3030    2.7130 C   0  0
    6.1520    1.0040    5.2500 C   0  0
    5.7940    0.6880    8.4060 C   0  0
   11.5000    3.7400    6.1200 C   0  0
   12.4660    2.8370    5.8990 N   0  0
   12.1000    1.7460    5.2060 C   0  0
   10.8730    1.4790    4.7280 N   0  0
    8.6720    2.1000    4.5210 N   0  0
   13.0870    0.8260    4.9930 N   0  0
   13.2080   -0.3450    4.2120 C   0  0
   12.3520   -1.9440    2.6260 C   0  0
   13.6040   -2.5560    2.5380 C   0  0
   14.4850   -0.8730    4.0340 C   0  0
   13.4220   -3.5090    0.4220 O   0  0
   14.3410   -4.8610    1.9860 N   0  0
   14.8030   -5.1460    3.3440 C   0  0
    8.3060    0.8470    3.8850 C   0  0
    6.7840    0.5540    3.9430 C   0  0
    5.7280    0.0100    6.0490 N   0  0
    6.0530    2.1990    5.5290 O   0  0
    4.9380    0.1810    7.2790 C   0  0
    3.7100    1.0380    7.0570 C   0  0
    9.1770    4.6750    5.9470 C   0  0
    8.2400    4.2530    6.7800 F   0  0
    9.7070    5.7770    6.4670 F   0  0
    8.5200    5.0770    4.8350 F   0  0
   14.5820   -6.0480    1.0630 C   0  0
   15.5910   -5.7540   -0.0500 C   0  0
   15.8250   -6.9740   -0.9430 C   0  0
   14.5270   -7.4790   -1.4880 N   0  0
   13.6160   -7.8890   -0.3730 C   0  0
   13.3010   -6.7000    0.5370 C   0  0
   14.7350   -8.5880   -2.4630 C   0  0
  1  2  1  0  0  0
  1 14  1  0  0  0
```

```
  1 15  2  0  0  0
  2 11  2  0  0  0
  2 30  1  0  0  0
  3 17  4  0  0  0
  3 18  4  0  0  0
  4 19  4  0  0  0
  4 20  4  0  0  0
  5 19  1  0  0  0
  5 21  2  0  0  0
  5 22  1  0  0  0
  6  7  1  0  0  0
  6 24  1  0  0  0
  7  8  1  0  0  0
  8 25  1  0  0  0
  9 25  1  0  0  0
  9 26  1  0  0  0
  9 27  2  0  0  0
 10 28  1  0  0  0
 11 12  1  0  0  0
 12 13  2  0  0  0
 13 14  1  0  0  0
 13 16  1  0  0  0
 15 24  1  0  0  0
 16 17  1  0  0  0
 17 20  4  0  0  0
 18 19  4  0  0  0
 22 23  1  0  0  0
 22 34  1  0  0  0
 24 25  1  0  0  0
 26 28  1  0  0  0
 28 29  1  0  0  0
 30 31  1  0  0  0
 30 32  1  0  0  0
 30 33  1  0  0  0
 34 35  1  0  0  0
 34 39  1  0  0  0
 35 36  1  0  0  0
 36 37  1  0  0  0
 37 38  1  0  0  0
 37 40  1  0  0  0
 38 39  1  0  0  0
M  END
> <Resolution>
1.6

$$$$
ZM447439
  DSViewer          3D                             0

 38 42  0  0  0  0  0  0  0  0999 V2000
   44.1060   46.4760   54.4970 C   0  0
   44.1830   45.5890   53.2480 C   0  0
```

43.5600	46.2460	52.1140	O	0	0
43.8400	47.6670	52.0600	C	0	0
43.1810	48.3730	53.2490	C	0	0
43.0190	47.4780	54.4130	N	0	0
43.0290	48.3040	55.6310	C	0	0
42.2100	47.6790	56.7660	C	0	0
41.7650	48.7720	57.7350	C	0	0
40.7210	49.5350	57.1120	O	0	0
39.8490	50.1150	57.9890	C	0	0
39.0190	49.3740	58.8270	C	0	0
39.8000	51.5100	58.0010	C	0	0
40.6470	52.1500	57.1360	O	0	0
40.5400	53.5920	57.1540	C	0	0
38.9250	52.1760	58.8670	C	0	0
38.0950	51.4380	59.7150	C	0	0
38.1480	50.0350	59.6900	C	0	0
37.3610	49.3060	60.4970	N	0	0
36.4740	49.9410	61.3710	C	0	0
36.4200	51.3360	61.4050	N	0	0
37.2090	52.0680	60.5960	C	0	0
37.1620	53.4230	60.5990	N	0	0
36.0820	53.9830	61.1790	C	0	0
36.2320	54.7750	62.3140	C	0	0
35.1140	55.3470	62.9220	C	0	0
34.7980	53.7630	60.6670	C	0	0
33.6770	54.3240	61.2780	C	0	0
33.8380	55.1310	62.4000	C	0	0
32.7640	55.6510	63.0110	N	0	0
32.2890	56.8780	62.7310	C	0	0
32.7620	57.6650	61.9060	O	0	0
31.1710	57.1970	63.5010	C	0	0
29.9710	57.5870	62.9010	C	0	0
28.8620	57.8880	63.6960	C	0	0
28.9500	57.7930	65.0870	C	0	0
30.1440	57.4000	65.6910	C	0	0
31.2500	57.0980	64.8970	C	0	0

1	2	1	0	0	0
1	6	1	0	0	0
2	3	1	0	0	0
3	4	1	0	0	0
4	5	1	0	0	0
5	6	1	0	0	0
6	7	1	0	0	0
7	8	1	0	0	0
8	9	1	0	0	0
9	10	1	0	0	0
10	11	1	0	0	0
11	12	4	0	0	0
11	13	4	0	0	0
12	18	4	0	0	0
13	14	1	0	0	0
13	16	4	0	0	0

```
 14 15  1  0  0  0
 16 17  4  0  0  0
 17 18  4  0  0  0
 17 22  4  0  0  0
 18 19  4  0  0  0
 19 20  4  0  0  0
 20 21  4  0  0  0
 21 22  4  0  0  0
 22 23  1  0  0  0
 23 24  1  0  0  0
 24 25  4  0  0  0
 24 27  4  0  0  0
 25 26  4  0  0  0
 26 29  4  0  0  0
 27 28  4  0  0  0
 28 29  4  0  0  0
 29 30  1  0  0  0
 30 31  1  0  0  0
 31 32  2  0  0  0
 31 33  1  0  0  0
 33 34  4  0  0  0
 33 38  4  0  0  0
 34 35  4  0  0  0
 35 36  4  0  0  0
 36 37  4  0  0  0
 37 38  4  0  0  0
M  END
> <Resolution>
1.86

$$$$
Reversine
  DSViewer          3D                              0

 29 33  0  0  0  0  0  0  0  0999 V2000
    7.4330    0.4430    2.4390 C   0  0
    6.2010    1.2800    2.0640 C   0  0
    5.0300    1.0330    3.0310 C   0  0
    5.5070    0.4120    4.3540 C   0  0
    6.8000    1.0790    4.8350 C   0  0
    7.9550    0.8340    3.8350 C   0  0
    8.7770    2.0490    3.7300 N   0  0
    9.9290    2.1840    4.4000 C   0  0
   10.8870    1.2450    4.3620 N   0  0
   10.0780    3.2980    5.1130 C   0  0
    9.2900    4.3510    5.3070 N   0  0
    9.9270    5.1930    6.1150 C   0  0
   11.1070    4.6580    6.4190 N   0  0
   11.2040    3.4870    5.8020 C   0  0
   12.1770    2.5600    5.7730 N   0  0
   12.0290    1.4300    5.0480 C   0  0
   13.0270    0.5220    5.0650 N   0  0
```

```
   13.2180   -0.4310    4.1230 C    0   0
   14.5080   -0.9480    4.0140 C    0   0
   14.8090   -1.9400    3.0920 C    0   0
   12.2140   -0.9380    3.2900 C    0   0
   12.5110   -1.9290    2.3510 C    0   0
   13.8110   -2.4370    2.2540 C    0   0
   14.1310   -3.3890    1.3550 N    0   0
   13.4760   -4.7300    1.3930 C    0   0
   14.4290   -5.8380    0.9340 C    0   0
   15.1330   -5.4230   -0.2650 O    0   0
   16.0110   -4.3030   -0.0030 C    0   0
   15.1600   -3.0750    0.3180 C    0   0
  1  2  1  0  0  0
  1  6  1  0  0  0
  2  3  1  0  0  0
  3  4  1  0  0  0
  4  5  1  0  0  0
  5  6  1  0  0  0
  6  7  1  0  0  0
  7  8  1  0  0  0
  8  9  4  0  0  0
  8 10  4  0  0  0
  9 16  4  0  0  0
 10 11  1  0  0  0
 10 14  4  0  0  0
 11 12  2  0  0  0
 12 13  1  0  0  0
 13 14  1  0  0  0
 14 15  4  0  0  0
 15 16  4  0  0  0
 16 17  1  0  0  0
 17 18  1  0  0  0
 18 19  4  0  0  0
 18 21  4  0  0  0
 19 20  4  0  0  0
 20 23  4  0  0  0
 21 22  4  0  0  0
 22 23  4  0  0  0
 23 24  1  0  0  0
 24 25  1  0  0  0
 24 29  1  0  0  0
 25 26  1  0  0  0
 26 27  1  0  0  0
 27 28  1  0  0  0
 28 29  1  0  0  0
M  END
> <Resolution>
1.7

$$$$
Hesperadin
  DSViewer          3D                              0
```

```
 37 41  0  0  0  0  0  0  0  0999 V2000
   16.0320   -4.5090    3.5270 C   0  0
   16.3080   -5.8530    4.2050 C   0  0
   14.9940   -6.6020    4.4400 C   0  0
   14.2350   -6.7630    3.1220 C   0  0
   14.0080   -5.3920    2.4770 C   0  0
   15.2950   -4.7130    2.2670 N   0  0
   15.0870   -3.4400    1.5530 C   0  0
   14.2780   -2.4260    2.3650 C   0  0
   12.9520   -2.1540    2.0370 C   0  0
   12.2390   -1.1890    2.7430 C   0  0
   14.8830   -1.7340    3.4120 C   0  0
   14.1690   -0.7670    4.1120 C   0  0
   12.8500   -0.4810    3.7770 C   0  0
   12.2800    0.5550    4.3960 N   0  0
   11.0710    1.0900    4.2110 C   0  0
   10.0530    0.4720    3.4810 C   0  0
    9.5070    1.1020    2.3690 C   0  0
    8.4260    0.5380    1.6970 C   0  0
    7.8910   -0.6720    2.1370 C   0  0
    8.4400   -1.3100    3.2430 C   0  0
    9.5180   -0.7410    3.9160 C   0  0
   10.7730    2.2290    4.9410 C   0  0
    9.5280    2.8320    5.0960 C   0  0
    8.2590    2.3720    4.7850 C   0  0
   11.6770    3.0350    5.6310 C   0  0
   12.8610    2.7700    5.7690 O   0  0
   11.0210    4.0750    6.1580 N   0  0
    9.7240    3.9730    5.8700 C   0  0
    8.5920    4.6490    6.3300 C   0  0
    7.3130    4.1690    6.0510 C   0  0
    7.1380    3.0180    5.2830 C   0  0
    5.9660    2.5950    4.8120 N   0  0
    4.5050    3.0170    5.3880 S   0  0
    3.5080    1.9410    5.0770 O   0  0
    4.0680    4.3390    4.8300 O   0  0
    4.7680    3.1690    7.1920 C   0  0
    5.0360    1.7690    7.7530 C   0  0
  1  2  1  0  0  0
  1  6  1  0  0  0
  2  3  1  0  0  0
  3  4  1  0  0  0
  4  5  1  0  0  0
  5  6  1  0  0  0
  6  7  1  0  0  0
  7  8  1  0  0  0
  8  9  1  0  0  0
  8 11  1  0  0  0
  9 10  2  0  0  0
 10 13  1  0  0  0
 11 12  2  0  0  0
```

```
 12 13  1  0  0  0
 13 14  2  0  0  0
 14 15  1  0  0  0
 15 16  1  0  0  0
 15 22  1  0  0  0
 16 17  4  0  0  0
 16 21  4  0  0  0
 17 18  4  0  0  0
 18 19  4  0  0  0
 19 20  4  0  0  0
 20 21  4  0  0  0
 22 23  2  0  0  0
 22 25  1  0  0  0
 23 24  1  0  0  0
 23 28  1  0  0  0
 24 31  2  0  0  0
 25 26  2  0  0  0
 25 27  1  0  0  0
 27 28  2  0  0  0
 28 29  1  0  0  0
 29 30  2  0  0  0
 30 31  1  0  0  0
 31 32  1  0  0  0
 32 33  1  0  0  0
 33 34  2  0  0  0
 33 35  2  0  0  0
 33 36  1  0  0  0
 36 37  1  0  0  0
M  END
> <Resolution>
1.8

$$$$
Aminothiazole 25
  DSViewer          3D                             0

 17 18  0  0  0  0  0  0  0  0999 V2000
   12.3400    2.8260    6.2340 N   0  0
   11.3700    3.7560    6.3430 C   0  0
   10.2810    3.4710    5.6180 C   0  0
   10.4890    2.0760    4.8150 S   0  0
   12.0480    1.8060    5.4180 C   0  0
   12.9140    0.7800    5.2240 N   0  0
   12.8580   -0.1470    4.2400 C   0  0
   11.6990   -0.5010    3.5400 C   0  0
   11.7400   -1.4780    2.5370 C   0  0
   14.0540   -0.7990    3.9310 C   0  0
   14.0960   -1.7810    2.9350 C   0  0
   12.9410   -2.1260    2.2250 C   0  0
   12.9890   -3.1020    1.2160 C   0  0
   13.7630   -4.0620    1.2910 O   0  0
   12.1480   -2.8920    0.1780 N   0  0
```

```
   12.1300   -3.8630   -0.9340 C   0  0
    8.7360    4.5630    5.5650 Br  0  0
  1  2  4  0  0  0
  1  5  4  0  0  0
  2  3  4  0  0  0
  3  4  4  0  0  0
  3 17  1  0  0  0
  4  5  4  0  0  0
  5  6  1  0  0  0
  6  7  1  0  0  0
  7  8  4  0  0  0
  7 10  4  0  0  0
  8  9  4  0  0  0
  9 12  4  0  0  0
 10 11  4  0  0  0
 11 12  4  0  0  0
 12 13  1  0  0  0
 13 14  2  0  0  0
 13 15  1  0  0  0
 15 16  1  0  0  0
M  END
> <Resolution>
1.7

$$$$
GSK1070916
  DSViewer          3D                             0

 38 42  0  0  0  0  0  0  0  0999 V2000
   31.9187  -35.3207    0.0012 C   0  0
   31.4922  -36.5856    0.0037 C   0  0
   32.4586  -37.6489    0.0119 C   0  0
   33.7537  -37.3742    0.0174 C   0  0
   34.2585  -36.0224    0.0149 C   0  0
   33.3683  -35.0272    0.0069 C   0  0
   30.1265  -36.9455   -0.0002 N   0  0
   29.0482  -36.0451   -0.0016 C   0  0
   29.2168  -34.8209    0.0004 O   0  0
   27.7543  -36.5605   -0.0033 N   0  0
   26.6291  -35.6750   -0.0027 C   0  0
   27.5511  -37.9826   -0.0054 C   0  0
   35.7414  -35.6675    0.0212 C   0  0
   37.0369  -36.5192    0.0279 C   0  0
   38.0245  -35.6306    0.0300 C   0  0
   37.4259  -34.3651    0.0259 N   0  0
   36.0876  -34.4420    0.0207 N   0  0
   38.1522  -33.1207    0.0173 C   0  0
   39.6718  -33.3850    0.0055 C   0  0
   37.2731  -38.0673    0.0225 C   0  0
   36.1603  -39.0134    0.0074 C   0  0
   36.3372  -40.3355   -0.0086 C   0  0
   37.6138  -40.8668   -0.0150 N   0  0
```

38.5944	-40.0704	0.0009	C	0	0
38.4797	-38.6632	0.0232	C	0	0
39.9017	-40.4205	-0.0127	N	0	0
40.6428	-39.2225	0.0052	C	0	0
39.8572	-38.1293	0.0314	C	0	0
42.0925	-39.2564	-0.0333	C	0	0
42.6671	-40.4518	-0.1356	C	0	0
44.1055	-40.5732	-0.2227	C	0	0
44.8593	-39.4812	-0.2039	C	0	0
44.2458	-38.1440	-0.0720	C	0	0
42.9144	-38.0401	0.0023	C	0	0
45.0965	-36.8703	-0.0685	C	0	0
46.5781	-37.0928	-0.1715	N	0	0
47.2230	-35.7974	-0.4195	C	0	0
47.1588	-37.6435	1.0822	C	0	0

1	2	2	0	0	0
2	3	1	0	0	0
3	4	2	0	0	0
4	5	1	0	0	0
5	6	2	0	0	0
6	1	1	0	0	0
2	7	1	0	0	0
7	8	1	0	0	0
8	9	2	0	0	0
8	10	1	0	0	0
10	11	1	0	0	0
10	12	1	0	0	0
5	13	1	0	0	0
13	14	1	0	0	0
14	15	2	0	0	0
15	16	1	0	0	0
16	17	1	0	0	0
17	13	2	0	0	0
16	18	1	0	0	0
18	19	1	0	0	0
14	20	1	0	0	0
20	21	1	0	0	0
21	22	2	0	0	0
22	23	1	0	0	0
23	24	2	0	0	0
24	25	1	0	0	0
25	20	2	0	0	0
24	26	1	0	0	0
26	27	1	0	0	0
27	28	2	0	0	0
28	25	1	0	0	0
27	29	1	0	0	0
29	30	2	0	0	0
30	31	1	0	0	0
31	32	2	0	0	0
32	33	1	0	0	0
33	34	2	0	0	0

```
 34 29  1  0  0  0
 33 35  1  0  0  0
 35 36  1  0  0  0
 36 37  1  0  0  0
 36 38  1  0  0  0
M  END
> <Clean Energy>
54.7492

$$$$
CYC116
  DSViewer          3D                             0

 26 29  0  0  0  0  0  0  0  0999 V2000
    2.3522  -27.6909    0.3141 C   0  0
    2.3454  -28.9873   -0.2801 O   0  0
    3.4178  -29.7176    0.3064 C   0  0
    4.7686  -29.1374   -0.1505 C   0  0
    4.8084  -27.6877   -0.0489 N   0  0
    3.5951  -26.8959   -0.1388 C   0  0
    6.0552  -27.0494   -0.0278 C   0  0
    7.2673  -27.8557   -0.0233 C   0  0
    8.4585  -27.2698   -0.0083 C   0  0
    8.5775  -25.8052    0.0043 C   0  0
    7.4659  -25.0681    0.0020 C   0  0
    6.1446  -25.7186   -0.0148 C   0  0
    9.8514  -25.1506    0.0156 N   0  0
   11.1086  -25.8306    0.0116 C   0  0
   11.1678  -27.1038    0.0074 N   0  0
   12.3815  -27.7787    0.0024 C   0  0
   13.5099  -27.0864    0.0012 C   0  0
   13.4410  -25.6395    0.0055 C   0  0
   12.3005  -25.0704    0.0106 N   0  0
   14.7149  -24.8840    0.0033 C   0  0
   16.2534  -25.6810   -0.0025 S   0  0
   17.0752  -24.1442   -0.0021 C   0  0
   16.2684  -23.1518    0.0019 N   0  0
   14.8848  -23.5519    0.0050 C   0  0
   13.7717  -22.5368    0.0097 C   0  0
   18.4647  -24.0132   -0.0056 N   0  0
  1  2  1  0  0  0
  2  3  1  0  0  0
  3  4  1  0  0  0
  4  5  1  0  0  0
  5  6  1  0  0  0
  6  1  1  0  0  0
  5  7  1  0  0  0
  7  8  1  0  0  0
  8  9  2  0  0  0
  9 10  1  0  0  0
 10 11  2  0  0  0
 11 12  1  0  0  0
```

```
 12  7  2  0  0  0
 10 13  1  0  0  0
 13 14  1  0  0  0
 14 15  2  0  0  0
 15 16  1  0  0  0
 16 17  2  0  0  0
 17 18  1  0  0  0
 18 19  2  0  0  0
 19 14  1  0  0  0
 18 20  1  0  0  0
 20 21  1  0  0  0
 21 22  1  0  0  0
 22 23  2  0  0  0
 23 24  1  0  0  0
 24 20  2  0  0  0
 24 25  1  0  0  0
 22 26  1  0  0  0
M  END
> <Clean Energy>
18.7169

$$$$
SNS-314
  DSViewer          3D                             0

 28 31  0  0  0  0  0  0  0  0999 V2000
    3.2693   -5.6983    0.0027 C   0  0
    3.1922   -7.0398    0.0015 C   0  0
    4.5383   -7.6648   -0.0001 C   0  0
    5.5287   -6.8088    0.0000 C   0  0
    4.9616   -5.1706    0.0021 S   0  0
    4.7786   -9.0302   -0.0018 N   0  0
    5.9889   -9.4359   -0.0033 C   0  0
    7.0791   -8.5179   -0.0033 N   0  0
    6.8988   -7.2470   -0.0016 C   0  0
    7.9783   -6.2706   -0.0013 N   0  0
    9.4455   -6.5409   -0.0026 C   0  0
    9.7812   -8.0365    0.0055 C   0  0
   11.2294   -8.3740    0.0032 C   0  0
   11.7589   -9.9976    0.0047 S   0  0
   13.4144   -9.4928    0.0004 C   0  0
   13.5350   -8.2154   -0.0019 N   0  0
   12.2547   -7.5185   -0.0004 C   0  0
   14.4939  -10.3957   -0.0005 N   0  0
   14.2624  -11.7959   -0.0004 C   0  0
   15.3082  -12.7455   -0.0007 N   0  0
   13.1054  -12.2264   -0.0002 O   0  0
   16.6874  -12.4556   -0.0006 C   0  0
   17.5411  -13.4771   -0.0009 C   0  0
   18.9806  -13.2386   -0.0007 C   0  0
   19.4494  -11.9925   -0.0002 C   0  0
   18.5187  -10.8621    0.0001 C   0  0
```

```
   17.2054  -11.0813   -0.0001 C   0  0
   20.0730  -14.5766   -0.0011 Cl  0  0
  1  2  2  0  0  0
  2  3  1  0  0  0
  3  4  2  0  0  0
  4  5  1  0  0  0
  5  1  1  0  0  0
  3  6  1  0  0  0
  6  7  2  0  0  0
  7  8  1  0  0  0
  8  9  2  0  0  0
  9  4  1  0  0  0
  9 10  1  0  0  0
 10 11  1  0  0  0
 11 12  1  0  0  0
 12 13  1  0  0  0
 13 14  1  0  0  0
 14 15  1  0  0  0
 15 16  2  0  0  0
 16 17  1  0  0  0
 17 13  2  0  0  0
 15 18  1  0  0  0
 18 19  1  0  0  0
 19 20  1  0  0  0
 19 21  2  0  0  0
 20 22  1  0  0  0
 22 23  2  0  0  0
 23 24  1  0  0  0
 24 25  2  0  0  0
 25 26  1  0  0  0
 26 27  2  0  0  0
 27 22  1  0  0  0
 24 28  1  0  0  0
M  END
> <Clean Energy>
32.9355

$$$$
AMG 900
  DSViewer          3D                             0

 37 42  0  0  0  0  0  0  0  0999 V2000
   31.7125   -5.7697   -0.0010 C   0  0
   31.7431   -7.0956   -0.0008 C   0  0
   33.0217   -7.7824   -0.0001 C   0  0
   34.1495   -7.0468    0.0002 C   0  0
   34.0759   -5.6437   -0.0002 N   0  0
   32.9634   -5.0290   -0.0008 C   0  0
   32.9949   -9.2961    0.0002 C   0  0
   31.8624   -9.8755   -0.0001 N   0  0
   31.7550  -11.2812    0.0000 C   0  0
   32.7933  -12.0196    0.0004 N   0  0
```

34.0700	-11.4556	0.0007	C	0	0
34.2016	-10.1334	0.0006	C	0	0
30.4910	-11.8726	-0.0002	N	0	0
35.3620	-7.6975	0.0011	O	0	0
36.5933	-7.0128	0.0009	C	0	0
37.6303	-7.8105	0.0026	C	0	0
38.9247	-7.2970	0.0024	C	0	0
39.2305	-6.0276	0.0003	C	0	0
38.0951	-5.0622	-0.0015	C	0	0
36.8313	-5.5315	-0.0011	C	0	0
40.6418	-5.5964	-0.0004	N	0	0
41.9693	-6.3815	-0.0002	C	0	0
42.2245	-7.9269	-0.0005	C	0	0
43.4847	-8.3570	-0.0001	C	0	0
44.5763	-7.3853	0.0003	C	0	0
44.3007	-6.1407	0.0004	N	0	0
43.0181	-5.6486	0.0002	N	0	0
41.1957	-8.9664	-0.0013	C	0	0
41.4479	-10.2648	-0.0013	C	0	0
42.7950	-10.7054	-0.0007	C	0	0
43.7583	-9.7997	-0.0002	C	0	0
46.0257	-7.8315	0.0004	C	0	0
46.6092	-9.4998	0.0005	S	0	0
48.2791	-9.0493	0.0004	C	0	0
48.3713	-7.7189	0.0003	C	0	0
47.0562	-6.9838	0.0004	C	0	0
49.6933	-7.0188	0.0002	C	0	0

1	2	2	0	0	0
2	3	1	0	0	0
3	4	2	0	0	0
4	5	1	0	0	0
5	6	2	0	0	0
6	1	1	0	0	0
3	7	1	0	0	0
7	8	2	0	0	0
8	9	1	0	0	0
9	10	2	0	0	0
10	11	1	0	0	0
11	12	2	0	0	0
12	7	1	0	0	0
9	13	1	0	0	0
4	14	1	0	0	0
14	15	1	0	0	0
15	16	2	0	0	0
16	17	1	0	0	0
17	18	2	0	0	0
18	19	1	0	0	0
19	20	2	0	0	0
20	15	1	0	0	0
18	21	1	0	0	0
21	22	1	0	0	0
22	23	1	0	0	0

```
 23 24  2  0  0  0
 24 25  1  0  0  0
 25 26  2  0  0  0
 26 27  1  0  0  0
 27 22  2  0  0  0
 23 28  1  0  0  0
 28 29  2  0  0  0
 29 30  1  0  0  0
 30 31  2  0  0  0
 31 24  1  0  0  0
 25 32  1  0  0  0
 32 33  1  0  0  0
 33 34  1  0  0  0
 34 35  2  0  0  0
 35 36  1  0  0  0
 36 32  2  0  0  0
 35 37  1  0  0  0
M  END
> <Clean Energy>
40.1834

$$$$
PHA-739358
  DSViewer          3D                              0

 35 39  0  0  0  0  0  0  0  0999 V2000
   28.0449  -24.5395    0.2897 C   0  0
   28.0226  -25.8802   -0.3006 N   0  0
   29.1386  -26.6102    0.2952 C   0  0
   30.4785  -26.0095   -0.1615 C   0  0
   30.5139  -24.5593   -0.0632 N   0  0
   29.3012  -23.7641   -0.1629 C   0  0
   31.7639  -23.9218   -0.0510 C   0  0
   32.9779  -24.7364   -0.0414 C   0  0
   34.1720  -24.1554   -0.0365 C   0  0
   34.2846  -22.7031   -0.0400 C   0  0
   33.1831  -21.9463   -0.0469 C   0  0
   31.8582  -22.5907   -0.0539 C   0  0
   35.6096  -22.0990   -0.0262 C   0  0
   36.5814  -22.8579   -0.0067 O   0  0
   35.7494  -20.7685   -0.0182 N   0  0
   36.9853  -20.0124    0.0181 C   0  0
   38.3829  -20.4567    0.0220 C   0  0
   39.1221  -19.4116    0.0958 C   0  0
   38.3423  -18.2751    0.1308 N   0  0
   37.0119  -18.7263    0.0806 N   0  0
   39.1336  -21.7292   -0.0287 C   0  0
   40.5088  -21.1589    0.0393 N   0  0
   40.5698  -19.6609    0.1308 C   0  0
   41.6441  -22.0179    0.0225 C   0  0
   41.4139  -23.2288   -0.0556 O   0  0
   43.1045  -21.4614    0.1442 C   0  0
```

```
   44.2583  -22.5716    0.0628 C   0  0
   43.9523  -23.8699   -0.0048 C   0  0
   44.9911  -24.9010   -0.0696 C   0  0
   46.2754  -24.5591   -0.0606 C   0  0
   46.6452  -23.1538    0.0230 C   0  0
   45.7013  -22.2168    0.0875 C   0  0
   43.2450  -20.3504   -0.8198 O   0  0
   44.3625  -19.4634   -0.5904 C   0  0
   26.7717  -26.5455    0.0861 C   0  0
  1  2  1  0  0  0
  2  3  1  0  0  0
  3  4  1  0  0  0
  4  5  1  0  0  0
  5  6  1  0  0  0
  6  1  1  0  0  0
  5  7  1  0  0  0
  7  8  1  0  0  0
  8  9  2  0  0  0
  9 10  1  0  0  0
 10 11  2  0  0  0
 11 12  1  0  0  0
 12  7  2  0  0  0
 10 13  1  0  0  0
 13 14  2  0  0  0
 13 15  1  0  0  0
 15 16  1  0  0  0
 16 17  1  0  0  0
 17 18  2  0  0  0
 18 19  1  0  0  0
 19 20  1  0  0  0
 20 16  2  0  0  0
 17 21  1  0  0  0
 21 22  1  0  0  0
 22 23  1  0  0  0
 23 18  1  0  0  0
 22 24  1  0  0  0
 24 25  2  0  0  0
 24 26  1  0  0  0
 26 27  1  0  0  0
 27 28  2  0  0  0
 28 29  1  0  0  0
 29 30  2  0  0  0
 30 31  1  0  0  0
 31 32  2  0  0  0
 32 27  1  0  0  0
 26 33  1  0  0  0
 33 34  1  0  0  0
  2 35  1  0  0  0
M  END
> <Clean Energy>
76.9805
```

```
$$$$
AT9283
  DSViewer          3D                             0

 28 32  0  0  0  0  0  0  0  0999 V2000
    2.4406  -37.3959    0.7261 C   0  0
    2.0944  -38.3771   -0.2657 O   0  0
    3.1398  -39.3549   -0.3946 C   0  0
    4.4474  -38.6567   -0.8294 C   0  0
    4.8394  -37.6729    0.2176 N   0  0
    3.7518  -36.6812    0.3277 C   0  0
    6.1117  -36.9412   -0.1024 C   0  0
    7.4200  -37.7579   -0.0348 C   0  0
    7.4406  -39.0823    0.2066 C   0  0
    8.7228  -39.8182    0.2744 C   0  0
    9.8324  -39.1114    0.1111 C   0  0
    9.8259  -37.7472   -0.1108 C   0  0
    8.7081  -37.0377   -0.1987 C   0  0
   11.1365  -39.5358    0.1318 N   0  0
   11.8910  -38.3345   -0.0644 C   0  0
   11.1378  -37.3023   -0.2056 N   0  0
   13.3523  -38.2703   -0.0844 C   0  0
   14.2551  -39.3846    0.0148 C   0  0
   15.4865  -38.9020   -0.0295 C   0  0
   15.3449  -37.5033   -0.1550 N   0  0
   14.0204  -37.1914   -0.1850 N   0  0
   13.8734  -40.7464    0.1271 N   0  0
   14.8000  -41.8188    0.2629 C   0  0
   14.3693  -42.9738    0.3572 O   0  0
   16.1845  -41.5985    0.3366 N   0  0
   17.0864  -42.6981    0.5502 C   0  0
   18.3964  -42.4376   -0.2141 C   0  0
   17.3591  -43.4350   -0.7714 C   0  0
  1  2  1  0  0  0
  2  3  1  0  0  0
  3  4  1  0  0  0
  4  5  1  0  0  0
  5  6  1  0  0  0
  6  1  1  0  0  0
  5  7  1  0  0  0
  7  8  1  0  0  0
  8  9  2  0  0  0
  9 10  1  0  0  0
 10 11  2  0  0  0
 11 12  1  0  0  0
 12 13  2  0  0  0
 13  8  1  0  0  0
 11 14  1  0  0  0
 14 15  1  0  0  0
 15 16  2  0  0  0
 16 12  1  0  0  0
 15 17  1  0  0  0
```

```
 17 18  1  0  0  0
 18 19  2  0  0  0
 19 20  1  0  0  0
 20 21  1  0  0  0
 21 17  2  0  0  0
 18 22  1  0  0  0
 22 23  1  0  0  0
 23 24  2  0  0  0
 23 25  1  0  0  0
 25 26  1  0  0  0
 26 27  1  0  0  0
 26 28  1  0  0  0
 27 28  1  0  0  0
M  END
> <Clean Energy>
67.3204

$$$$
TAK-901
  DSViewer          3D                              0

 36 40  0  0  0  0  0  0  0  0999 V2000
    2.3093  -12.2907    0.2880 C    0  0
    1.8876  -13.4951   -0.4710 N    0  0
    2.7603  -14.6391   -0.1591 C    0  0
    4.2135  -14.3093   -0.5450 C    0  0
    4.6943  -13.0782    0.2770 C    0  0
    3.7727  -11.8944   -0.0585 C    0  0
    0.5037  -13.8414   -0.1069 C    0  0
    6.1290  -12.7365    0.0707 N    0  0
    7.0804  -13.6821    0.1738 C    0  0
    6.7217  -14.8322    0.4445 O    0  0
    8.5737  -13.3555    0.0506 C    0  0
    9.5089  -14.3330    0.0660 C    0  0
   10.8808  -13.9899    0.0134 C    0  0
   11.3350  -12.7514   -0.0367 C    0  0
   10.3490  -11.5774   -0.0601 C    0  0
    9.0379  -11.9009   -0.0359 C    0  0
   11.9286  -14.9279    0.0273 N    0  0
   13.1034  -14.1950   -0.0224 C    0  0
   12.8406  -12.9058   -0.0666 C    0  0
   10.7339  -10.1078   -0.0628 C    0  0
   11.9817   -9.7336    0.1576 C    0  0
   12.3926   -8.3561    0.2041 C    0  0
   11.4936   -7.3949    0.0141 C    0  0
   10.0871   -7.7700   -0.2385 C    0  0
    9.7226   -9.0581   -0.2633 C    0  0
   14.0952   -7.9977    0.5653 S    0  0
   14.6712   -9.1291    1.3968 O    0  0
   14.2144   -6.6804    1.3062 O    0  0
   15.0353   -7.8925   -0.9757 C    0  0
   16.5179   -7.6219   -0.6476 C    0  0
```

```
   14.3946  -14.7105   -0.0259 N   0  0
   15.3873  -13.9101   -0.0725 C   0  0
   15.1743  -12.4454   -0.1280 C   0  0
   13.9342  -11.9550   -0.1286 C   0  0
   16.3536  -11.5241   -0.1826 C   0  0
    9.2061  -15.8112    0.1486 C   0  0
  1  2  1  0  0  0
  2  3  1  0  0  0
  3  4  1  0  0  0
  4  5  1  0  0  0
  5  6  1  0  0  0
  6  1  1  0  0  0
  2  7  1  0  0  0
  5  8  1  0  0  0
  8  9  1  0  0  0
  9 10  2  0  0  0
  9 11  1  0  0  0
 11 12  2  0  0  0
 12 13  1  0  0  0
 13 14  2  0  0  0
 14 15  1  0  0  0
 15 16  2  0  0  0
 16 11  1  0  0  0
 13 17  1  0  0  0
 17 18  1  0  0  0
 18 19  2  0  0  0
 19 14  1  0  0  0
 15 20  1  0  0  0
 20 21  2  0  0  0
 21 22  1  0  0  0
 22 23  2  0  0  0
 23 24  1  0  0  0
 24 25  2  0  0  0
 25 20  1  0  0  0
 22 26  1  0  0  0
 26 27  2  0  0  0
 26 28  2  0  0  0
 26 29  1  0  0  0
 29 30  1  0  0  0
 18 31  1  0  0  0
 31 32  2  0  0  0
 32 33  1  0  0  0
 33 34  2  0  0  0
 34 19  1  0  0  0
 33 35  1  0  0  0
 12 36  1  0  0  0
M  END
> <Clean Energy>
42.9504

$$$$
CS2164
```

```
 DSViewer          3D                              0

33 37  0  0  0  0  0  0  0  0999 V2000
   27.7371  -15.5385    0.0001 C   0  0
   27.6695  -17.0100    0.0002 C   0  0
   28.7893  -17.7215    0.0002 C   0  0
   30.0750  -17.0564    0.0002 C   0  0
   30.1426  -15.7198    0.0001 C   0  0
   28.9195  -14.9263    0.0001 C   0  0
   31.2286  -17.8416    0.0002 N   0  0
   32.3752  -17.3100    0.0002 C   0  0
   32.5027  -15.8730    0.0001 C   0  0
   31.4371  -15.0588    0.0001 C   0  0
   26.4430  -17.6787    0.0002 O   0  0
   25.3547  -16.7523    0.0002 C   0  0
   31.4740  -13.5426    0.0000 O   0  0
   32.7475  -12.7911   -0.0001 C   0  0
   33.9069  -13.4129   -0.0005 C   0  0
   35.1338  -12.7864   -0.0006 C   0  0
   35.2342  -11.4638   -0.0003 C   0  0
   33.9660  -10.6680    0.0001 C   0  0
   32.7800  -11.3052    0.0002 C   0  0
   36.2786  -13.6519   -0.0011 C   0  0
   37.4839  -13.1357   -0.0013 C   0  0
   37.6304  -11.7166   -0.0009 C   0  0
   36.6185  -10.8596   -0.0004 C   0  0
   36.8699   -9.3367    0.0001 C   0  0
   35.8636   -8.6157    0.0001 O   0  0
   38.0724   -8.6380    0.0005 N   0  0
   39.5354   -8.9709    0.0008 C   0  0
   40.0537  -10.1967    0.0017 C   0  0
   41.4844  -10.4488    0.0019 C   0  0
   42.3277   -9.4270    0.0012 C   0  0
   41.8002   -8.0671    0.0003 C   0  0
   40.4832   -7.8480    0.0001 C   0  0
   40.0054   -6.5420   -0.0008 N   0  0
  1  2  1  0  0  0
  2  3  2  0  0  0
  3  4  1  0  0  0
  4  5  2  0  0  0
  5  6  1  0  0  0
  6  1  2  0  0  0
  4  7  1  0  0  0
  7  8  2  0  0  0
  8  9  1  0  0  0
  9 10  2  0  0  0
 10  5  1  0  0  0
  2 11  1  0  0  0
 11 12  1  0  0  0
 10 13  1  0  0  0
 13 14  1  0  0  0
 14 15  2  0  0  0
```

```
 15 16  1  0  0  0
 16 17  2  0  0  0
 17 18  1  0  0  0
 18 19  2  0  0  0
 19 14  1  0  0  0
 16 20  1  0  0  0
 20 21  2  0  0  0
 21 22  1  0  0  0
 22 23  2  0  0  0
 23 17  1  0  0  0
 23 24  1  0  0  0
 24 25  2  0  0  0
 24 26  1  0  0  0
 26 27  1  0  0  0
 27 28  2  0  0  0
 28 29  1  0  0  0
 29 30  2  0  0  0
 30 31  1  0  0  0
 31 32  2  0  0  0
 32 27  1  0  0  0
 32 33  1  0  0  0
M  END
> <Clean Energy>
30.5624

$$$$
SP-96
  DSViewer          3D                              0

 34 38  0  0  0  0  0  0  0  0999 V2000
   43.9350  -31.2017   -0.0030 C   0  0
   44.0318  -32.5336   -0.0019 C   0  0
   45.3413  -33.1893    0.0005 C   0  0
   46.4521  -32.4564    0.0016 C   0  0
   46.3594  -30.9954    0.0005 C   0  0
   45.1664  -30.3995   -0.0017 C   0  0
   42.6630  -30.5299   -0.0059 N   0  0
   45.4204  -34.5288    0.0013 F   0  0
   41.3991  -31.2005   -0.0038 C   0  0
   41.3521  -32.4347    0.0020 O   0  0
   40.1863  -30.4226   -0.0078 N   0  0
   38.8672  -31.0007   -0.0040 C   0  0
   37.7852  -30.2098   -0.0096 C   0  0
   36.4184  -30.8117   -0.0036 C   0  0
   36.2931  -32.1368    0.0081 C   0  0
   37.4668  -32.9935    0.0128 C   0  0
   38.6822  -32.4587    0.0073 C   0  0
   35.2123  -30.0426   -0.0047 N   0  0
   35.1192  -28.6313   -0.0383 C   0  0
   36.1492  -27.8859   -0.1000 N   0  0
   36.0483  -26.4659   -0.1337 C   0  0
   34.9251  -25.8580   -0.1023 N   0  0
```

```
   33.7379  -26.5736   -0.0319 C   0  0
   33.8025  -28.0084    0.0003 C   0  0
   32.5625  -25.9472    0.0080 C   0  0
   31.3233  -26.7251    0.0873 C   0  0
   31.3792  -28.0573    0.1150 C   0  0
   32.6870  -28.7343    0.0678 C   0  0
   30.0319  -26.0319    0.1501 C   0  0
   29.8793  -24.6072    0.1188 C   0  0
   28.6361  -24.3546    0.1802 N   0  0
   27.8633  -25.5906    0.3036 C   0  0
   28.9066  -26.6041    0.2297 N   0  0
   26.8444  -25.7348   -0.8410 C   0  0
  1  2  2  0  0  0
  2  3  1  0  0  0
  3  4  2  0  0  0
  4  5  1  0  0  0
  5  6  2  0  0  0
  6  1  1  0  0  0
  1  7  1  0  0  0
  3  8  1  0  0  0
  7  9  1  0  0  0
  9 10  2  0  0  0
  9 11  1  0  0  0
 11 12  1  0  0  0
 12 13  2  0  0  0
 13 14  1  0  0  0
 14 15  2  0  0  0
 15 16  1  0  0  0
 16 17  2  0  0  0
 17 12  1  0  0  0
 14 18  1  0  0  0
 18 19  1  0  0  0
 19 20  2  0  0  0
 20 21  1  0  0  0
 21 22  2  0  0  0
 22 23  1  0  0  0
 23 24  1  0  0  0
 24 19  1  0  0  0
 23 25  2  0  0  0
 25 26  1  0  0  0
 26 27  2  0  0  0
 27 28  1  0  0  0
 28 24  2  0  0  0
 26 29  1  0  0  0
 29 30  1  0  0  0
 30 31  2  0  0  0
 31 32  1  0  0  0
 32 33  1  0  0  0
 33 29  2  0  0  0
 32 34  1  0  0  0
M  END
> <Clean Energy>
```

```
16.5824

$$$$
PHA-680632
  DSViewer          3D                              0

 37 41  0  0  0  0  0  0  0  0999 V2000
    3.1358  -42.6134    0.2764 C    0  0
    4.5943  -42.8263   -0.1717 C    0  0
    5.3985  -41.6226   -0.0522 N    0  0
    4.8014  -40.3004   -0.1465 C    0  0
    3.3275  -40.2787    0.2995 C    0  0
    2.5913  -41.3872   -0.3073 N    0  0
    1.1777  -41.2748    0.0740 C    0  0
    6.7950  -41.7338   -0.0255 C    0  0
    7.3771  -42.9343   -0.0285 C    0  0
    8.8401  -43.0445   -0.0107 C    0  0
    9.5827  -41.9396    0.0086 C    0  0
    8.9433  -40.6239    0.0152 C    0  0
    7.6179  -40.5245   -0.0010 C    0  0
   11.0332  -42.0162    0.0176 C    0  0
   11.6757  -40.9593    0.0311 O    0  0
   11.6727  -43.1916    0.0061 N    0  0
   13.0734  -43.1847    0.0071 C    0  0
   13.9713  -44.3069    0.0004 C    0  0
   15.1597  -43.8384    0.0003 C    0  0
   15.1428  -42.4642    0.0072 N    0  0
   13.7966  -42.1157    0.0109 N    0  0
   13.8717  -45.8001   -0.0061 C    0  0
   15.3709  -46.0350   -0.0086 N    0  0
   16.2194  -44.8133   -0.0067 C    0  0
   16.0200  -47.2746   -0.0104 C    0  0
   17.2407  -47.2069   -0.0121 O    0  0
   15.3240  -48.5388   -0.0055 N    0  0
   15.8613  -49.9419   -0.0004 C    0  0
   14.9264  -50.9009    0.0015 C    0  0
   15.2877  -52.3244    0.0069 C    0  0
   16.5555  -52.7103    0.0103 C    0  0
   17.6112  -51.7263    0.0086 C    0  0
   17.3515  -50.4187    0.0036 C    0  0
   18.5622  -49.5151    0.0029 C    0  0
   19.8781  -50.3202    0.0076 C    0  0
   13.4710  -50.5223   -0.0018 C    0  0
   12.5969  -51.7976    0.0016 C    0  0
  1  2  1  0  0  0
  2  3  1  0  0  0
  3  4  1  0  0  0
  4  5  1  0  0  0
  5  6  1  0  0  0
  6  1  1  0  0  0
  6  7  1  0  0  0
  3  8  1  0  0  0
```

```
  8  9  2  0  0  0
  9 10  1  0  0  0
 10 11  2  0  0  0
 11 12  1  0  0  0
 12 13  2  0  0  0
 13  8  1  0  0  0
 11 14  1  0  0  0
 14 15  2  0  0  0
 14 16  1  0  0  0
 16 17  1  0  0  0
 17 18  1  0  0  0
 18 19  2  0  0  0
 19 20  1  0  0  0
 20 21  1  0  0  0
 21 17  2  0  0  0
 18 22  1  0  0  0
 22 23  1  0  0  0
 23 24  1  0  0  0
 24 19  1  0  0  0
 23 25  1  0  0  0
 25 26  2  0  0  0
 25 27  1  0  0  0
 27 28  1  0  0  0
 28 29  2  0  0  0
 29 30  1  0  0  0
 30 31  2  0  0  0
 31 32  1  0  0  0
 32 33  2  0  0  0
 33 28  1  0  0  0
 33 34  1  0  0  0
 34 35  1  0  0  0
 29 36  1  0  0  0
 36 37  1  0  0  0
M  END
> <Clean Energy>
51.1074

$$$$
CCT129202
  DSViewer          3D                             0

 34 38  0  0  0  0  0  0  0  0999 V2000
    7.0406  -32.3070    0.0350 C   0  0
    7.2146  -33.6329    0.0630 C   0  0
    8.4835  -34.2041    0.0527 N   0  0
    9.4975  -33.4511    0.0089 C   0  0
    9.4522  -32.0504   -0.0296 C   0  0
    8.2830  -31.3837   -0.0187 C   0  0
   10.7881  -33.8446    0.0023 N   0  0
   11.5444  -32.6535   -0.0334 C   0  0
   10.8029  -31.5951   -0.0561 N   0  0
   13.0107  -32.6926   -0.0326 C   0  0
```

8.1946	-29.8974	-0.0473	N	0	0
5.3863	-31.7622	0.0368	Cl	0	0
13.6072	-33.8832	-0.0039	C	0	0
15.0610	-33.9867	0.0046	C	0	0
15.8064	-32.8847	-0.0143	C	0	0
15.1526	-31.5615	-0.0451	C	0	0
13.8195	-31.4702	-0.0543	C	0	0
17.2099	-32.9918	-0.0048	N	0	0
17.8360	-34.2884	0.0222	C	0	0
18.0248	-31.8111	-0.0217	C	0	0
9.3539	-29.0878	-0.4169	C	0	0
9.2514	-27.6088	0.0541	C	0	0
7.9165	-27.0305	-0.1991	N	0	0
7.0098	-27.7934	0.6420	C	0	0
6.9124	-29.2343	0.1235	C	0	0
7.9637	-25.5969	0.2200	C	0	0
6.6425	-24.8092	0.1406	C	0	0
6.6850	-23.5909	0.3366	O	0	0
5.4605	-25.3979	-0.0914	N	0	0
4.2458	-24.6576	-0.1230	C	0	0
4.1282	-22.9202	-0.0035	S	0	0
2.3936	-22.9797	-0.1027	C	0	0
2.0031	-24.2546	-0.2168	C	0	0
3.0955	-25.2028	-0.2254	N	0	0

1	2	2	0	0	0
2	3	1	0	0	0
3	4	2	0	0	0
4	5	1	0	0	0
5	6	2	0	0	0
6	1	1	0	0	0
4	7	1	0	0	0
7	8	1	0	0	0
8	9	2	0	0	0
9	5	1	0	0	0
8	10	1	0	0	0
6	11	1	0	0	0
1	12	1	0	0	0
10	13	2	0	0	0
13	14	1	0	0	0
14	15	2	0	0	0
15	16	1	0	0	0
16	17	2	0	0	0
17	10	1	0	0	0
15	18	1	0	0	0
18	19	1	0	0	0
18	20	1	0	0	0
11	21	1	0	0	0
21	22	1	0	0	0
22	23	1	0	0	0
23	24	1	0	0	0
24	25	1	0	0	0
25	11	1	0	0	0

```
 23 26  1  0  0  0
 26 27  1  0  0  0
 27 28  2  0  0  0
 27 29  1  0  0  0
 29 30  1  0  0  0
 30 31  1  0  0  0
 31 32  1  0  0  0
 32 33  2  0  0  0
 33 34  1  0  0  0
 34 30  2  0  0  0
M  END
> <Clean Energy>
46.0117

$$$$
CCT137690
  DSViewer          3D                             0

 36 41  0  0  0  0  0  0  0  0999 V2000
   12.1167  -17.8968   -0.0179 C   0  0
   12.3074  -19.2198   -0.0431 C   0  0
   13.5798  -19.7789   -0.0349 N   0  0
   14.5872  -19.0190    0.0007 C   0  0
   14.5349  -17.6179    0.0328 C   0  0
   13.3594  -16.9547    0.0267 C   0  0
   15.8820  -19.4056    0.0059 N   0  0
   16.6338  -18.2119    0.0350 C   0  0
   15.8856  -17.1564    0.0539 N   0  0
   18.1052  -18.2422    0.0295 C   0  0
   18.7105  -19.4296    0.0023 C   0  0
   20.1676  -19.5234   -0.0176 C   0  0
   20.9074  -18.4171   -0.0106 C   0  0
   20.2433  -17.0979    0.0218 C   0  0
   18.9090  -17.0153    0.0413 C   0  0
   22.3160  -18.5159   -0.0404 N   0  0
   22.9285  -19.8332   -0.1656 C   0  0
   24.4070  -19.8519    0.2751 C   0  0
   25.1323  -18.7241   -0.3096 N   0  0
   24.5762  -17.5154    0.3020 C   0  0
   23.1103  -17.3043   -0.1353 C   0  0
   26.5496  -18.8317    0.0658 C   0  0
   10.2745  -17.3449   -0.0272 Br  0  0
   13.2734  -15.4624    0.0516 N   0  0
   14.4571  -14.6434    0.3163 C   0  0
   14.3259  -13.1808   -0.2061 C   0  0
   13.0210  -12.5908    0.1402 N   0  0
   12.0382  -13.3772   -0.5867 C   0  0
   11.9874  -14.7996   -0.0204 C   0  0
   12.9973  -11.1746   -0.3289 C   0  0
   11.6710  -10.4542   -0.0645 C   0  0
   11.4545   -9.0789   -0.3307 C   0  0
   10.2026   -8.8765    0.0388 C   0  0
```

```
    9.5931  -10.0572    0.5315 O    0  0
   10.6139  -10.9755    0.4204 N    0  0
    9.5063   -7.5478   -0.0499 C    0  0
  1  2  2  0  0  0
  2  3  1  0  0  0
  3  4  2  0  0  0
  4  5  1  0  0  0
  5  6  2  0  0  0
  6  1  1  0  0  0
  4  7  1  0  0  0
  7  8  1  0  0  0
  8  9  2  0  0  0
  9  5  1  0  0  0
  8 10  1  0  0  0
 10 11  2  0  0  0
 11 12  1  0  0  0
 12 13  2  0  0  0
 13 14  1  0  0  0
 14 15  2  0  0  0
 15 10  1  0  0  0
 13 16  1  0  0  0
 16 17  1  0  0  0
 17 18  1  0  0  0
 18 19  1  0  0  0
 19 20  1  0  0  0
 20 21  1  0  0  0
 21 16  1  0  0  0
 19 22  1  0  0  0
  1 23  1  0  0  0
  6 24  1  0  0  0
 24 25  1  0  0  0
 25 26  1  0  0  0
 26 27  1  0  0  0
 27 28  1  0  0  0
 28 29  1  0  0  0
 29 24  1  0  0  0
 27 30  1  0  0  0
 30 31  1  0  0  0
 31 32  1  0  0  0
 32 33  2  0  0  0
 33 34  1  0  0  0
 34 35  1  0  0  0
 35 31  2  0  0  0
 33 36  1  0  0  0
M  END
> <Clean Energy>
50.2654

$$$$
GSK650394
  DSViewer          3D                              0
```

```
29 33  0  0  0  0  0  0  0  0999 V2000
   28.4360  -44.6472    0.0166 C   0  0
   28.5244  -46.1099    0.0096 C   0  0
   29.7162  -46.7054    0.0143 C   0  0
   30.9406  -45.8985    0.0264 C   0  0
   30.8595  -44.5680    0.0326 C   0  0
   29.5476  -43.9132    0.0278 C   0  0
   32.2518  -46.5536    0.0270 C   0  0
   32.3393  -47.8919    0.0134 C   0  0
   33.6110  -48.5314    0.0051 N   0  0
   34.6588  -47.8103    0.0114 C   0  0
   34.6259  -46.4237    0.0279 C   0  0
   33.4831  -45.7517    0.0349 C   0  0
   36.5127  -44.5461    0.0087 C   0  0
   37.8236  -44.2962   -0.0753 C   0  0
   38.3410  -42.8980   -0.1326 C   0  0
   37.4490  -41.8758   -0.1061 C   0  0
   36.0263  -42.1835    0.0168 C   0  0
   35.5878  -43.4324    0.0670 C   0  0
   37.7647  -40.4107   -0.2221 C   0  0
   36.8317  -39.6070   -0.1233 O   0  0
   39.0294  -39.9169   -0.5193 O   0  0
   39.8680  -42.7137   -0.2497 C   0  0
   40.6409  -44.0221   -0.1841 C   0  0
   42.1098  -43.5117   -0.1534 C   0  0
   42.0097  -42.0859    0.4895 C   0  0
   40.5336  -41.9803    0.9190 C   0  0
   35.9893  -48.2310   -0.0031 N   0  0
   36.8136  -47.0381   -0.0045 C   0  0
   36.0188  -45.9598    0.0191 C   0  0
  1  2  1  0  0  0
  2  3  2  0  0  0
  3  4  1  0  0  0
  4  5  2  0  0  0
  5  6  1  0  0  0
  6  1  2  0  0  0
  4  7  1  0  0  0
  7  8  2  0  0  0
  8  9  1  0  0  0
  9 10  2  0  0  0
 10 11  1  0  0  0
 11 12  2  0  0  0
 12  7  1  0  0  0
 13 14  2  0  0  0
 14 15  1  0  0  0
 15 16  2  0  0  0
 16 17  1  0  0  0
 17 18  2  0  0  0
 18 13  1  0  0  0
 16 19  1  0  0  0
 19 20  2  0  0  0
 19 21  1  0  0  0
```

```
 15 22  1  0  0  0
 22 23  1  0  0  0
 23 24  1  0  0  0
 24 25  1  0  0  0
 25 26  1  0  0  0
 26 22  1  0  0  0
 10 27  1  0  0  0
 27 28  1  0  0  0
 28 29  2  0  0  0
 29 11  1  0  0  0
 29 13  1  0  0  0
M  END
> <Clean Energy>
50.553

$$$$
ABT-348
  DSViewer          3D                              0

 35 39  0  0  0  0  0  0  0  0999 V2000
   41.9345  -64.1487    0.0002 C    0  0
   42.3224  -65.5700    0.0002 C    0  0
   43.6052  -65.9225    0.0001 C    0  0
   44.6442  -64.8951    0.0001 C    0  0
   44.3039  -63.6074    0.0002 C    0  0
   42.8880  -63.2177    0.0002 C    0  0
   43.9422  -67.2206    0.0001 F    0  0
   40.5668  -63.7393    0.0002 N    0  0
   39.4658  -64.6443    0.0005 C    0  0
   38.1203  -64.1984    0.0005 N    0  0
   39.6525  -65.8652    0.0008 O    0  0
   37.6750  -62.8525    0.0001 C    0  0
   38.5056  -61.8105   -0.0006 C    0  0
   37.9643  -60.4414   -0.0010 C    0  0
   36.6455  -60.2279   -0.0006 C    0  0
   35.7589  -61.3749    0.0002 C    0  0
   36.2391  -62.6136    0.0005 C    0  0
   36.1566  -58.8053   -0.0008 C    0  0
   37.0656  -57.8223   -0.0012 C    0  0
   36.2879  -56.2537   -0.0011 S    0  0
   34.6795  -56.9274   -0.0004 C    0  0
   34.6980  -58.2523   -0.0003 C    0  0
   33.4160  -56.1801    0.0001 C    0  0
   32.2820  -56.8781    0.0005 C    0  0
   32.2968  -58.2941    0.0005 N    0  0
   33.3798  -58.9729    0.0001 C    0  0
   33.3561  -54.6992    0.0004 C    0  0
   34.4899  -53.7881    0.0005 C    0  0
   34.0259  -52.6002    0.0009 N    0  0
   32.6823  -52.6618    0.0010 N    0  0
   32.2395  -53.9865    0.0007 C    0  0
   31.8311  -51.4996    0.0010 C    0  0
```

```
   30.3457  -51.9035   -0.0005 C   0  0
   29.5386  -50.7220   -0.0005 O   0  0
   33.2710  -60.3486    0.0001 N   0  0
  1  2  1  0  0  0
  2  3  2  0  0  0
  3  4  1  0  0  0
  4  5  2  0  0  0
  5  6  1  0  0  0
  6  1  2  0  0  0
  3  7  1  0  0  0
  1  8  1  0  0  0
  8  9  1  0  0  0
  9 10  1  0  0  0
  9 11  2  0  0  0
 10 12  1  0  0  0
 12 13  2  0  0  0
 13 14  1  0  0  0
 14 15  2  0  0  0
 15 16  1  0  0  0
 16 17  2  0  0  0
 17 12  1  0  0  0
 15 18  1  0  0  0
 18 19  2  0  0  0
 19 20  1  0  0  0
 20 21  1  0  0  0
 21 22  2  0  0  0
 22 18  1  0  0  0
 21 23  1  0  0  0
 23 24  2  0  0  0
 24 25  1  0  0  0
 25 26  2  0  0  0
 26 22  1  0  0  0
 23 27  1  0  0  0
 27 28  1  0  0  0
 28 29  2  0  0  0
 29 30  1  0  0  0
 30 31  1  0  0  0
 31 27  2  0  0  0
 30 32  1  0  0  0
 32 33  1  0  0  0
 33 34  1  0  0  0
 26 35  1  0  0  0
M  END
> <Clean Energy>
37.714

$$$$
Barasertib
  DSViewer          3D                             0

 37 40  0  0  0  0  0  0  0  0999 V2000
   32.5270   31.1120   65.3050 C   0  0
```

32.2690	32.2650	65.9990	C	0	0
33.2000	33.5840	65.7350	F	0	0
31.2010	32.2310	66.8680	C	0	0
30.4380	31.0680	67.0530	C	0	0
30.7300	29.9040	66.3240	C	0	0
31.7970	29.9120	65.4830	C	0	0
32.0340	28.8450	64.7440	N	0	0
32.9570	28.8630	63.7820	C	0	0
33.8900	29.6780	63.7720	O	0	0
32.8960	27.6130	62.8170	C	0	0
34.2960	27.7230	62.0500	C	0	0
35.2190	26.7600	61.8620	C	0	0
34.7600	28.8220	61.4610	N	0	0
36.0130	28.5410	60.9260	N	0	0
36.2940	27.2430	61.1920	C	0	0
37.4880	26.6380	60.8010	N	0	0
37.6460	25.2750	60.7470	C	0	0
36.8640	24.5180	61.5640	N	0	0
37.0040	23.1840	61.4170	C	0	0
37.9660	22.5560	60.6420	N	0	0
38.8020	23.2710	59.8280	C	0	0
39.7050	22.5990	58.9080	C	0	0
38.6570	24.6980	59.8930	C	0	0
39.5060	25.4290	59.0080	C	0	0
40.4260	24.7750	58.1760	C	0	0
40.5780	23.3830	58.1480	C	0	0
41.5500	22.8750	57.3060	O	0	0
42.2750	21.6740	57.4800	C	0	0
42.1010	21.0780	56.0930	C	0	0
43.4310	20.6320	55.5200	C	0	0
43.2650	20.6530	54.0380	N	0	0
44.0810	19.6140	53.3780	C	0	0
43.1770	18.3790	53.1460	C	0	0
43.5660	22.0130	53.5480	C	0	0
43.0520	22.1710	52.1070	C	0	0
42.1460	23.3070	52.1060	O	0	0

1	2	4	0	0	0
1	7	4	0	0	0
2	3	1	0	0	0
2	4	4	0	0	0
4	5	4	0	0	0
5	6	4	0	0	0
6	7	4	0	0	0
7	8	1	0	0	0
8	9	1	0	0	0
9	10	2	0	0	0
9	11	1	0	0	0
11	12	1	0	0	0
12	13	2	0	0	0
12	14	1	0	0	0
13	16	1	0	0	0
14	15	1	0	0	0

```
 15 16  2  0  0  0
 16 17  1  0  0  0
 17 18  1  0  0  0
 18 19  4  0  0  0
 18 24  4  0  0  0
 19 20  4  0  0  0
 20 21  4  0  0  0
 21 22  4  0  0  0
 22 23  4  0  0  0
 22 24  4  0  0  0
 23 27  4  0  0  0
 24 25  4  0  0  0
 25 26  4  0  0  0
 26 27  4  0  0  0
 27 28  1  0  0  0
 28 29  1  0  0  0
 29 30  1  0  0  0
 30 31  1  0  0  0
 31 32  1  0  0  0
 32 33  1  0  0  0
 32 35  1  0  0  0
 33 34  1  0  0  0
 35 36  1  0  0  0
 36 37  1  0  0  0
M  END
> <Resolution>
1.49

$$$$
```
