# Supplementary material for: Aurora B Inhibitors as Cancer Therapeutics
Source: Molecules. 2023 Apr 11;28(8):3385. doi: 10.3390/molecules28083385 (PMC10144992; doi:10.3390/molecules28083385)
Supplement: Supplementary file 1 [file molecules-28-03385-s001.zip › molecules-2259316-supplementary Table S1.pdf]

Aurora B Inhibitors as Cancer Therapeutics

Antal H. Kovacs [1], Dong Zhao [1] and Jinqiang Hou [1,2,*]

[1] Department of Chemistry, Lakehead University, 955 Oliver Road, Thunder Bay, ON P7B 5E1, Canada
[2] Thunder Bay Regional Health Research Institute, 980 Oliver Road, Thunder Bay, ON P7B 6V4, Canada
* Correspondence: jhou3@lakeheadu.ca

Table S1: Comprehensive list of IC_{50} data for Aurora B inhibitors reported in literature

AZD1152 *in vitro* IC_{50} values

Malignancy	Cell Line	IC_{50} (nM)	Reference
Adult lymphoblastic Leukemia: Philadelphia chromosome-positive	PALL-2	5	1
Acute monocytic leukemia	MOLM13	12	1
Biphenotypic leukemia	MV4-11	8	1
Androgen-sensitive human prostatic adenocarcinoma	LNCaP	25	2
Androgen-independent advanced human prostatic adenocarcinoma	PC-3	10	2
Human retinoblastoma	Y-79	39.38	3
Patient-derived retinoblastoma cells	LRB1	38.41	3
Patient-derived retinoblastoma cells	LRB2	32.39	3
Hepatocellular carcinoma	Huh-7	16.72	4
Hepatocellular carcinoma	Hep3B	4.79	4
Lung adenocarcinoma	A549	0.9	5
Lung adenocarcinoma	A549	20.16	6

Malignancy	Cell Line	IC_{50} (nM)	Reference
Lung squamous cell carcinoma	SK-MES1	1.2	5
Lung adenocarcinoma	SKLU1	2.3	5
Osteosarcoma	U2OS	146	7
Undifferentiated human liposarcoma	SW-872	43.4	8
Differentiated human liposarcoma	93T449	74.5	8
Colon carcinoma	HCT116	20.12	6
Colorectal adenocarcinoma	HT-29	150	6
Anaplastic osteosarcoma	SK-ES-1	5	9
Anaplastic osteosarcoma	A4573	6	9
Anaplastic osteosarcoma	A673	5000	9
Breast adenocarcinoma	MDA-MB-468	14	10
Amelanotic melanoma	MDA-MB-435	125	10
Breast adenocarcinoma	MDA-MB-231	105	10
Breast adenocarcinoma	MDA-MB-361	70	10
Breast carcinoma	BT-474	8	10
Anaplastic thyroid carcinoma	CAL-62	9.2	11
Anaplastic thyroid carcinoma	BHT-101	461.3	11
Anaplastic thyroid carcinoma	8305C	287.8	11

Malignancy	Cell Line	IC_{50} (nM)	Reference
Anaplastic thyroid carcinoma	8505C	26.7	[11]
Hereditary thyroid medullary carcinoma	TT	401.6	[12]
Adult hepatocellular carcinoma	JHH-1	17.4	[13]
Adult hepatocellular carcinoma	JHH-2	218.0	[13]
Adult hepatocellular carcinoma	JHH-4	155.6	[13]
Adult hepatocellular carcinoma	HuH-1	27.3	[13]
Hepatoblastoma	HuH-6	3.7	[13]
Adult hepatocellular carcinoma	HuH-7	6.8	[13]
Adult hepatocellular carcinoma	HLE	45.9	[13]
Adult hepatocellular carcinoma	HLF	126.1	[13]
Adult hepatocellular carcinoma	PLC/PRF/5	76.9	[13]
Hepatic epithelial neoplasm	SK-Hep1	21.9	[13]
Pediatric hepatocellular carcinoma	Hep-3B	7.6	[13]
Hepatoblastoma	Hep-G2	14.7	[13]
Gastric carcinoma	HGC-27	3.98	[14]
Gastric mucinous adenocarcinoma	MGC-803	2.82	[14]

GSK1070916 *in vitro* IC_{50} values

Malignancy	Cell Line	IC_{50} (nM)	Reference
Endocervical adenocarcinoma (HPV related)	KB-3-1	16	15
Endocervical adenocarcinoma (HPV related)	KB-C2	1359	15
Colon adenocarcinoma	SW620	96	15
Colon adenocarcinoma	SW620/AD300	1460	15
Embryonic kidney cells	HEK293/pcDNA3.1	435	15
Embryonic kidney cells	HEK293/ABCB1	3331	15
Human retinoblastoma	Y-79	17.89	3
Patient-derived retinoblastoma cells	LRB1	5.97	3
Patient-derived retinoblastoma cells	LRB2	5.88	3
Osteosarcoma	U2OS	34 (EC_{50})	16
B-cell chronic leukemia	MEC-1	10 (EC_{50})	16
Primary cutaneous T-cell non-Hodgkin lymphoma	MJ	7 (EC_{50})	16
Cutaneous T-cell lymphoma (Sézary syndrome)	HuT 78	14 (EC_{50})	16
Primary cutaneous T-cell non-Hodgkin lymphoma	HH	18 (EC_{50})	16
Precursor T-cell acute lymphoblastic leukemia	ALL-SIL	2 (EC_{50})	16
Precursor T-cell acute lymphoblastic leukemia	MOLT-16	3 (EC_{50})	16

Malignancy	Cell Line	IC_{50} (nM)	Reference
Precursor T-cell acute lymphoblastic leukemia	HSB-2	3 (EC_{50})	[16]
Chronic myelogenous leukemia	CML-T1	3 (EC_{50})	[16]
Precursor T-cell acute lymphoblastic leukemia	MOLT-4	3 (EC_{50})	[16]
Precursor T-cell acute lymphoblastic leukemia	Jurkat	3 (EC_{50})	[16]
Precursor T-cell acute lymphoblastic leukemia	CTV-1	4 (EC_{50})	[16]
Precursor T-cell acute lymphoblastic leukemia	SKW-3	5 (EC_{50})	[16]
Precursor T-cell acute lymphoblastic leukemia	MOLT-3	5 (EC_{50})	[16]
Precursor T-cell acute lymphoblastic leukemia	CEM/C1	6 (EC_{50})	[16]
Precursor T-cell acute lymphoblastic leukemia	CCRF-CEM	7 (EC_{50})	[16]
Precursor T-cell acute lymphoblastic leukemia	JRT3-T3.5	9 (EC_{50})	[16]
Precursor T-cell acute lymphoblastic leukemia	DND-41	787 (EC_{50})	[16]
Chronic myeloid leukemia	EM-2	4 (EC_{50})	[16]
Chronic myeloid leukemia	EM-3	4 (EC_{50})	[16]
Chronic myeloid leukemia	BV173	5 (EC_{50})	[16]
Chronic myeloid leukemia	KCL-22	5 (EC_{50})	[16]
Chronic myeloid leukemia	KU812	8 (EC_{50})	[16]
Blast phase chronic myelogenous leukemia	K-562	13 (EC_{50})	[16]

Malignancy	Cell Line	IC_{50} (nM)	Reference
Blast phase chronic myelogenous leukemia	MEG-01	44 (EC_{50})	[16]
Acute myeloid leukemia	PLB-985	1 (EC_{50})	[16]
Acute monoblastic/monocytic leukemia	NOMO-1	2 (EC_{50})	[16]
Precursor T-cell acute lymphoblastic leukemia	CCRF-SB	2 (EC_{50})	[16]
Acute myeloid leukemia	OCI-AML-2	4 (EC_{50})	[16]
Acute myeloid leukemia	OCI-AML-3	4 (EC_{50})	[16]
Acute myeloid leukemia	ML-2	5 (EC_{50})	[16]
Acute monoblastic/monocytic leukemia	THP-1	5 (EC_{50})	[16]
Acute monoblastic/monocytic leukemia	MV-4-11	7 (EC_{50})	[16]
Acute myeloid leukemia	HL-60	10 EC_{50})	[16]
Myelodysplastic syndrome	F-36P	10 (EC_{50})	[16]
Acute promyelocytic leukemia	NB4	11 (EC_{50})	[16]
Acute megakaryoblastic leukemia	M-07e	13 (EC_{50})	[16]
Acute myeloid leukemia	OCI-M1	20 (EC_{50})	[16]
Acute myeloid leukemia	GDM-1	21 (EC_{50})	[16]
Acute myeloid leukemia	BDCM	26 (EC_{50})	[16]
Acute megakaryoblastic	CMK	27 (EC_{50})	[16]

Malignancy	Cell Line	IC_{50} (nM)	Reference
leukemia in Down syndrome			
Acute myeloid leukemia	KG-1	32 (EC_{50})	[16]
Acute erythroid leukemia	HEL 92.1.7	60 (EC_{50})	[16]
B-cell prolymphocytic leukemia	JVM-3	2 (EC_{50})	[16]
B-lymphoblastic leukemia/lymphoma	SUP-B15	2 (EC_{50})	[16]
Precursor B-cell acute lymphoblastic leukemia	NALM-6	2 (EC_{50})	[16]
Diffuse large B-cell lymphoma	KARPAS-231	3 (EC_{50})	[16]
B-lymphoblastic leukemia/lymphoma	SEM	3 (EC_{50})	[16]
B-lymphoblastic leukemia/lymphoma	RCH-ACV	4 (EC_{50})	[16]
Acute myeloid leukemia	CESS	5 (EC_{50})	[16]
B-lymphoblastic leukemia/lymphoma	Kasumi-2	6 (EC_{50})	[16]
Precursor B-cell acute lymphoblastic leukemia	TANOUE	1478 (EC_{50})	[16]
Acute myeloid leukemia	HD-MY-Z	4 (EC_{50})	[16]
B-lymphoblastoid	Hs 445	5 (EC_{50})	[16]
B-lymphoblastoid	RPMI 6666	10 (EC_{50})	[16]
Hodgkin lymphoma	L-428	31 (EC_{50})	[16]
Hodgkin lymphoma	TO 175.t	54 (EC_{50})	[16]
Burkitt lymphoma	ST486	2 (EC_{50})	[16]

Malignancy	Cell Line	IC_{50} (nM)	Reference
EBV-related Burkitt lymphoma	EB2	2 (EC_{50})	[16]
EBV-related Burkitt lymphoma	Raji	3 (EC_{50})	[16]
Burkitt lymphoma	GA10	4 (EC_{50})	[16]
EBV-related Burkitt lymphoma	Daudi	5 (EC_{50})	[16]
EBV-related Burkitt lymphoma	Jiyoye	5 (EC_{50})	[16]
Burkitt lymphoma	CA46	5 (EC_{50})	[16]
EBV-related Burkitt lymphoma	Namalwa	6 (EC_{50})	[16]
EBV-related Burkitt lymphoma	NC-37	7 (EC_{50})	[16]
EBV-related Burkitt lymphoma	EB1	7 (EC_{50})	[16]
EBV-related Burkitt lymphoma	EB3	7 (EC_{50})	[16]
EBV-related Burkitt lymphoma	P3HR-1	8 (EC_{50})	[16]
B-cell non-Hodgkin lymphoma	MC116	8 (EC_{50})	[16]
B-lymphoblastoid	1A2	9 (EC_{50})	[16]
EBV-related Burkitt lymphoma	HS-Sultan	14 (EC_{50})	[16]
Burkitt lymphoma	DG-75	18 (EC_{50})	[16]
Primary effusion lymphoma	CRO-AP2	3 (EC_{50})	[16]
Primary effusion lymphoma	CRO-AP5	3 (EC_{50})	[16]

Malignancy	Cell Line	IC_{50} (nM)	Reference
ALK-positive anaplastic large cell lymphoma	SR	3 (EC_{50})	[16]
Diffuse large B-cell lymphoma activated B-cell type	NU-DUL-1	4 (EC_{50})	[16]
B-cell non-Hodgkin lymphoma	MHH-PREB-1	4 (EC_{50})	[16]
Diffuse large B-cell lymphoma germinal centre B-cell type	OCI-LY-19	4 (EC_{50})	[16]
B-cell non-Hodgkin lymphoma	SU-DHL-16	4 (EC_{50})	[16]
Diffuse large B-cell lymphoma germinal centre B-cell type	Pfeiffer	4 (EC_{50})	[16]
Acute monoblastic/monocytic leukemia	U-937	5 (EC_{50})	[16]
Diffuse large B-cell lymphoma germinal centre B-cell type	SU-DHL-5	5 (EC_{50})	[16]
Diffuse large B-cell lymphoma germinal centre B-cell type	SU-DHL-4	5 (EC_{50})	[16]
Diffuse large B-cell lymphoma germinal centre B-cell type	SU-DHL-6	6 (EC_{50})	[16]
Diffuse large B-cell lymphoma germinal centre B-cell type	HT	7 (EC_{50})	[16]
Precursor B-cell acute lymphoblastic leukemia	JM-1	7 (EC_{50})	[16]
Diffuse large B-cell lymphoma germinal centre B-cell type	Farage	7 (EC_{50})	[16]

Malignancy	Cell Line	IC_{50} (nM)	Reference
B-lymphoblastoid	MC/CAR	9 (EC_{50})	[16]
Diffuse large B-cell lymphoma	RL	10 (EC_{50})	[16]
Diffuse large B-cell lymphoma germinal centre B-cell type	DB	11 (EC_{50})	[16]
Follicular lymphoma	Sc-1	12 (EC_{50})	[16]
Diffuse large B-cell lymphoma germinal centre B-cell type	DoHH-2	14 (EC_{50})	[16]
Diffuse large B-cell lymphoma germinal centre B-cell type	Toledo	15 (EC_{50})	[16]
Diffuse large B-cell lymphoma germinal centre B-cell type	SU-DHL-10	15 (EC_{50})	[16]
B-lymphoblastoid	ARH-77	15 (EC_{50})	[16]
Primary effusion lymphoma	BC-1	18 (EC_{50})	[16]
Primary effusion lymphoma	BC-3	27 (EC_{50})	[16]
Primary effusion lymphoma	BCP-1	31 (EC_{50})	[16]
Diffuse large B-cell lymphoma germinal center B-cell type	RC-K8	38 (EC_{50})	[16]
Primary effusion lymphoma	BC-2	75 (EC_{50})	[16]
Mantle cell lymphoma	REC-1	77 (EC_{50})	[16]
B-lymphoblastoid	HuNS1	9 (EC_{50})	[16]

Malignancy	Cell Line	IC_{50} (nM)	Reference
Human myeloma	RPMI8266	11 (EC_{50})	[16]
Multiple myeloma	SKO-007	353 (EC_{50})	[16]
Multiple myeloma	U266B1	422(EC_{50})	[16]
Minimally invasive lung adenocarcinoma	NCI-H358	4 (EC_{50})	[16]
Lung adenocarcinoma	A-549	7 (EC_{50})	[16]
Lung squamous cell carcinoma	NCI-H157	8 (EC_{50})	[16]
Colon adenocarcinoma	MV-522	13 (EC_{50})	[16]
Lung large cell carcinoma	NCI-H460	14 (EC_{50})	[16]
Lung large cell carcinoma	NCI-H1299	23 (EC_{50})	[16]
Lung large cell carcinoma	NCI-H1155	40 (EC_{50})	[16]
Endometrial adenocarcinoma	HEC-1-B	4 (EC_{50})	[16]
Melanoma	SK-MEL-2	2 (EC_{50})	[16]
Amelanotic melanoma	A375-P	7 (EC_{50})	[16]
Cutaneous melanoma	SK-MEL-28	7 (EC_{50})	[16]
Cutaneous melanoma	SK-MEL-5	8 (EC_{50})	[16]
Vulvar squamous cell carcinoma	SW954	11 (EC_{50})	[16]
Prostate carcinoma	PC-3	11 (EC_{50})	[16]
Prostate carcinoma	LNCaP	12 (EC_{50})	[16]

Malignancy	Cell Line	IC_{50} (nM)	Reference
Prostate carcinoma	DU145	15 (EC_{50})	[16]
Pancreatic ductal adenocarcinoma	AsPC-1	2 (EC_{50})	[16]
Pancreatic ductal adenocarcinoma	Mia PaCa-2	3 (EC_{50})	[16]
Pancreatic ductal adenocarcinoma	BxPC-3	3 (EC_{50})	[16]
Pancreatic ductal adenocarcinoma	PANC-1	13 (EC_{50})	[16]
High grade ovarian serous adenocarcinoma	OVCAR-3	3 (EC_{50})	[16]
Ovarian endometrioid adenocarcinoma	A2780	5 (EC_{50})	[16]
High grade ovarian serous adenocarcinoma	OVCAR-4	11 (EC_{50})	[16]
Ovarian serous cystadenocarcinoma	SK-OV-3	12 (EC_{50})	[16]
High grade ovarian serous adenocarcinoma	OVCAR-8	30 (EC_{50})	[16]
Pediatric hepatocellular carcinoma	Hep-3B	12 (EC_{50})	[16]
Renal cell carcinoma	A-498	33 (EC_{50})	[16]
Renal cell carcinoma	786-O	57 (EC_{50})	[16]
Tongue squamous cell carcinoma	HN-5	10 (EC_{50})	[16]
Endocervical adenocarcinoma (HPV-related)	HeLa	13 (EC_{50})	[16]
Breast adenocarcinoma	SK-BR-3	4 (EC_{50})	[16]
Breast adenocarcinoma	MDA-MB-453	6 (EC_{50})	[16]

Malignancy	Cell Line	IC_{50} (nM)	Reference
Breast carcinoma	MX-1	8 (EC_{50})	[16]
Breast adenocarcinoma	MDA-MB-231	11 (EC_{50})	[16]
Breast adenocarcinoma	MDA-MB-468	12 (EC_{50})	[16]
Invasive breast carcinoma of no special type	MCF-7	13 (EC_{50})	[16]
Invasive breast carcinoma of no special type	T-47D	13 (EC_{50})	[16]
Colon adenocarcinoma	SW48	2 (EC_{50})	[16]
Colon adenocarcinoma	COLO 201	2 (EC_{50})	[16]
Colon adenocarcinoma	SW480	3 (EC_{50})	[16]
Colon adenocarcinoma	WiDr	3 (EC_{50})	[16]
Colon adenocarcinoma	COLO 205	7 (EC_{50})	[16]
Colon carcinoma	RKO-E6	7 (EC_{50})	[16]
Colon carcinoma	RKO	7 (EC_{50})	[16]
Colon adenocarcinoma	LOVO	7 (EC_{50})	[16]
Colon carcinoma	HCT116	8 (EC_{50})	[16]
Colon adenocarcinoma	SW620	8 (EC_{50})	[16]
Colon adenocarcinoma	HT29	10 (EC_{50})	[16]
Colon adenocarcinoma	SW1417	19 (EC_{50})	[16]
Colon adenocarcinoma	DLD-1	28 (EC_{50})	[16]

Malignancy	Cell Line	IC_{50} (nM)	Reference
Colon adenocarcinoma	HCT 8	38 (EC_{50})	16
Colon adenocarcinoma	COLO 320HSR	92 (EC_{50})	16
Colon adenocarcinoma	COLO 320DM	96 (EC_{50})	16
Rectal carcinoma	NCI-H630	127 (EC_{50})	16

PHA-739358 *in vitro* IC_{50} values

Malignancy	Cell Line	IC_{50} (nM)	Reference
Adult hepatocellular carcinoma	HuH-7	15	17
Hepatoblastoma	HepG2	20	17
Invasive breast carcinoma of no special type	T-47D	210	18
Acral lentiginous melanoma	WM3211	1760	19
Melanoma	1205Lu	3340	19
Cutaneous melanoma	SK-MEL-28	12450	19
Acute monoblastic/monocytic leukemia	THP-1	2690	20
Acute myeloid leukemia	HL-60	3250	20
Chronic myeloid leukemia	K-562	3020	20
Breast adenocarcinoma	MDA-MB-231	3090	21
Invasive breast carcinoma of no special type	MCF7	5140	21

Malignancy	Cell Line	IC_{50} (nM)	Reference
Cervical squamous cell carcinoma (HPV-related)	C13	1830	22
Acute myeloid leukemia	HL-60	3000	23
Chronic myeloid leukemia	K-562	200	23
Pediatric hepatocellular carcinoma	Hep3B	22030	24
Gastric tubular adenocarcinoma	NCI-N87	2770	25
Gastric adenocarcinoma	AGS	1450	25
Amelanotic melanoma	A375	254	26
Cutaneous melanoma	SK-MEL-5	1718	26

AT9283 *in vitro* IC_{50} values

Malignancy	Cell Line	IC_{50} (nM)	Reference
Chronic myeloid leukemia	BV-173	55	27
Chronic myeloid leukemia	Ku812	26	27
Chronic myeloid leukemia	MYL	21	27
Chronic myeloid leukemia	KT-1	81	27
Chronic myeloid leukemia	KBM-5	84	27
Blast phase chronic myelogenous leukemia	MEG-01	31	27
Blast phase chronic myelogenous leukemia	K-562	100 (polyploidy)	27
Chronic myeloid leukemia	KBM-5/STIR	16	27

Malignancy	Cell Line	IC_{50} (nM)	Reference
Chronic myeloid leukemia	BV-173/shBim	12	[27]
Acute myeloid leukemia	HL-60	30 (polyploidy)	[27]
Precursor T-cell acute lymphoblastic leukemia	Jurkat	500 (polyploidy)	[27]
Precursor T-cell acute lymphoblastic leukemia	NALM-6	140 (polyploidy)	[27]
Acute myeloid leukemia	KG-1a	55 (polyploidy)	[27]
EBV-related Burkitt lymphoma	Raji	370	[28]
Diffuse large B-cell lymphoma germinal centre B-cell type	Toledo	110	[28]
Mantle cell lymphoma	Granta-519	850	[28]
Diffuse large B-cell lymphoma germinal centre B-cell type	SU-DHL-4	280	[28]

AMG 900 *in vitro* IC_{50} values

Malignancy	Cell Line	IC_{50} (nM)	Reference
Liposarcoma	SW872	3.7 (EC_{50})	[8]
Well-differentiated liposarcoma	93T449	6.5 (EC_{50})	[8]
Colon carcinoma	HCT116	1.4 (EC_{50})	[8]
Breast ductal carcinoma	HCC1187	0.08	[29]
Breast adenocarcinoma	MDA-MB-468	0.07	[29]
Breast ductal carcinoma	HCC38	0.04	[29]

Malignancy	Cell Line	IC_{50} (nM)	Reference
Breast ductal carcinoma	HCC70	0.08	29
Breast ductal carcinoma	EFM-19	0.39	29
Invasive breast carcinoma of no special type	BT-20	0.46	29
Breast ductal carcinoma	HCC1395	0.5	29
Breast carcinoma	MDA-MB-157	0.45	29
Breast ductal carcinoma	HCC1569	0.1	29
Invasive breast lobular carcinoma	MDA-MB-134	0.3	29
Invasive breast carcinoma of no special type	UACC-893	2.12	29
Breast adenocarcinoma	MDA-MB-361	1.19	29
Breast adenocarcinoma	CAMA-1	0.38	29
Invasive breast carcinoma of no special type	BT-549	0.07	29
Invasive breast carcinoma of no special type	ZR-75-1	0.07	29
Invasive breast carcinoma of no special type	SUM225CWN	2.78	29
Breast carcinoma	UACC-732	0.7	29
Breast adenocarcinoma	MDA-MB-415	2.23	29
Breast adenocarcinoma	SK-BR-3	0.12	29
Invasive breast carcinoma of no special type	BT-474	0.11	29
Breast ductal carcinoma	HCC1954	0.13	29

Malignancy	Cell Line	IC_{50} (nM)	Reference
Amelanotic melanoma	MDA-MB-435	0.04	[29]
Breast ductal carcinoma	HCC1419	0.08	[29]
Invasive breast carcinoma of no special type	MDA-MB-436	1.64	[29]
Invasive breast carcinoma of no special type	Hs 578T	0.92	[29]
Breast carcinoma	CAL-51	1.17	[29]
Invasive breast carcinoma of no special type	MDA-MB-175-VII	0.19	[29]
Breast inflammatory carcinoma	SUM190PT	0.27	[29]
Breast squamous cell carcinoma, acantholytic variant	HCC1806	0.02	[29]
Breast carcinoma	EFM-192A	0.09	[29]
Non-malignant breast epithelial cells	184A1	1.18	[29]
Non-malignant breast epithelial cells	184B5	1.03	[29]
Breast carcinoma	COLO 824	0.49	[29]
Breast carcinoma	DU4475	1.15	[29]
Breast ductal carcinoma	HCC1143	0.03	[29]
Breast ductal carcinoma	HCC1937	0.06	[29]
Breast ductal carcinoma	HCC2218	2.05	[29]
Invasive breast carcinoma of no special type	KPL-1	0.88	[29]

Malignancy	Cell Line	IC_{50} (nM)	Reference
Non-malignant breast epithelial cells	MCF-10A	0.92	[29]
Invasive breast carcinoma of no special type	MCF-7	0.22	[29]
Breast adenocarcinoma	MDA-MB-231	0.25	[29]
Breast adenocarcinoma	MDA-MB-453	0.02	[29]
Invasive breast carcinoma of no special type	T-47D	0.46	[29]
Invasive breast carcinoma of no special type	UACC-812	15.21	[29]
Lung large cell carcinoma	NCI-H460 parental	3	[30]
Lung large cell carcinoma	NCI-H460-PTX paclitaxel resistant	3	[30]
Breast adenocarcinoma	MDA-MB-231 parental	2	[30]
Breast adenocarcinoma	MDA-MB-231-PTX paclitaxel resistant	3	[30]
Uterine corpus sarcoma	MES-SA parental	3	[30]
Uterine corpus sarcoma	MES-SA-Dx5 doxorubicin resistant	2	[30]
Plasma cell myeloma	U226-B1	2 (EC_{50})	[30]
Plasma cell myeloma	RPMI-8226	3 (EC_{50})	[30]

ZM447439 *in vitro* IC_{50} values

Malignancy	Cell Line	IC_{50} (nM)	Reference
Retinoblastoma	Y-79	845.27	[3]

Malignancy	Cell Line	IC_{50} (nM)	Reference
Patient-derived retinoblastoma cells	LRB1	641.47	[3]
Patient-derived retinoblastoma cells	LRB2	680.27	[3]
Lung adenocarcinoma	A549	3300	[31]
Lung large cell carcinoma	NCI-H1299	700	[31]
Invasive breast carcinoma of no special type	MCF-7	800	[31]
Hepatoblastoma	HepG2	600	[31]
Adult hepatocellular carcinoma	HLF	10 000	[31]
Canine leukemia	GL-1	4770	[32]
Canine lymphoma	EMA	8030	[32]
Osteosarcoma	U2OS	400	[33]
Osteosarcoma	SaOS-2	400	[33]
Osteosarcoma	IOR/OS18	2200	[33]
Osteosarcoma	IOR/OS9	200	[33]
Osteosarcoma	U2-OS/DX580	52 800	[33]
Osteosarcoma	Saos-2/DX580	16 300	[33]
Osteosarcoma	U2-OS/MTX300	600	[33]
Osteosarcoma	Saos-2/MTX300	300	[33]
Osteosarcoma	U2-OS/CDDP4	15 200	[33]

Malignancy	Cell Line	IC_{50} (nM)	Reference
Osteosarcoma	Saos-2/CDDP6	8400	[33]
Pancreatic ductal adenocarcinoma	Capan-1	1900	[34]
Normal human breast epithelial cell	MCF10A	2500	[34]
Normal human mammary epithelial cells (BRCA mutated)	BRCA2-999del5-N2	2700	[34]
Normal human mammary epithelial cells (BRCA mutated)	BRCA2-999del5-N1	2900	[34]
Normal human mammary epithelial cells (BRCA mutated)	BRCA2-999del5-T2	3900	[34]
Normal breast epithelial progenitor cell line	D492	3700	[34]
Normal breast epithelial cells (no mutations)	1585KT	4700	[34]
Invasive breast carcinoma of no special type	MCF7	4700	[34]
Normal breast epithelial cells (6474del4 BRCA2 mutation)	348T	5000	[34]
Invasive breast carcinoma of no special type	T-47D	5400	[34]
Normal breast epithelial cells (4700del4 BRCA2 mutation)	2981T	5600	[34]
Normal breast epithelial cells (no mutations)	1585T	6200	[34]
Normal breast epithelial cells (6474del4 BRCA2 mutation)	348KT	6700	[34]
Normal breast epithelial	2980T	7400	[34]

Malignancy	Cell Line	IC_{50} (nM)	Reference
cells (4700del4 BRCA2 mutation)			
Normal breast epithelial cells (no mutations)	3736T	8100	[34]

CYC116 *in vitro* IC_{50} values

Malignancy	Cell Line	IC_{50} (nM)	Reference
Lung adenocarcinoma	A549	480	[35]
Invasive breast carcinoma of no special type	MCF7	599	[35]
Endocervical adenocarcinoma (HPV-related)	HeLa	590	[35]
Colon adenocarcinoma	COLO205	241	[35]
Colon carcinoma	HCT116	340	[35]
Colon adenocarcinoma	HT29	725	[35]
Blast phase chronic myelogenous leukemia	K-562	1375	[35]
Precursor T-cell acute lymphoblastic leukemia	CCRF-CEM	471	[35]
Biphenotypic leukemia	MV4-11	34	[35]
Acute myeloid leukemia	HL-60	372	[35]
Lung large cell carcinoma	NCI-H460	681	[35]
Ovarian endometrioid adenocarcinoma	A2780	151	[35]
Pancreatic ductal adenocarcinoma	BxPC-3	1626	[35]

Malignancy	Cell Line	IC_{50} (nM)	Reference
Pancreatic adenocarcinoma	HuP-T4	775	[35]
Pancreatic ductal adenocarcinoma	MIA PaCa-2	308	[35]
Osteosarcoma	Saos-2	110	[35]
Uterine corpus sarcoma	MES-SA	90	[35]

VX-680 *in vitro* IC_{50} values

Malignancy	Cell Line	IC_{50} (nM)	Reference
Osteosarcoma	U2OS	2700	[33]
Osteosarcoma	Saos-2	2700	[33]
Osteosarcoma	IOR/OS18	5500	[33]
Osteosarcoma	IOR/OS9	700	[33]
Osteosarcoma	U2-OS/DX580	11 200	[33]
Osteosarcoma	Saos-2/DX580	13 800	[33]
Osteosarcoma	U2-OS/MTX300	4300	[33]
Osteosarcoma	Saos-2/MTX300	600	[33]
Osteosarcoma	U2-OS/CDDP4	4400	[33]
Osteosarcoma	Saos-2/CDDP6	2700	[33]
Endocervical adenocarcinoma (HPV-related)	HeLa	170	[36]

SNS-314 *in vitro* IC_{50} values

Malignancy	Cell Line	IC_{50} (nM)	Reference
Colon carcinoma	HCT116	6.4	[37]
Colon adenocarcinoma	HT29	24.0	[37]
Lung adenocarcinoma (non-small cell)	Calu-6	13.0	[37]
Lung large cell carcinoma	NCI-H1299	4.0	[37]
Prostate carcinoma	PC-3	4.4	[37]
Ovarian endometrioid adenocarcinoma	A2780	1.8	[37]
Breast adenocarcinoma	MDA-MB-231	8.1	[37]
Endocervical adenocarcinoma (HPV-related)	HeLa	9.3	[37]
Pancreatic ductal adenocarcinoma	MIA PaCa	9.1	[37]
Amelanotic melanoma	A375	5.9	[37]

BI 847325 *in vitro* IC_{50} values

Malignancy	Cell Line	IC_{50} (nM)	Reference
Amelanotic melanoma	A375	7.5 (GI_{50})	[38]
Lung adenocarcinoma (non-small cell)	Calu-6	60 (GI_{50})	[38]
Melanoma	M229	0.7	[39]
Melanoma (increased PDGFRβ expression mutation)	M229R	4	[39]

Malignancy	Cell Line	IC_{50} (nM)	Reference
Amelanotic melanoma	A-375	0.3	[39]
Melanoma (Ras mutation)	A-375R	0.5	[39]
Melanoma	WM793	2000	[39]
Melanoma (resistance mutation)	WM793R	230	[39]
Melanoma	1205Lu	140	[39]
Melanoma (resistance mutation)	1205LuR	320	[39]
Melanoma	M249	0.3	[39]
Melanoma (Ras mutation)	M249R	1	[39]
Cutaneous melanoma	WM164	5	[39]
Cutaneous melanoma (resistance mutation)	WM164R	80	[39]
Melanoma	WM39	0.6	[39]
Melanoma	RPMI17951	0.6	[39]

GSK650394 *in vitro* IC_{50} values

Malignancy	Cell Line	IC_{50} (nM)	Reference
Hepatoblastoma	HepG2	1530	[40]
Endocervical adenocarcinoma (HPV-related)	L-02	1750	[40]
Invasive breast carcinoma of no special type	MCF-7	1420	[40]

Malignancy	Cell Line	IC_{50} (nM)	Reference
Normal human embryonic fibroblast cells	HELF	6140	[40]
Endocervical adenocarcinoma (HPV-related)	HeLa	3570	[40]

SP-96 *in vitro* IC_{50} values

Malignancy	Cell Line	IC_{50} (nM)	Reference
Precursor T-cell acute lymphoblastic leukemia	CCRF-CEM	47.4 (GI_{50})	[41]
Acute myeloid leukemia	HL-60(TB)	5900 (GI_{50})	[41]
Blast phase chronic myelogenous leukemia	K-562	2140 (GI_{50})	[41]
Precursor T-cell acute lymphoblastic leukemia	MOLT-4	1950 (GI_{50})	[41]
Human myeloma	RPMI8226	1540 (GI_{50})	[41]
ALK-positive anaplastic large cell lymphoma	SR	1240 (GI_{50})	[41]
Lung adenocarcinoma	A-549/ATCC	1850 (GI_{50})	[41]
Lung adenocarcinoma	EKVX	6220 (GI_{50})	[41]
Lung adenocarcinoma	HOP-62	1840 (GI_{50})	[41]
Lung non-small cell carcinoma	HOP-92	2230 (GI_{50})	[41]
Pleural epithelioid mesothelioma	NCI-H226	12 100 (GI_{50})	[41]
Lung adenocarcinoma	NCI-H23	2990 (GI_{50})	[41]
Minimally invasive lung adenocarcinoma	NCI-H322M	3030 (GI_{50})	[41]

Malignancy	Cell Line	IC_{50} (nM)	Reference
Lung large cell carcinoma	NCI-H460	985 (GI_{50})	[41]
Lung adenocarcinoma	NCI-H522	1220 (GI_{50})	[41]
Colon adenocarcinoma	COLO205	50.3 (GI_{50})	[41]
Colon adenocarcinoma	HCC2998	4810 (GI_{50})	[41]
Colon carcinoma	HCT116	2040 (GI_{50})	[41]
Colon carcinoma	HCT 15	2500 (GI_{50})	[41]
Colon adenocarcinoma	HT29	1970 (GI_{50})	[41]
Colon carcinoma	KM12	2520 (GI_{50})	[41]
Colon adenocarcinoma	SW620	1900 (GI_{50})	[41]
Astrocytoma	SF268	1640 (GI_{50})	[41]
Glioblastoma	SF295	1810 (GI_{50})	[41]
Gliosarcoma	SF539	55.4 (GI_{50})	[41]
Astrocytoma	SNB-19	3160 (GI_{50})	[41]
Glioblastoma	SNB-75	39.1 (GI_{50})	[41]
Astrocytoma	U-251	1790 (GI_{50})	[41]
Amelanotic melanoma	LOX-IMVI	1550 (GI_{50})	[41]
Melanoma	Malme-3M	1290 (GI_{50})	[41]
Amelanotic melanoma	M14	2610 (GI_{50})	[41]
Amelanotic melanoma	MDA-MB-435	1490 (GI_{50})	[41]

Malignancy	Cell Line	IC_{50} (nM)	Reference
Melanoma	SK-MEL-2	4430 (GI_{50})	[41]
Cutaneous melanoma	SK-MEL-28	1550 (GI_{50})	[41]
Cutaneous melanoma	SK-MEL-5	1530 (GI_{50})	[41]
Melanoma	UACC-257	4500 (GI_{50})	[41]
Melanoma	UACC-62	3210 (GI_{50})	[41]
Ovarian endometrioid adenocarcinoma	IGROV-1	1300 (GI_{50})	[41]
High grade ovarian serous adenocarcinoma	OVCAR-3	1510 (GI_{50})	[41]
High grade ovarian serous adenocarcinoma	OVCAR-4	1850 (GI_{50})	[41]
High grade ovarian serous adenocarcinoma	OVCAR-5	10 300 (GI_{50})	[41]
High grade ovarian serous adenocarcinoma	OVCAR-8	2250 (GI_{50})	[41]
High grade ovarian serous adenocarcinoma	NCI/ADR-RES	10 800 (GI_{50})	[41]
Ovarian serous cystadenocarcinoma	SK-OV-3	610 (GI_{50})	[41]
Renal cell carcinoma	786-O	1870 (GI_{50})	[41]
Renal cell carcinoma	A-498	53.2 (GI_{50})	[41]
Papillary renal cell carcinoma	ACHN	2170 (GI_{50})	[41]
Clear cell renal cell carcinoma	Caki-1	560 (GI_{50})	[41]
Renal cell carcinoma	RXF 393L	1200 (GI_{50})	[41]
Renal cell carcinoma	SN12C	1600 (GI_{50})	[41]

Malignancy	Cell Line	IC_{50} (nM)	Reference
Clear cell renal cell carcinoma	TK-10	2720 (GI_{50})	[41]
Renal cell carcinoma	UO-31	2090 (GI_{50})	[41]
Prostate carcinoma	PC-3	2780 (GI_{50})	[41]
Prostate carcinoma	DU145	1860 (GI_{50})	[41]
Invasive breast carcinoma of no specific type	MCF7	1140 (GI_{50})	[41]
Invasive breast carcinoma of no specific type	MCF7	517	[41]
Breast adenocarcinoma	MDA-MB-231/ATCC	2880 (GI_{50})	[41]
Invasive breast carcinoma of no specific type	Hs 578T	1500 (GI_{50})	[41]
Invasive breast carcinoma of no specific type	BT-549	4040 (GI_{50})	[41]
Invasive breast carcinoma of no specific type	T-47D	1620 (GI_{50})	[41]
Breast adenocarcinoma	MDA-MB-468	107 (GI_{50})	[41]

PHA680632 *in vitro* IC_{50} values

Malignancy	Cell Line	IC_{50} (nM)	Reference
Cervical squamous cell carcinoma	C-33 A	320	[42]
Endocervical adenocarcinoma (HPV-related)	HeLa	410	[42]
Colon carcinoma	HCT116	60	[42]
Colon adenocarcinoma	HT29	1170	[42]

Malignancy	Cell Line	IC_{50} (nM)	Reference
Colon adenocarcinoma	LOVO	560	42
Lung adenocarcinoma	A549	620	42
Invasive breast carcinoma of no special type	MCF7	290	42
Ovarian endometrioid adenocarcinoma	A2780	110	42
Osteosarcoma	U2OS	1560	42
Prostate carcinoma	DU145	620	42
Acute monoblastic/monocytic leukemia	U-937	70	42
Acute myeloid leukemia	HL-60	130	42
Normal human dermal fibroblast tissue	NHDF	410	42

CCT129202 *in vitro* IC_{50} values

Malignancy	Cell Line	IC_{50} (nM)	Reference
Colon adenocarcinoma	COLO205	460 (GI_{50})	43
Colon adenocarcinoma	SW620	700 (GI_{50})	43
Colon carcinoma	HCT116	350 (GI_{50})	43
Colon adenocarcinoma	HT29	500 (GI_{50})	43
Colon carcinoma	KM12	1200 (GI_{50})	43
Endocervical adenocarcinoma (HPV-related)	HeLa	200 (GI_{50})	43

Malignancy	Cell Line	IC_{50} (nM)	Reference
Ovarian endometrioid adenocarcinoma	A2780	300 (GI_{50})	[43]
Ovarian endometrioid adenocarcinoma	OVCAR8	1000 (GI_{50})	[43]
Ovarian endometrioid adenocarcinoma	MDA-MB-157	1700 (GI_{50})	[43]
Biphenotypic leukemia	MV4-11	80 (GI_{50})	[43]
Epidermal carcinoma of the mouth	KB	1724	[44]
Endocervical adenocarcinoma (HPV-related)	KBv200	7023	[44]
Colon adenocarcinoma	S1	987	[44]
Colon adenocarcinoma	S1-M1-80	6130	[44]
Invasive breast carcinoma of no special type	MCF-7	3647	[44]
High grade ovarian serous adenocarcinoma	MCF-7/adr	25 660	[44]
Acute myeloid leukemia	HL-60	1464	[44]
Acute myeloid leukemia	HL-60/adr	6528	[44]

Reversine *in vitro* IC_{50} values

Malignancy	Cell Line	IC_{50} (nM)	Reference
Malignant neoplasms of the mouse mammary gland	4T1	130	[45]
Breast adenocarcinoma	MDA-MB-231	190	[45]
Invasive breast carcinoma of no special type	MCF-7	220	[45]

Malignancy	Cell Line	IC_{50} (nM)	Reference
Lung adenocarcinoma	A549	4000	46
Lung large cell carcinoma	NCI-H1299	20 000	46
Lung adenocarcinoma	NCI-H1435	900	46
Lung adenocarcinoma	NCI-H23	9700	46

CCT137690 *in vitro* IC_{50} values

Malignancy	Cell Line	IC_{50} (nM)	Reference
Colon adenocarcinoma	SW48	5 (GI_{50})	47
Colon adenocarcinoma	T84	140 (GI_{50})	47
Colon adenocarcinoma	SW620	150 (GI_{50})	47
Colon adenocarcinoma	LS174T	160 (GI_{50})	47
Colon adenocarcinoma	SW403	180 (GI_{50})	47
Colon adenocarcinoma	SW948	190 (GI_{50})	47
Colon carcinoma	HCT 116	220 (GI_{50})	47
Colon adenocarcinoma	DLD-1	260 (GI_{50})	47
Colon adenocarcinoma	COLO320	360 (GI_{50})	47
Colorectal carcinoma	PC/JW2	450 (GI_{50})	47
Colon adenocarcinoma	LOVO	470 (GI_{50})	47
Ovarian endometrioid adenocarcinoma	A2780	350 (GI_{50})	47

Malignancy	Cell Line	IC_{50} (nM)	Reference
Endocervical adenocarcinoma (HPV-related)	HeLa	140 (GI_{50})	47
Neuroblastoma	Kelly	330 (GI_{50})	47
Neuroblastoma	IMR-32	380 (GI_{50})	47
Neuroblastoma	SHEP WT	390 (GI_{50})	47
Neuroblastoma	SH-SY5Y	920 (GI_{50})	47
Neuroblastoma	SK-N-SH	4930 (GI_{50})	47
Neuroblastoma	SHEP	9210 (GI_{50})	47

References:

(1) Yang, J.; Ikezoe, T.; Nishioka, C.; Tasaka, T.; Taniguchi, A.; Kuwayama, Y.; Komatsu, N.; Bandobashi, K.; Togitani, K.; Koeffler, H. P.; Taguchi, H.; Yokoyama, A. AZD1152, a Novel and Selective Aurora B Kinase Inhibitor, Induces Growth Arrest, Apoptosis, and Sensitization for Tubulin Depolymerizing Agent or Topoisomerase II Inhibitor in Human Acute Leukemia Cells in Vitro and in Vivo. *Blood* **2007**, *110* (6), 2034–2040. https://doi.org/10.1182/blood-2007-02-073700.

(2) Zekri, A.; Ghaffari, S. H.; Ghanizadeh-Vesali, S.; Yaghmaie, M.; Salmaninejad, A.; Alimoghaddam, K.; Modarressi, M. H.; Ghavamzadeh, A. AZD1152-HQPA Induces Growth Arrest and Apoptosis in Androgen-Dependent Prostate Cancer Cell Line (LNCaP) via Producing Aneugenic Micronuclei and Polyploidy. *Tumour Biol. J. Int. Soc. Oncodevelopmental Biol. Med.* **2015**, *36* (2), 623–632. https://doi.org/10.1007/s13277-014-2664-8.

(3) Borah, N. A.; Sradhanjali, S.; Barik, M. R.; Jha, A.; Tripathy, D.; Kaliki, S.; Rath, S.; Raghav, S. K.; Patnaik, S.; Mittal, R.; Reddy, M. M. Aurora Kinase B Expression, Its Regulation and Therapeutic Targeting in Human Retinoblastoma. *Invest. Ophthalmol. Vis. Sci.* **2021**, *62* (3), 16. https://doi.org/10.1167/iovs.62.3.16.

(4) Lin, Z.-Z.; Jeng, Y.-M.; Hu, F.-C.; Pan, H.-W.; Tsao, H.-W.; Lai, P.-L.; Lee, P.-H.; Cheng, A.-L.; Hsu, H.-C. Significance of Aurora B Overexpression in Hepatocellular Carcinoma. Aurora B Overexpression in HCC. *BMC Cancer* **2010**, *10*, 461. https://doi.org/10.1186/1471-2407-10-461.

(5) Al-Khafaji, A. S.; Davies, M. P.; Risk, J. M.; Marcus, M. W.; Koffa, M.; Gosney, J. R.; Shaw, R. J.; Field, J. K.; Liloglou, T. Aurora B Expression Modulates Paclitaxel Response in Non-Small

Cell Lung Cancer. *Br. J. Cancer* **2017**, *116* (5), 592–599. https://doi.org/10.1038/bjc.2016.453.
(6) Tao, Y.; Zhang, P.; Girdler, F.; Frascogna, V.; Castedo, M.; Bourhis, J.; Kroemer, G.; Deutsch, E. Enhancement of Radiation Response in P53-Deficient Cancer Cells by the Aurora-B Kinase Inhibitor AZD1152. *Oncogene* **2008**, *27* (23), 3244–3255. https://doi.org/10.1038/sj.onc.1210990.
(7) ZHU, X. P.; LIU, Z. L.; PENG, A. F.; ZHOU, Y. F.; LONG, X. H.; LUO, Q. F.; HUANG, S. H.; SHU, Y. Inhibition of Aurora-B Suppresses Osteosarcoma Cell Migration and Invasion. *Exp. Ther. Med.* **2014**, *7* (3), 560–564. https://doi.org/10.3892/etm.2014.1491.
(8) Noronha, S.; Alt, L. A. C.; Scimeca, T. E.; Zarou, O.; Obrzut, J.; Zanotti, B.; Hayward, E. A.; Pillai, A.; Mathur, S.; Rojas, J.; Salamah, R.; Chandar, N.; Fay, M. J. Preclinical Evaluation of the Aurora Kinase Inhibitors AMG 900, AZD1152-HQPA, and MK-5108 on SW-872 and 93T449 Human Liposarcoma Cells. *In Vitro Cell. Dev. Biol. Anim.* **2018**, *54* (1), 71–84. https://doi.org/10.1007/s11626-017-0208-4.
(9) Sánchez-Molina, S.; Figuerola-Bou, E.; Blanco, E.; Sánchez-Jiménez, M.; Táboas, P.; Gómez, S.; Ballaré, C.; García-Domínguez, D. J.; Prada, E.; Hontecillas-Prieto, L.; M Carcaboso, Á.; Tirado, Ó. M.; Hernández-Muñoz, I.; de Álava, E.; Lavarino, C.; Di Croce, L.; Mora, J. RING1B Recruits EWSR1-FLI1 and Cooperates in the Remodeling of Chromatin Necessary for Ewing Sarcoma Tumorigenesis. *Sci. Adv.* **2020**, *6* (43), eaba3058. https://doi.org/10.1126/sciadv.aba3058.
(10) Gully, C. P.; Zhang, F.; Chen, J.; Yeung, J. A.; Velazquez-Torres, G.; Wang, E.; Yeung, S.-C. J.; Lee, M.-H. Antineoplastic Effects of an Aurora B Kinase Inhibitor in Breast Cancer. *Mol. Cancer* **2010**, *9*, 42. https://doi.org/10.1186/1476-4598-9-42.
(11) Baldini, E.; Tuccilli, C.; Prinzi, N.; Sorrenti, S.; Antonelli, A.; Gnessi, L.; Morrone, S.; Moretti, C.; Bononi, M.; Arlot-Bonnemains, Y.; D'Armiento, M.; Ulisse, S. Effects of Selective Inhibitors of Aurora Kinases on Anaplastic Thyroid Carcinoma Cell Lines. *Endocr. Relat. Cancer* **2014**, *21* (5), 797–811. https://doi.org/10.1530/ERC-14-0299.
(12) Tuccilli, C.; Baldini, E.; Prinzi, N.; Morrone, S.; Sorrenti, S.; Filippini, A.; Catania, A.; Alessandrini, S.; Rendina, R.; Coccaro, C.; D'Armiento, M.; Ulisse, S. Preclinical Testing of Selective Aurora Kinase Inhibitors on a Medullary Thyroid Carcinoma-Derived Cell Line. *Endocrine* **2016**, *52* (2), 287–295. https://doi.org/10.1007/s12020-015-0700-0.
(13) Aihara, A.; Tanaka, S.; Yasen, M.; Matsumura, S.; Mitsunori, Y.; Murakata, A.; Noguchi, N.; Kudo, A.; Nakamura, N.; Ito, K.; Arii, S. The Selective Aurora B Kinase Inhibitor AZD1152 as a Novel Treatment for Hepatocellular Carcinoma. *J. Hepatol.* **2010**, *52* (1), 63–71. https://doi.org/10.1016/j.jhep.2009.10.013.
(14) He, J.; Qi, Z.; Zhang, X.; Yang, Y.; Liu, F.; Zhao, G.; Wang, Z. Aurora Kinase B Inhibitor Barasertib (AZD1152) Inhibits Glucose Metabolism in Gastric Cancer Cells. *Anticancer. Drugs* **2019**, *30* (1), 19–26. https://doi.org/10.1097/CAD.0000000000000684.
(15) Wu, Z.-X.; Yang, Y.; Wang, J.-Q.; Zhou, W.-M.; Chen, J.; Fu, Y.-G.; Patel, K.; Chen, Z.-S.; Zhang, J.-Y. Elevated ABCB1 Expression Confers Acquired Resistance to Aurora Kinase Inhibitor GSK-1070916 in Cancer Cells. *Front. Pharmacol.* **2021**, *11*, 615824. https://doi.org/10.3389/fphar.2020.615824.
(16) Hardwicke, M. A.; Oleykowski, C. A.; Plant, R.; Wang, J.; Liao, Q.; Moss, K.; Newlander, K.; Adams, J. L.; Dhanak, D.; Yang, J.; Lai, Z.; Sutton, D.; Patrick, D. GSK1070916, a Potent Aurora B/C Kinase Inhibitor with Broad Antitumor Activity in Tissue Culture Cells and Human Tumor Xenograft Models. *Mol. Cancer Ther.* **2009**, *8* (7), 1808–1817. https://doi.org/10.1158/1535-7163.MCT-09-0041.
(17) Benten, D.; Keller, G.; Quaas, A.; Schrader, J.; Gontarewicz, A.; Balabanov, S.; Braig, M.; Wege, H.; Moll, J.; Lohse, A. W.; Brummendorf, T. H. Aurora Kinase Inhibitor PHA-739358 Suppresses Growth of Hepatocellular Carcinoma In Vitro and in a Xenograft Mouse Model. *Neoplasia N. Y. N* **2009**, *11* (9), 934–944.
(18) Zhang, Y.; Zhang, B.; Feng, W.; Li, Y.; Cao, X. [Molecular mechanism of Aurora kinase inhibitor PHA739358 in inhibited proliferation and induced apoptosis of breast cancer cells]. *Zhonghua Yi Xue Za Zhi* **2012**, *92* (1), 45–49.

(19) Xie, L.; Meyskens, F. L. The Pan-Aurora Kinase Inhibitor, PHA-739358, Induces Apoptosis and Inhibits Migration in Melanoma Cell Lines. *Melanoma Res.* **2013**, *23* (2), 102–113. https://doi.org/10.1097/CMR.0b013e32835df5e4.
(20) He, S.-J.; Shu, L.-P.; Zhou, Z.-W.; Yang, T.; Duan, W.; Zhang, X.; He, Z.-X.; Zhou, S.-F. Inhibition of Aurora Kinases Induces Apoptosis and Autophagy via AURKB/P70S6K/RPL15 Axis in Human Leukemia Cells. *Cancer Lett.* **2016**, *382* (2), 215–230. https://doi.org/10.1016/j.canlet.2016.08.016.
(21) Li, J.-P.; Yang, Y.-X.; Liu, Q.-L.; Zhou, Z.-W.; Pan, S.-T.; He, Z.-X.; Zhang, X.; Yang, T.; Pan, S.-Y.; Duan, W.; He, S.-M.; Chen, X.-W.; Qiu, J.-X.; Zhou, S.-F. The Pan-Inhibitor of Aurora Kinases Danusertib Induces Apoptosis and Autophagy and Suppresses Epithelial-to-Mesenchymal Transition in Human Breast Cancer Cells. *Drug Des. Devel. Ther.* **2015**, *9*, 1027–1062. https://doi.org/10.2147/DDDT.S74412.
(22) Zi, D.; Zhou, Z.-W.; Yang, Y.-J.; Huang, L.; Zhou, Z.-L.; He, S.-M.; He, Z.-X.; Zhou, S.-F. Danusertib Induces Apoptosis, Cell Cycle Arrest, and Autophagy but Inhibits Epithelial to Mesenchymal Transition Involving PI3K/Akt/MTOR Signaling Pathway in Human Ovarian Cancer Cells. *Int. J. Mol. Sci.* **2015**, *16* (11), 27228–27251. https://doi.org/10.3390/ijms161126018.
(23) Balabanov, S.; Gontarewicz, A.; Keller, G.; Raddrizzani, L.; Braig, M.; Bosotti, R.; Moll, J.; Jost, E.; Barett, C.; Rohe, I.; Bokemeyer, C.; Holyoake, T. L.; Brümmendorf, T. H. Abcg2 Overexpression Represents a Novel Mechanism for Acquired Resistance to the Multi-Kinase Inhibitor Danusertib in BCR-ABL-Positive Cells In Vitro. *PLoS ONE* **2011**, *6* (4), e19164. https://doi.org/10.1371/journal.pone.0019164.
(24) Zhu, Q.; Yu, X.; Zhou, Z.-W.; Luo, M.; Zhou, C.; He, Z.-X.; Chen, Y.; Zhou, S.-F. A Quantitative Proteomic Response of Hepatocellular Carcinoma Hep3B Cells to Danusertib, a Pan-Aurora Kinase Inhibitor. *J. Cancer* **2018**, *9* (12), 2061–2071. https://doi.org/10.7150/jca.20822.
(25) Yuan, C.-X.; Zhou, Z.-W.; Yang, Y.-X.; He, Z.-X.; Zhang, X.; Wang, D.; Yang, T.; Pan, S.-Y.; Chen, X.-W.; Zhou, S.-F. Danusertib, a Potent Pan-Aurora Kinase and ABL Kinase Inhibitor, Induces Cell Cycle Arrest and Programmed Cell Death and Inhibits Epithelial to Mesenchymal Transition Involving the PI3K/Akt/MTOR-Mediated Signaling Pathway in Human Gastric Cancer AGS and NCI-N78 Cells. *Drug Des. Devel. Ther.* **2015**, *9*, 1293–1318. https://doi.org/10.2147/DDDT.S74964.
(26) Shang, Y.-Y.; Yu, N.; Xia, L.; Yu, Y.-Y.; Ma, C.; Jiao, Y.-N.; Li, Y.; Wang, Y.; Dang, J.; Li, W. Augmentation of Danusertib's Anticancer Activity against Melanoma by Blockage of Autophagy. *Drug Deliv. Transl. Res.* **2020**, *10* (1), 136–145. https://doi.org/10.1007/s13346-019-00668-5.
(27) Tanaka, R.; Squires, M. S.; Kimura, S.; Yokota, A.; Nagao, R.; Yamauchi, T.; Takeuchi, M.; Yao, H.; Reule, M.; Smyth, T.; Lyons, J. F.; Thompson, N. T.; Ashihara, E.; Ottmann, O. G.; Maekawa, T. Activity of the Multitargeted Kinase Inhibitor, AT9283, in Imatinib-Resistant BCR-ABL–Positive Leukemic Cells. *Blood* **2010**, *116* (12), 2089–2095. https://doi.org/10.1182/blood-2009-03-211466.
(28) Qi, W.; Liu, X.; Cooke, L. S.; Persky, D. O.; Miller, T. P.; Squires, M.; Mahadevan, D. AT9283, a Novel Aurora Kinase Inhibitor, Suppresses Tumor Growth in Aggressive B-Cell Lymphomas. *Int. J. Cancer* **2012**, *130* (12), 2997–3005. https://doi.org/10.1002/ijc.26324.
(29) Kalous, O.; Conklin, D.; Desai, A. J.; Dering, J.; Goldstein, J.; Ginther, C.; Anderson, L.; Lu, M.; Kolarova, T.; Eckardt, M. A.; Langerød, A.; Børresen-Dale, A.-L.; Slamon, D. J.; Finn, R. S. AMG 900, Pan-Aurora Kinase Inhibitor, Preferentially Inhibits the Proliferation of Breast Cancer Cell Lines with Dysfunctional P53. *Breast Cancer Res. Treat.* **2013**, *141* (3), 397–408. https://doi.org/10.1007/s10549-013-2702-z.
(30) Payton, M.; Bush, T. L.; Chung, G.; Ziegler, B.; Eden, P.; McElroy, P.; Ross, S.; Cee, V. J.; Deak, H. L.; Hodous, B. L.; Nguyen, H. N.; Olivieri, P. R.; Romero, K.; Schenkel, L. B.; Bak, A.; Stanton, M.; Dussault, I.; Patel, V. F.; Geuns-Meyer, S.; Radinsky, R.; Kendall, R. L. Preclinical Evaluation of AMG 900, a Novel Potent and Highly Selective Pan-Aurora Kinase Inhibitor with

Activity in Taxane-Resistant Tumor Cell Lines. *Cancer Res.* **2010**, *70* (23), 9846–9854. https://doi.org/10.1158/0008-5472.CAN-10-3001.
(31) Lee, H.-A.; Kwon, M.; Kim, H.-A.; Kim, K.-S. Antitumor Profiles and Cardiac Electrophysiological Effects of Aurora Kinase Inhibitor ZM447439. *Korean J. Physiol. Pharmacol.* **2019**, *23* (5), 393–402. https://doi.org/10.4196/kjpp.2019.23.5.393.
(32) Shiomitsu, K.; Xia, X.; Waite, K.; Sehgal, I.; Li, S. Evaluation of the Aurora Kinase Inhibitor, ZM447439, in Canine Malignant Lymphoid Cells *in Vitro*. **2013**, *2013*. https://doi.org/10.4236/ojvm.2013.31006.
(33) Tavanti, E.; Sero, V.; Vella, S.; Fanelli, M.; Michelacci, F.; Landuzzi, L.; Magagnoli, G.; Versteeg, R.; Picci, P.; Hattinger, C. M.; Serra, M. Preclinical Validation of Aurora Kinases-Targeting Drugs in Osteosarcoma. *Br. J. Cancer* **2013**, *109* (10), 2607–2618. https://doi.org/10.1038/bjc.2013.643.
(34) Vidarsdottir, L.; Steingrimsdottir, G.; Bodvarsdottir, S. K.; Ogmundsdottir, H. M.; Eyfjord, J. E. Sensitivity of BRCA2 Mutated Human Cell Lines to Aurora Kinase Inhibition. *Invest. New Drugs* **2012**, *30* (2), 425–434. https://doi.org/10.1007/s10637-010-9566-4.
(35) Wang, S.; Midgley, C. A.; Scaërou, F.; Grabarek, J. B.; Griffiths, G.; Jackson, W.; Kontopidis, G.; McClue, S. J.; McInnes, C.; Meades, C.; Mezna, M.; Plater, A.; Stuart, I.; Thomas, M. P.; Wood, G.; Clarke, R. G.; Blake, D. G.; Zheleva, D. I.; Lane, D. P.; Jackson, R. C.; Glover, D. M.; Fischer, P. M. Discovery of N-Phenyl-4-(Thiazol-5-Yl)Pyrimidin-2-Amine Aurora Kinase Inhibitors. *J. Med. Chem.* **2010**, *53* (11), 4367–4378. https://doi.org/10.1021/jm901913s.
(36) Le, T. T. D.; Ha, P. T.; Tran, T. H. Y.; Nguyen, D. T.; Nguyen, H. N.; Bui, V. K.; Hoang, M. N. $\less$i$\greater$In Vitro$\less$/I$\greater$ Evaluation of Aurora Kinase Inhibitor—VX680—in Formulation of PLA-TPGS Nanoparticles. *Adv. Nat. Sci. Nanosci. Nanotechnol.* **2016**, *7* (2), 025010. https://doi.org/10.1088/2043-6262/7/2/025010.
(37) Arbitrario, J. P.; Belmont, B. J.; Evanchik, M. J.; Flanagan, W. M.; Fucini, R. V.; Hansen, S. K.; Harris, S. O.; Hashash, A.; Hoch, U.; Hogan, J. N.; Howlett, A. R.; Jacobs, J. W.; Lam, J. W.; Ritchie, S. C.; Romanowski, M. J.; Silverman, J. A.; Stockett, D. E.; Teague, J. N.; Zimmerman, K. M.; Taverna, P. SNS-314, a Pan-Aurora Kinase Inhibitor, Shows Potent Anti-Tumor Activity and Dosing Flexibility in Vivo. *Cancer Chemother. Pharmacol.* **2010**, *65* (4), 707–717. https://doi.org/10.1007/s00280-009-1076-8.
(38) Sini, P.; Gürtler, U.; Zahn, S. K.; Baumann, C.; Rudolph, D.; Baumgartinger, R.; Strauss, E.; Haslinger, C.; Tontsch-Grunt, U.; Waizenegger, I. C.; Solca, F.; Bader, G.; Zoephel, A.; Treu, M.; Reiser, U.; Garin-Chesa, P.; Boehmelt, G.; Kraut, N.; Quant, J.; Adolf, G. R. Pharmacological Profile of BI 847325, an Orally Bioavailable, ATP-Competitive Inhibitor of MEK and Aurora Kinases. *Mol. Cancer Ther.* **2016**, *15* (10), 2388–2398. https://doi.org/10.1158/1535-7163.MCT-16-0066.
(39) Phadke, M. S.; Sini, P.; Smalley, K. S. M. The Novel ATP-Competitive MEK/Aurora Kinase Inhibitor BI-847325 Overcomes Acquired BRAF Inhibitor Resistance through Suppression of Mcl-1 and MEK Expression. *Mol. Cancer Ther.* **2015**, *14* (6), 1354–1364. https://doi.org/10.1158/1535-7163.MCT-14-0832.
(40) He, Y.; Fu, W.; Du, L.; Yao, H.; Hua, Z.; Li, J.; Lin, Z. Discovery of a Novel Aurora B Inhibitor GSK650394 with Potent Anticancer and Anti-Aspergillus Fumigatus Dual Efficacies in Vitro. *J. Enzyme Inhib. Med. Chem.* **2022**, *37* (1), 109–117. https://doi.org/10.1080/14756366.2021.1975693.
(41) Lakkaniga, N. R.; Zhang, L.; Belachew, B.; Gunaganti, N.; Frett, B.; Li, H. Discovery of SP-96, the First Non-ATP-Competitive Aurora Kinase B Inhibitor, for Reduced Myelosuppression. *Eur. J. Med. Chem.* **2020**, *203*, 112589. https://doi.org/10.1016/j.ejmech.2020.112589.
(42) Soncini, C.; Carpinelli, P.; Gianellini, L.; Fancelli, D.; Vianello, P.; Rusconi, L.; Storici, P.; Zugnoni, P.; Pesenti, E.; Croci, V.; Ceruti, R.; Giorgini, M. L.; Cappella, P.; Ballinari, D.; Sola, F.; Varasi, M.; Bravo, R.; Moll, J. PHA-680632, a Novel Aurora Kinase Inhibitor with Potent Antitumoral Activity. *Clin. Cancer Res.* **2006**, *12* (13), 4080–4089. https://doi.org/10.1158/1078-

0432.CCR-05-1964.
(43) Chan, F.; Sun, C.; Perumal, M.; Nguyen, Q.-D.; Bavetsias, V.; McDonald, E.; Martins, V.; Wilsher, N. E.; Raynaud, F. I.; Valenti, M.; Eccles, S.; te Poele, R.; Workman, P.; Aboagye, E. O.; Linardopoulos, S. Mechanism of Action of the Aurora Kinase Inhibitor CCT129202 and in Vivo Quantification of Biological Activity. *Mol. Cancer Ther.* **2007**, *6* (12), 3147–3157. https://doi.org/10.1158/1535-7163.MCT-07-2156.
(44) Cheng, C.; Liu, Z.; Zhang, H.; Xie, J.; Chen, X.; Zhao, X.; Wang, F.; Liang, Y.; Chen, L.; Singh, S.; Chen, J.; Talele, T. T.; Chen, Z.; Zhong, F.; Fu, L. Enhancing Chemosensitivity in ABCB1- and ABCG2-Overexpressing Cells and Cancer Stem-like Cells by An Aurora Kinase Inhibitor CCT129202. *Mol. Pharm.* **2012**, *9* (7), 1971–1982. https://doi.org/10.1021/mp2006714.
(45) Huang, D.; Huang, Y.; Huang, Z.; Weng, J.; Zhang, S.; Gu, W. Relation of AURKB Over-Expression to Low Survival Rate in BCRA and Reversine-Modulated Aurora B Kinase in Breast Cancer Cell Lines. *Cancer Cell Int.* **2019**, *19* (1), 166. https://doi.org/10.1186/s12935-019-0885-z.
(46) Lu, Y.-C.; Lee, Y.-R.; Liao, J.-D.; Lin, C.-Y.; Chen, Y.-Y.; Chen, P.-T.; Tseng, Y.-S. Reversine Induced Multinucleated Cells, Cell Apoptosis and Autophagy in Human Non-Small Cell Lung Cancer Cells. *PLOS ONE* **2016**, *11* (7), e0158587. https://doi.org/10.1371/journal.pone.0158587.
(47) Faisal, A.; Vaughan, L.; Bavetsias, V.; Sun, C.; Atrash, B.; Avery, S.; Jamin, Y.; Robinson, S. P.; Workman, P.; Blagg, J.; Raynaud, F. I.; Eccles, S. A.; Chesler, L.; Linardopoulos, S. The Aurora Kinase Inhibitor CCT137690 Downregulates MYCN and Sensitizes MYCN-Amplified Neuroblastoma In Vivo. *Mol. Cancer Ther.* **2011**, *10* (11), 2115–2123. https://doi.org/10.1158/1535-7163.MCT-11-0333.
